# Supplementary material for: Leveraging DNA-Methylation Quantitative-Trait Loci to Characterize the Relationship between Methylomic Variation, Gene Expression, and Complex Traits
Source: Am J Hum Genet. 2018 Oct 25;103(5):654–65. doi: 10.1016/j.ajhg.2018.09.007 (PMC6217758; doi:10.1016/j.ajhg.2018.09.007)
Supplement: Document S1. Figures S1–S17 and Tables S1–S3 and S9 [file mmc1.pdf]

The American Journal of Human Genetics, Volume *103*

Supplemental Data

Leveraging DNA-Methylation Quantitative-Trait Loci to Characterize the Relationship between Methylomic Variation, Gene Expression, and Complex Traits

Eilis Hannon, Tyler J. Gorrie-Stone, Melissa C. Smart, Joe Burrage, Amanda Hughes, Yanchun Bao, Meena Kumari, Leonard C. Schalkwyk, and Jonathan Mill

Figure S1: Overview of the study providing a schematic of our analytical plan and the datasets used in analyses.

Overview of analytical plan and datasets.

Identification of DNA methylation quantitative trait loci (mQTL)

Data:
Understanding Society UK Household Longitudinal study (UKHLS) (n = 1,111)
(https://www.understandingsociety.ac.uk/)
    Illumina EPIC array (766,714 DNA methylation sites)
    Imputed genotypes (5,210,475 genetic variants)
Analytical software:
    Matrix EQTL (http://www.bios.unc.edu/research/genomic_software/Matrix_eQTL/)

Aim 1: Refinement of genetic association signals from GWAS analyses

Data:
Publically available GWAS results
    (see **Supplementary Table 5**)
Westra et al (n = 5,111)
(https://molgenis58.target.rug.nl/bloodeqtlbrowser/)
    blood *cis* eQTL results
Analytical software:
    SMR (http://cnsgenomics.com/software/smr/)

Aim 2: Identification of associations between DNAm and gene expression:

Data:
Westra et al (n = 5,111)
(https://molgenis58.target.rug.nl/bloodeqtlbrowser/)
    blood *cis* eQTL results
Analytical software:
    SMR (http://cnsgenomics.com/software/smr/)

Figure S2: Genic distribution of novel associations between genetic variation and DNA methylation using the Illumina EPIC array. Barplot demonstrating the genic feature annotation of DNA methylation sites associated with common genetic variation split by whether the DNA methylation site was part of the older 450K array (red) or novel to the EPIC array (blue).

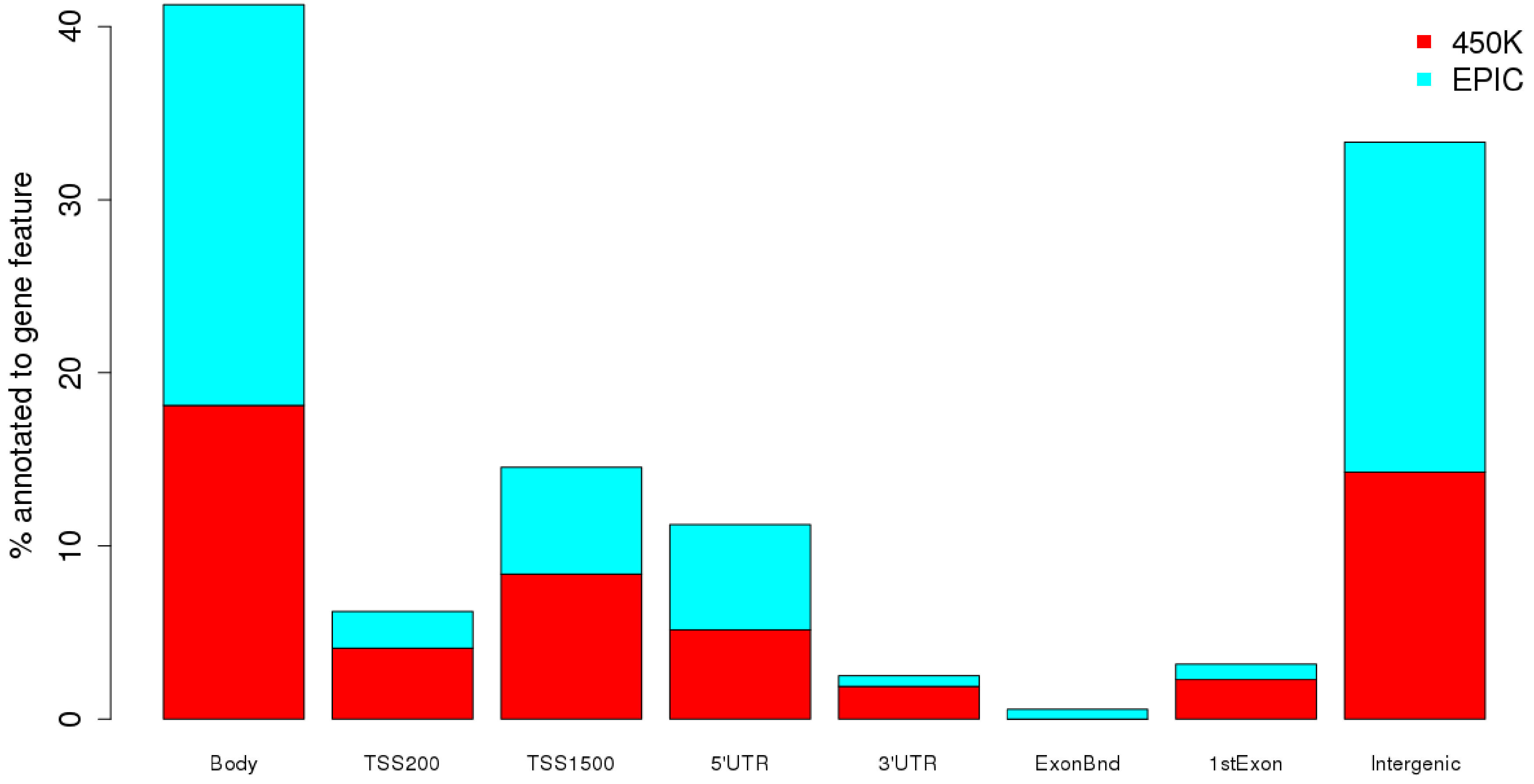

Figure S3: Additional content on the Illumina EPIC array enable the identification of novel DNA methylation quantitative trait loci (mQTL) associations. Venn diagram of the overlap of genes annotated to genetically influenced DNA methylation sites (identified at $P < 6.52x10^{-14}$) through content included on the 450K array and novel content added to the EPIC array. The outside circle includes all genes annotated to any DNA methylation site tested.

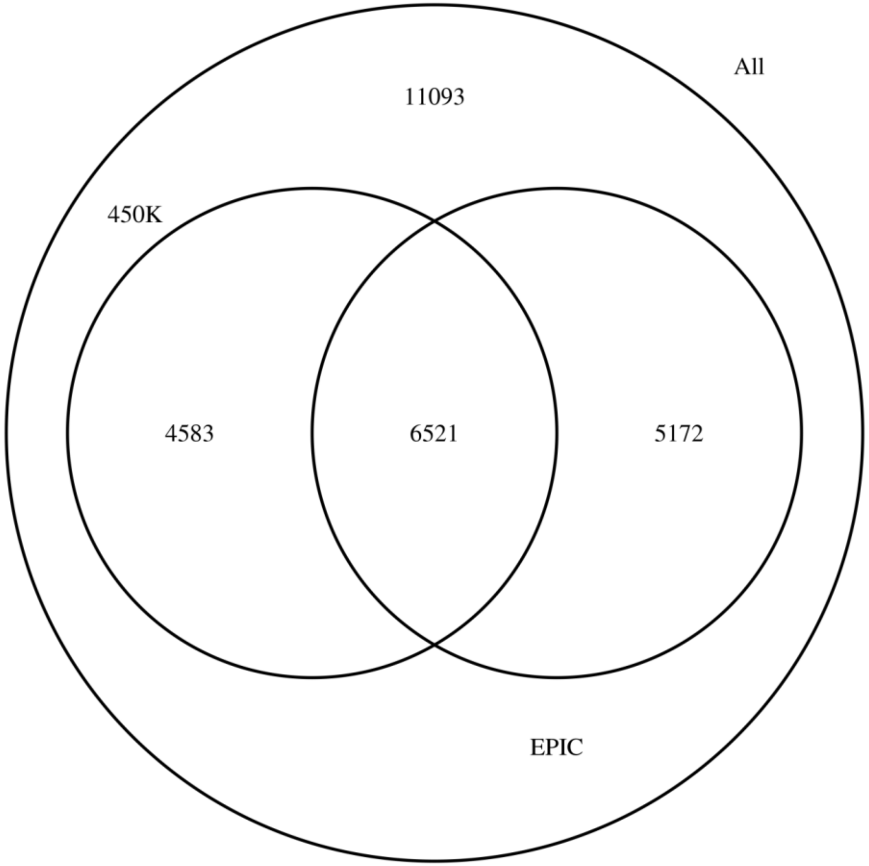

Figure S4: Frequency distribution of DNA methylation quantitative trait loci (mQTL) SNPs and their associated DNA methylation sites. Histograms of **A**) the number of independent genetic variants an individual DNA methylation site is associated with and **B**) the number of DNA methylation sites a genetic variant is associated with. Genetically-mediated DNA methylation sites are often associated with multiple mQTL SNPs (likely reflecting an artefact of linkage disequilibrium), but mQTL SNPs are associated with DNA methylation at relatively few sites.

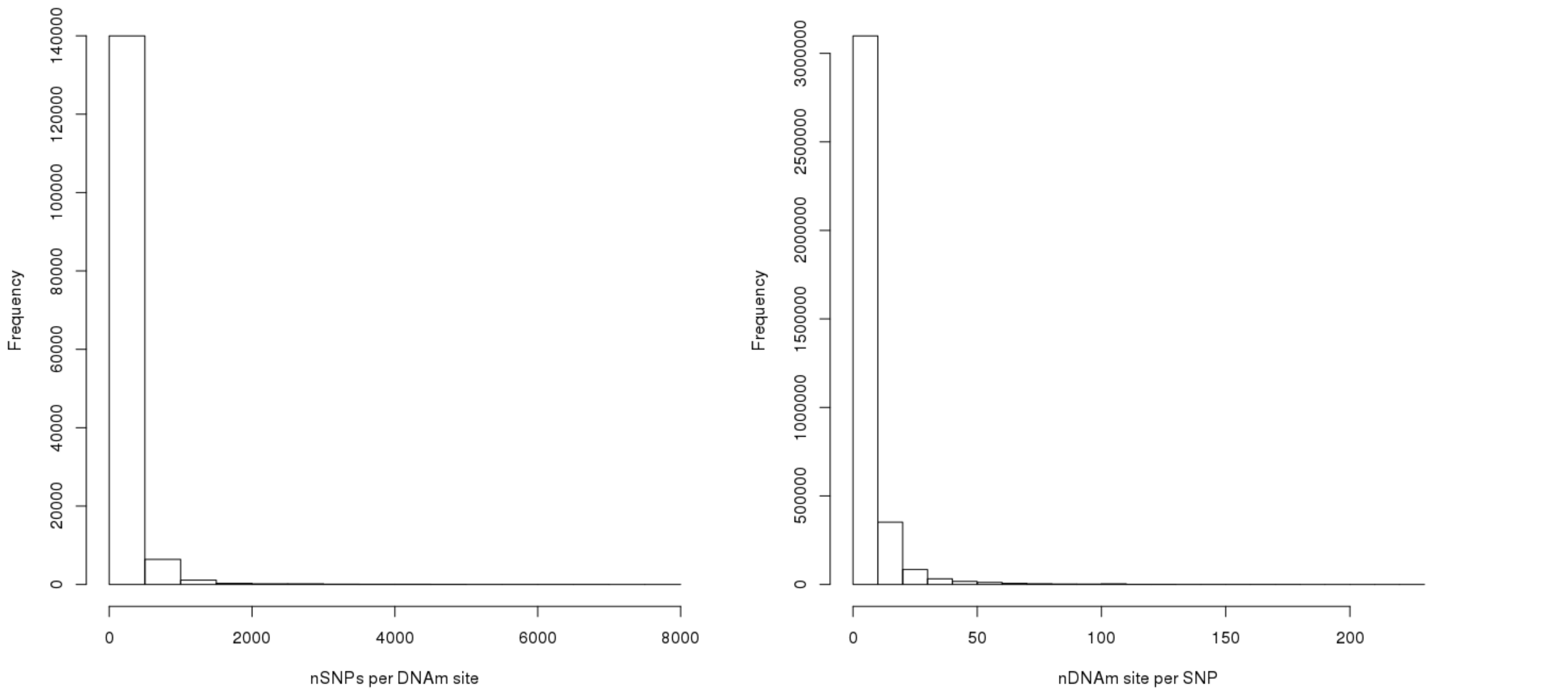

Figure S5: Frequency distribution of DNA methylation quantitative trait loci (mQTL) SNPs and associated DNA methylation sites after filtering for independent associations. Histograms of **A**) the number of independent genetic variants a DNA methylation site is associated with and **B**) the number of DNA methylation sites a genetic variant is associated with. Independent genetic signals were identified by clumping DNA methylation quantitative trait loci with a Bonferroni corrected P value of $P < 6.52 \times 10^{-14}$. After controlling for correlated genetic signals, each DNA methylation site is associated with a median of 1 genetic variant, and each genetic variant is associated with a median of 1 DNA methylation site.

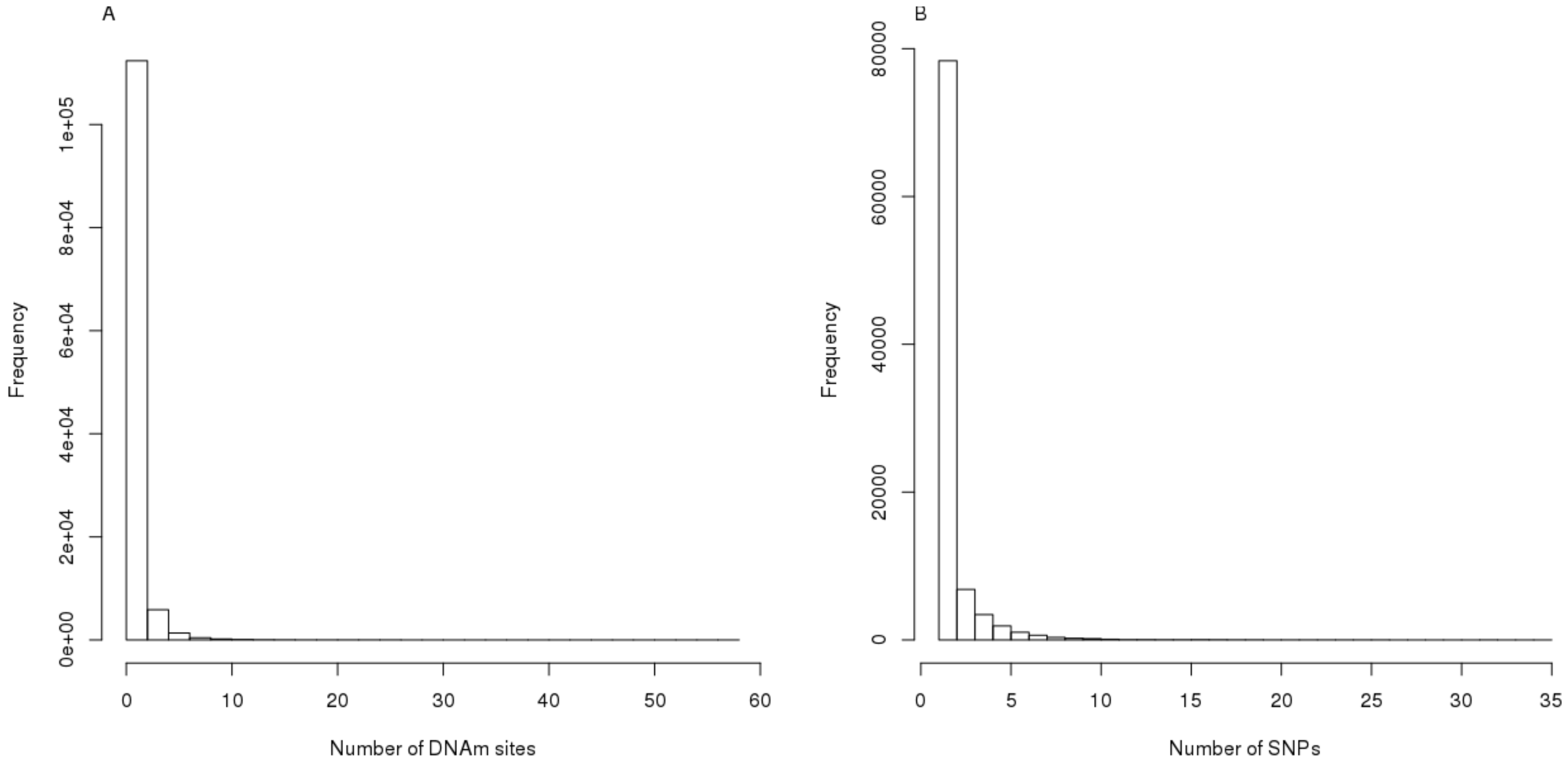

Figure S6: The distribution of effect sizes across all Bonferroni significant DNA methylation quantitative trait loci (mQTL). Shown is the DNA methylation difference (% DNA methylation) associated with each minor allele for all mQTLs ($P < 6.52x10^{-14}$), *cis*-acting mQTLs, and *trans*-acting mQTLs. The average effect size for *trans*-mQTLs (3.26% (SD = 2.78%) per allele) is significantly lower than that observed for *cis*-mQTLs (3.48% (SD = 3.03%) per allele) (two-sided Wilcoxon rank sum test, $P = 1.69x10^{-19}$).

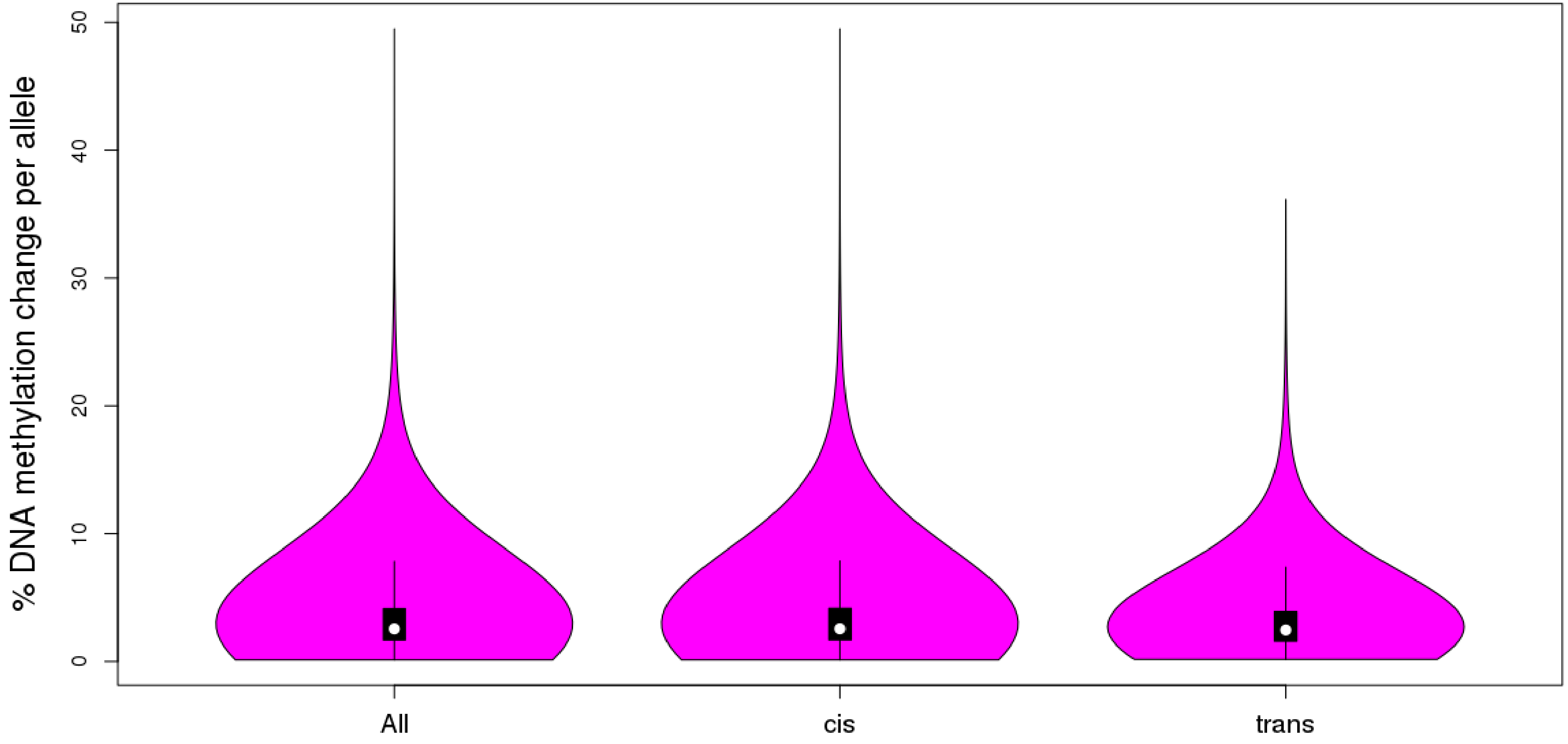

Figure S7: The significance and effect size of cis mQTL associations increases as the distance between the genetic variant and DNAm site decreases. Shown is the relationship between A) mQTL significance (–$\log_{10}$ P value) and B) the mean change in DNA methylation (%) per allele and distance between the DNA methylation site and genetic variant. The color of the points reflects the density of observations at that location, with gray indicating smallest density and red the highest.

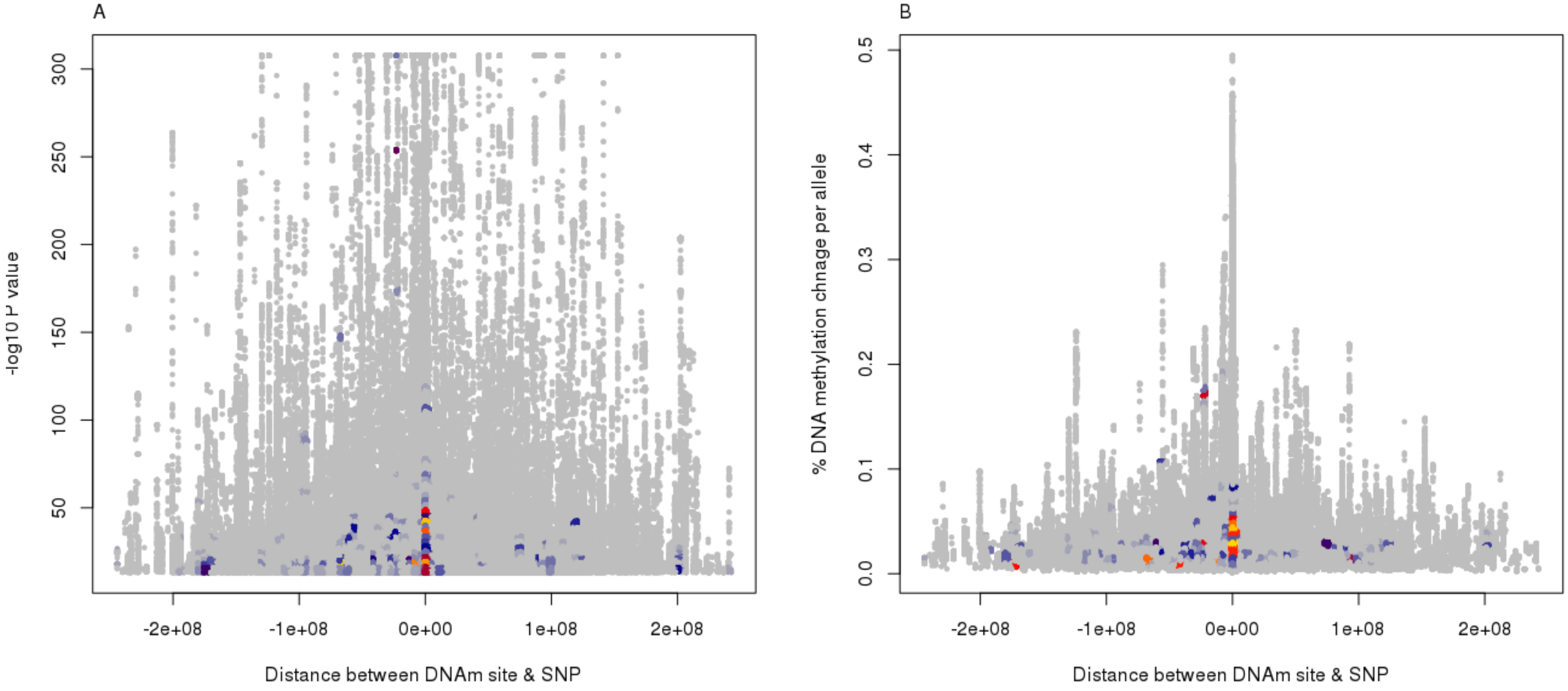

Figure S8: Genic distribution of DNA methylation sites associated with mQTL variation. Bar-plot demonstrating the genic location of DNA methylation sites associated with common genetic variation ($P < 6.52 \times 10^{-14}$; blue bars) compared to the background distribution of all DNA methylation sites tested in the mQTL analysis (gray bars). DNA methylation sites associated with mQTLs are significantly enriched in intergenic regions, and significantly depleted in both the gene body and 1st Exon.

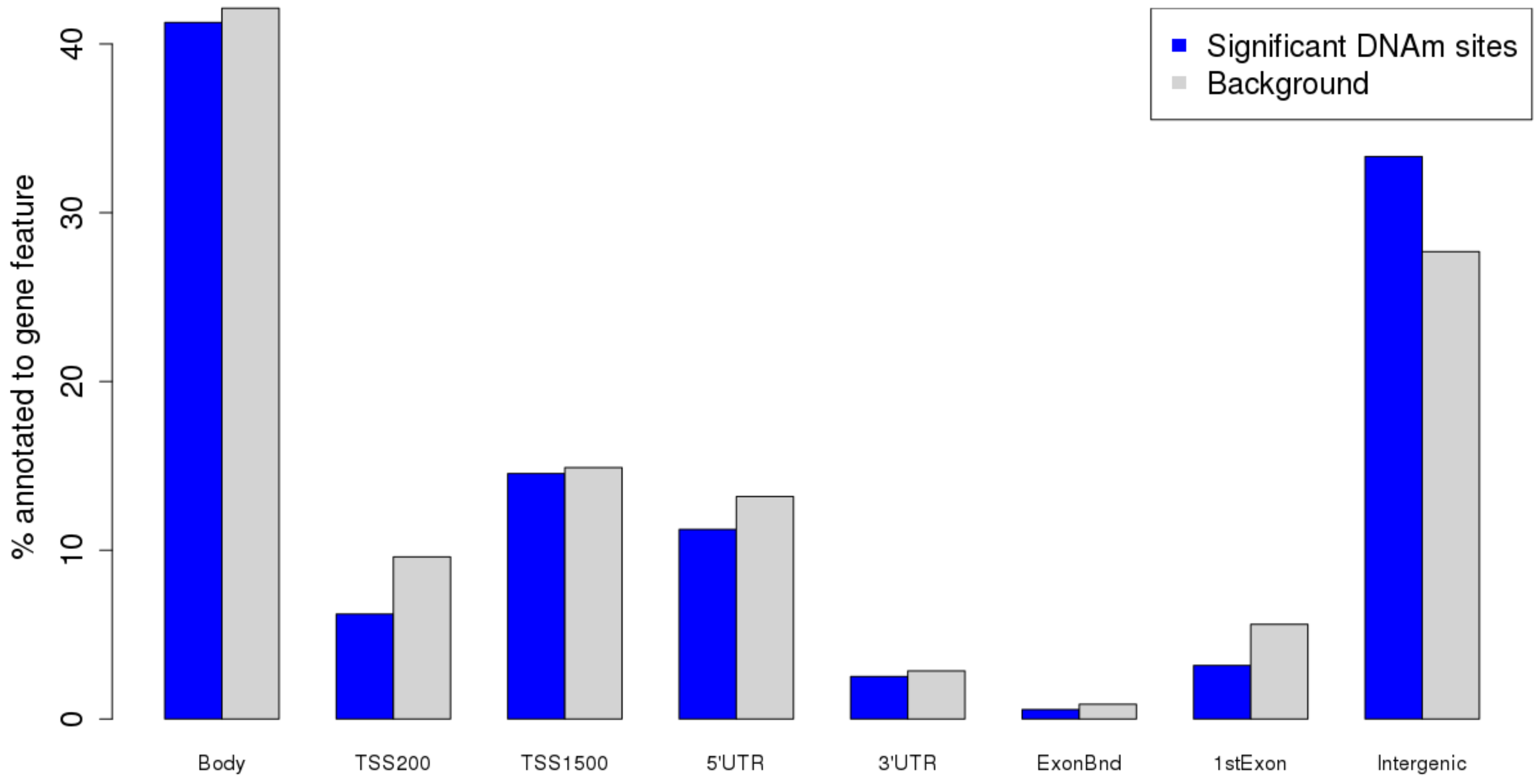

Figure S9: Distribution of DNA methylation sites associated with mQTL variation in CpG island features. Bar-plot demonstrating the genic location of DNA methylation sites associated with common genetic variation ($P < 6.52 \times 10^{-14}$; blue bars) compared to the background distribution of all DNA methylation sites tested in the mQTL analysis (gray bars). DNA methylation sites associated with mQTLs are significantly enriched in CpG shores and regions classified as “open sea”, and significantly depleted in CpG islands

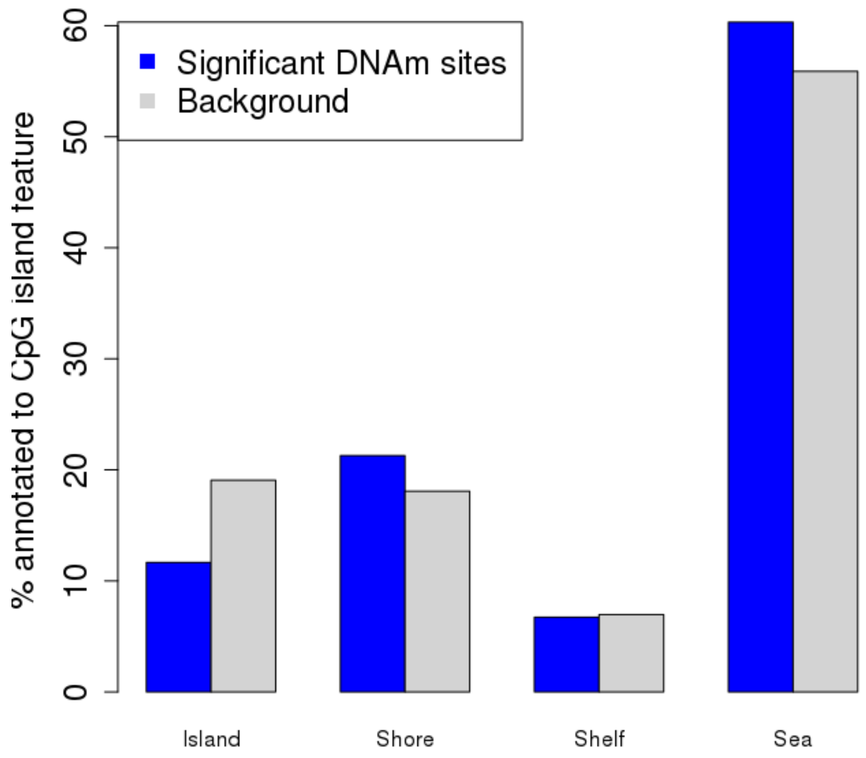

Figure S10: DNA methylation sites associated with common mQTL variants are more strongly influenced by additive genetic variation. Density plots of the distribution of percentage of variance in DNA methylation explained by genetic factors (h^2) as reported by (van Dongen et al. 2016). The distribution of all DNA methylation sites tested (black line), all DNA methylation sites with a Bonferroni significant association ($P < 6.52 \times 10^{-14}$; red line), DNA methylation sites identified with a *cis* acing genetic association (blue line) and DNA methylation sites identified with a *trans* acting genetic association (green line). *cis* effects are defined as those where the DNA methylation site and genetic variants are located within 500kilbases on the same chromosome.

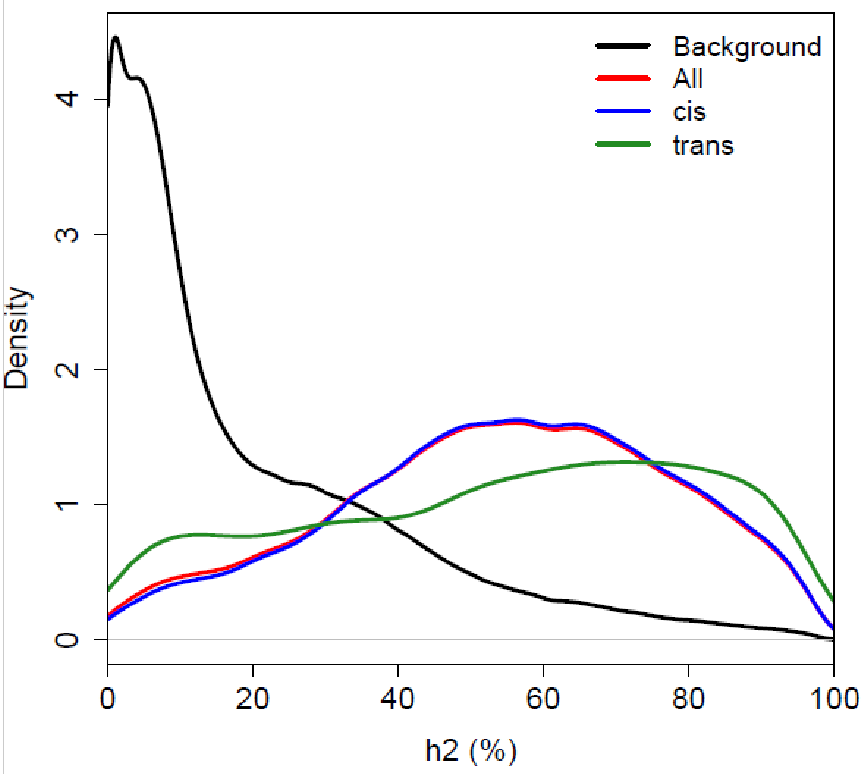

Figure S11: Distribution of correlation coefficients between pairs of DNAm sites with shared genetic effects. Histogram of correlation coefficients between pairs of DNAm sites with convincing evidence for co-localisation of genetic associations (n = 234,460 pairs with $PP_3 + PP_4 > 0.99$ & $PP_4/PP_3 > 5$). These pairs are enriched for concordant directions of effects (71.2% pairs with positive correlations vs 28.8% pairs with negative correlations, binomial test P = $1.48x10^{-323}$).

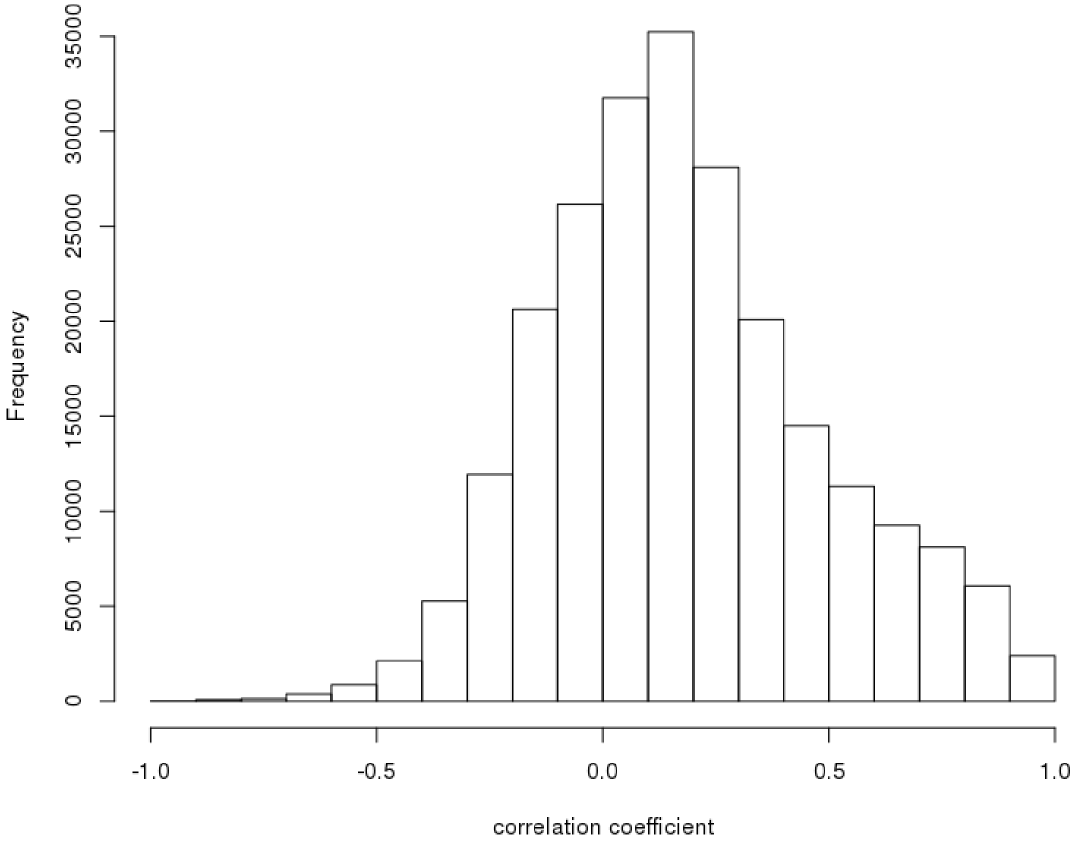

Figure S12: Distribution of correlation coefficients between pairs of DNAm sites with shared genetic effects, split by genic feature annotation. Boxplots of correlation coefficients between pairs of DNAm sites split by the genic feature annotation of the two DNAm sites in the pair for A) all pairs with convincing evidence for co-localisation of genetic associations (n = 234,460 pairs with $PP_3 + PP_4 > 0.99$ & $PP_4/PP_3 > 5$) and B) the subset of convincing pairs annotated to the same gene (n = 83,416). The colour of the boxplot indicates the genic feature category of one DNAm site in the pair and the location of the box indicates the genic feature category of the second DNam site. For example, the first boxplot on the far left of the figure presents the distribution of all pairs of DNAm sites where both sites are located in the 1st exon (indicated by the location in the 1st Exon panel and the colour red), the second yellow boxplot from the left presents the distribution of all pairs of DNAm sites where one DNAm site is located in the 1st Exon (indicated by the location in the 1st Exon panel) and the second is in the 3'UTR (indicated by the colour). In general we observe that where both DNAm sites are annotated the same genic feature the correlations are more likely to be positive (OR = 1.73, fisher's $P < 2.2x10^{-16}$) and higher (Mann-Whitney test $P < 2.2x10^{-16}$) compared to pairs annotated to different genic features.

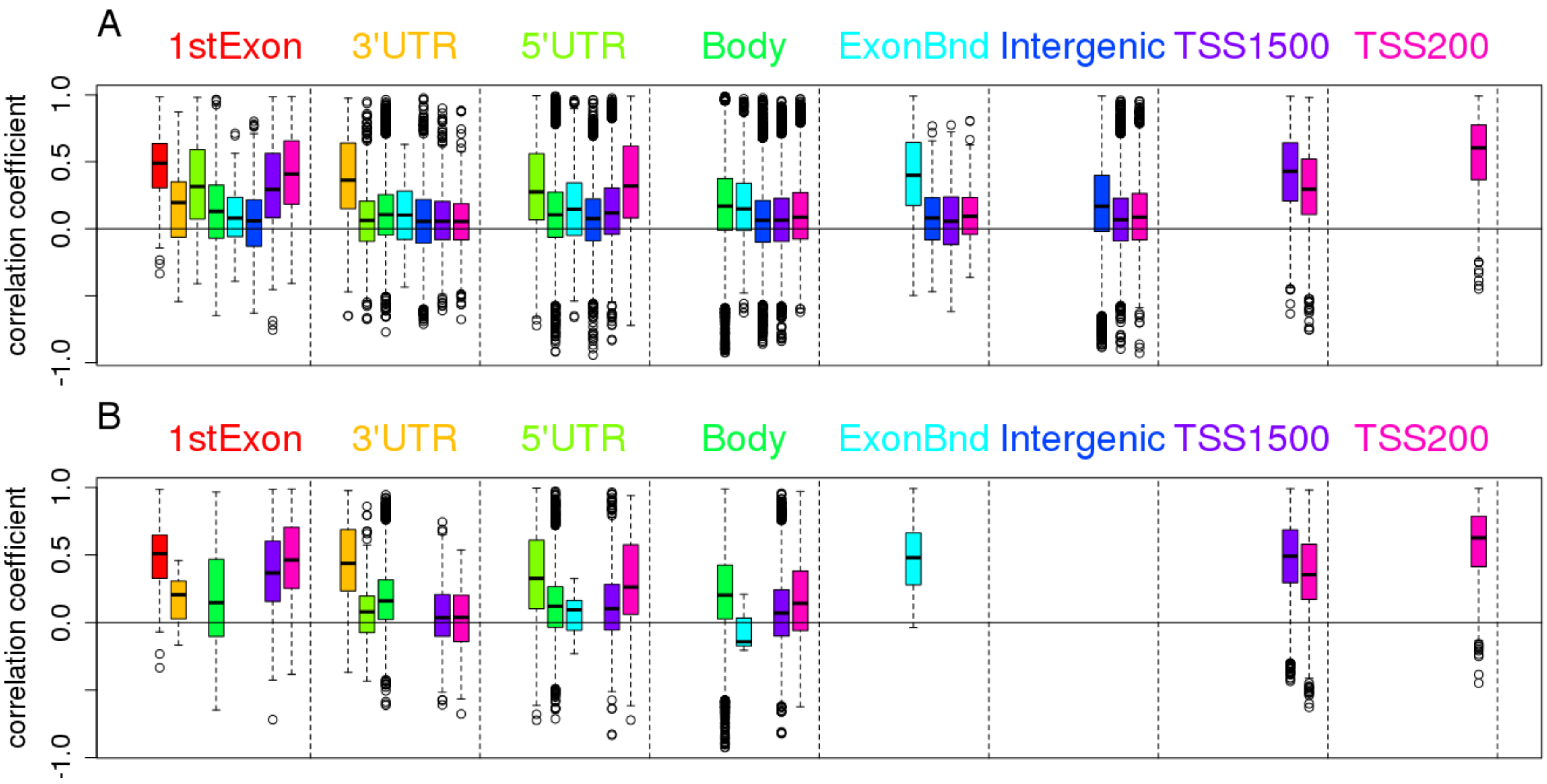
A
1stExon
3'UTR
5'UTR
Body
ExonBnd
Intergenic
TSS1500
TSS200
correlation coefficient
1.0
0.5
0.0
-1.0
B
1stExon
3'UTR
5'UTR
Body
ExonBnd
Intergenic
TSS1500
TSS200
correlation coefficient
1.0
0.5
0.0
-1.0

Figure S13: Manhattan plots of Summary data-based Mendelian Randomisation (SMR) tests for pleiotropic effects between 63 complex traits and DNA methylation. Shown on the y-axis of each plot is the –log10 P-value from the SMR analysis using DNA methylation quantitative trait loci (mQTL) generated from whole blood. Each point represents an SMR test for a particular DNA methylation site. The red horizontal line represents the genome-wide multiple testing significance threshold ($P < 6.42 \times 10^{-7}$); green points highlight the significant SMR tests which are not characterized by significant heterogeneity (i.e. $P > 0.05$), indicating pleiotropic relationships between that trait and either DNA methylation.

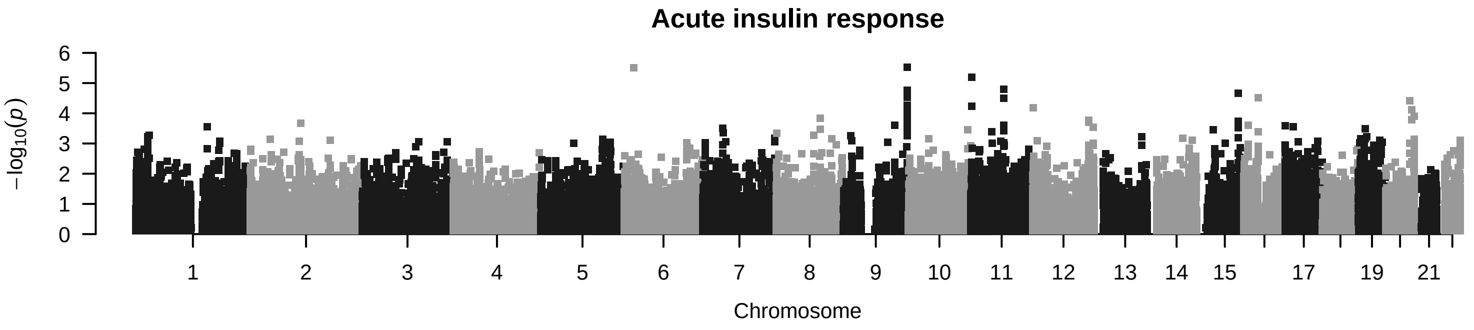
Acute insulin response
−log10(p)
0
1
2
3
4
5
6
1
2
3
4
5
6
7
8
9
10
11
12
13
14
15
17
19
21
Chromosome

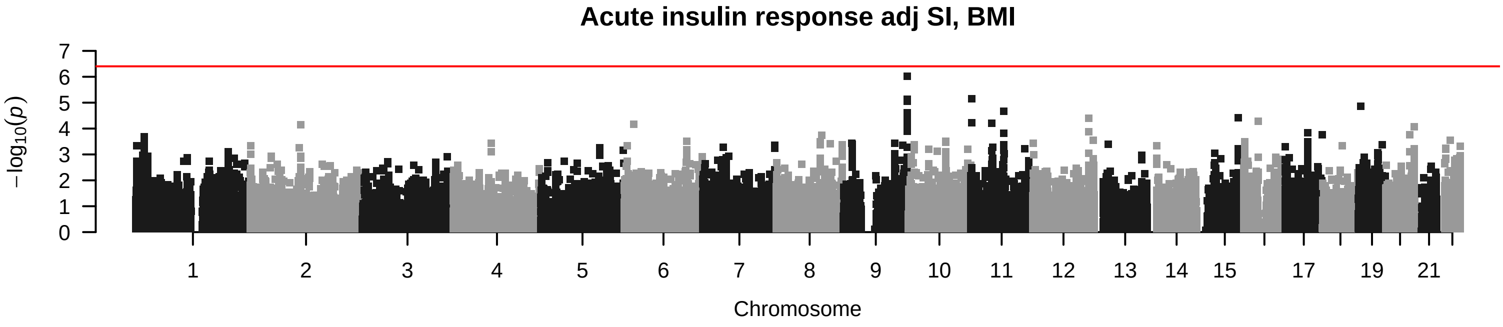
Acute insulin response adj SI, BMI
−log10(p)
0
1
2
3
4
5
6
7
1
2
3
4
5
6
7
8
9
10
11
12
13
14
15
17
19
21
Chromosome

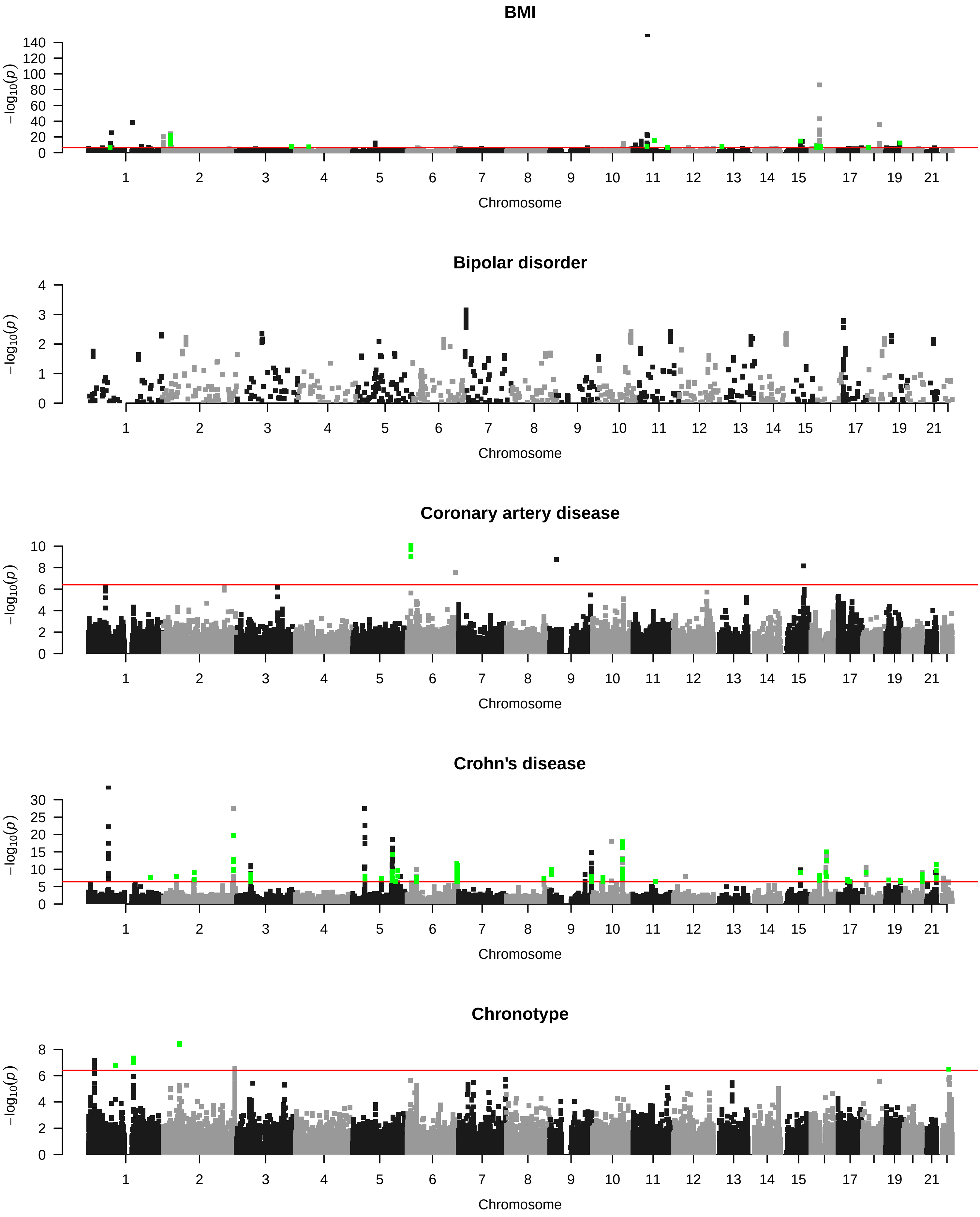
BMI
140 120 100 80 60 40 20 0
−log10(p)
1 2 3 4 5 6 7 8 9 10 11 12 13 14 15 17 19 21
Chromosome
Bipolar disorder
4 3 2 1 0
−log10(p)
1 2 3 4 5 6 7 8 9 10 11 12 13 14 15 17 19 21
Chromosome
Coronary artery disease
10 8 6 4 2 0
−log10(p)
1 2 3 4 5 6 7 8 9 10 11 12 13 14 15 17 19 21
Chromosome
Crohn's disease
30 25 20 15 10 5 0
−log10(p)
1 2 3 4 5 6 7 8 9 10 11 12 13 14 15 17 19 21
Chromosome
Chronotype
8 6 4 2 0
−log10(p)
1 2 3 4 5 6 7 8 9 10 11 12 13 14 15 17 19 21
Chromosome

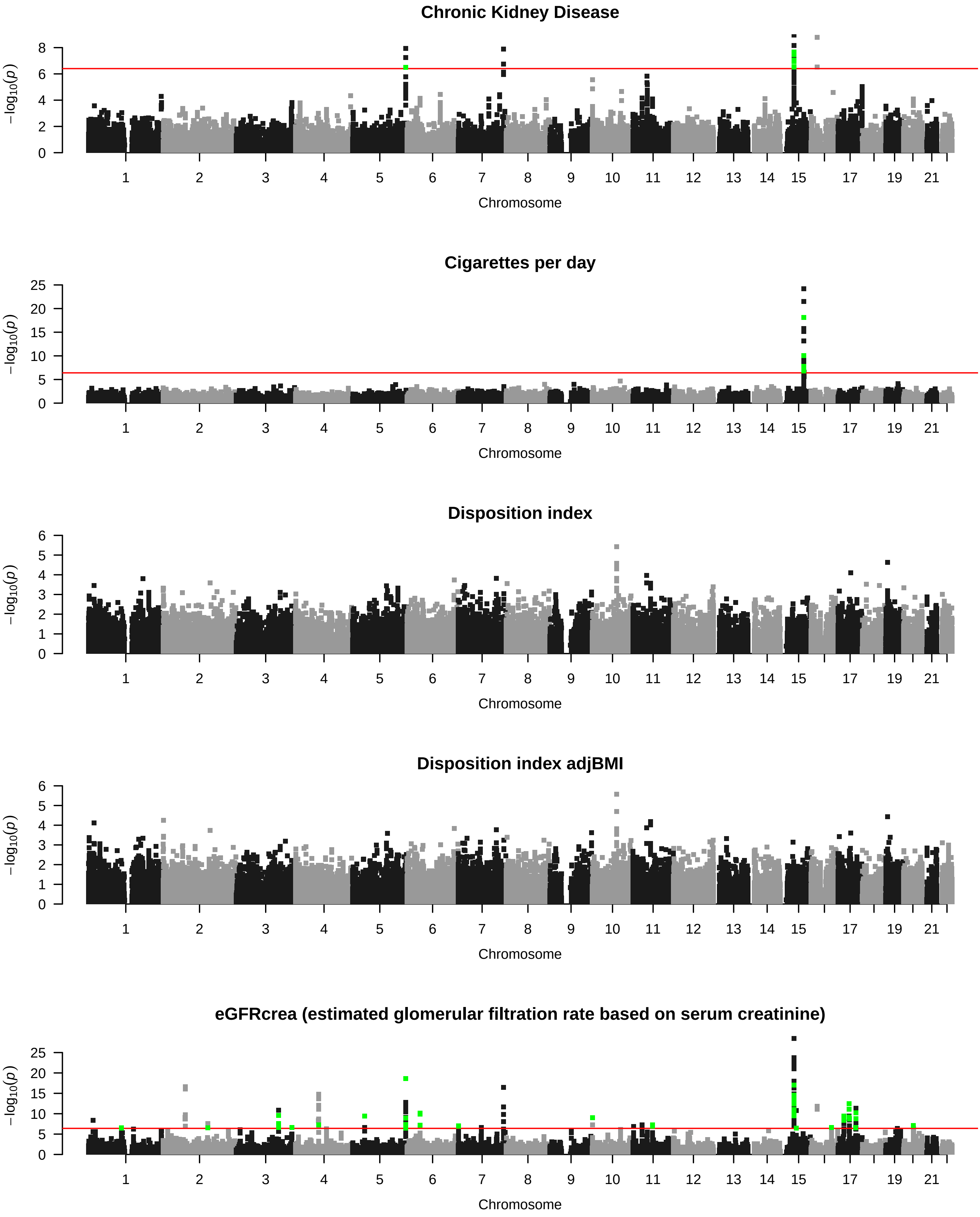
Chronic Kidney Disease
Cigarettes per day
Disposition index
Disposition index adjBMI
eGFRcrea (estimated glomerular filtration rate based on serum creatinine)
$-\log_{10}(p)$
Chromosome
1
2
3
4
5
6
7
8
9
10
11
12
13
14
15
17
19
21

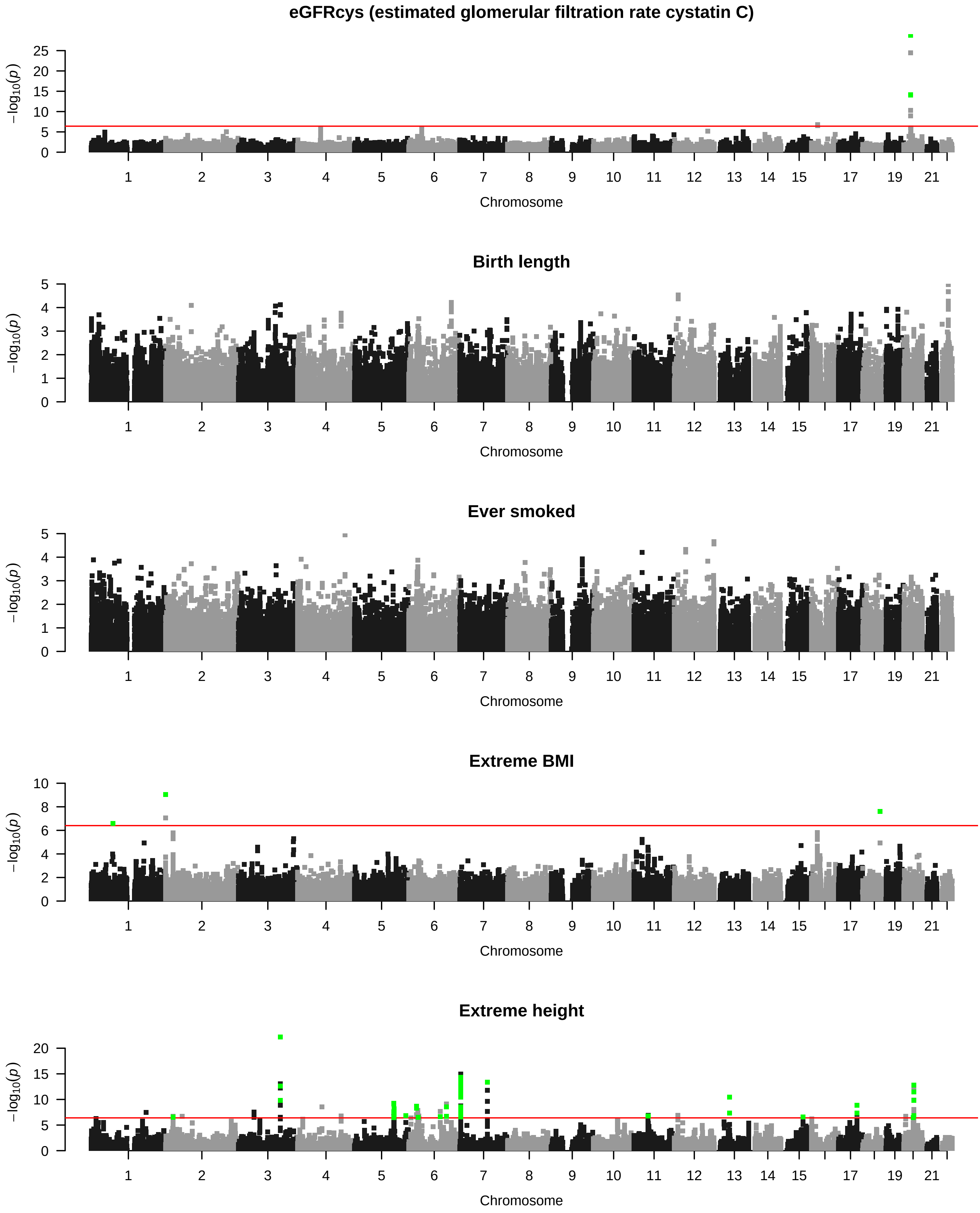
eGFRcys (estimated glomerular filtration rate cystatin C)
Birth length
Ever smoked
Extreme BMI
Extreme height
$-\log_{10}(p)$
Chromosome
1
2
3
4
5
6
7
8
9
10
11
12
13
14
15
17
19
21

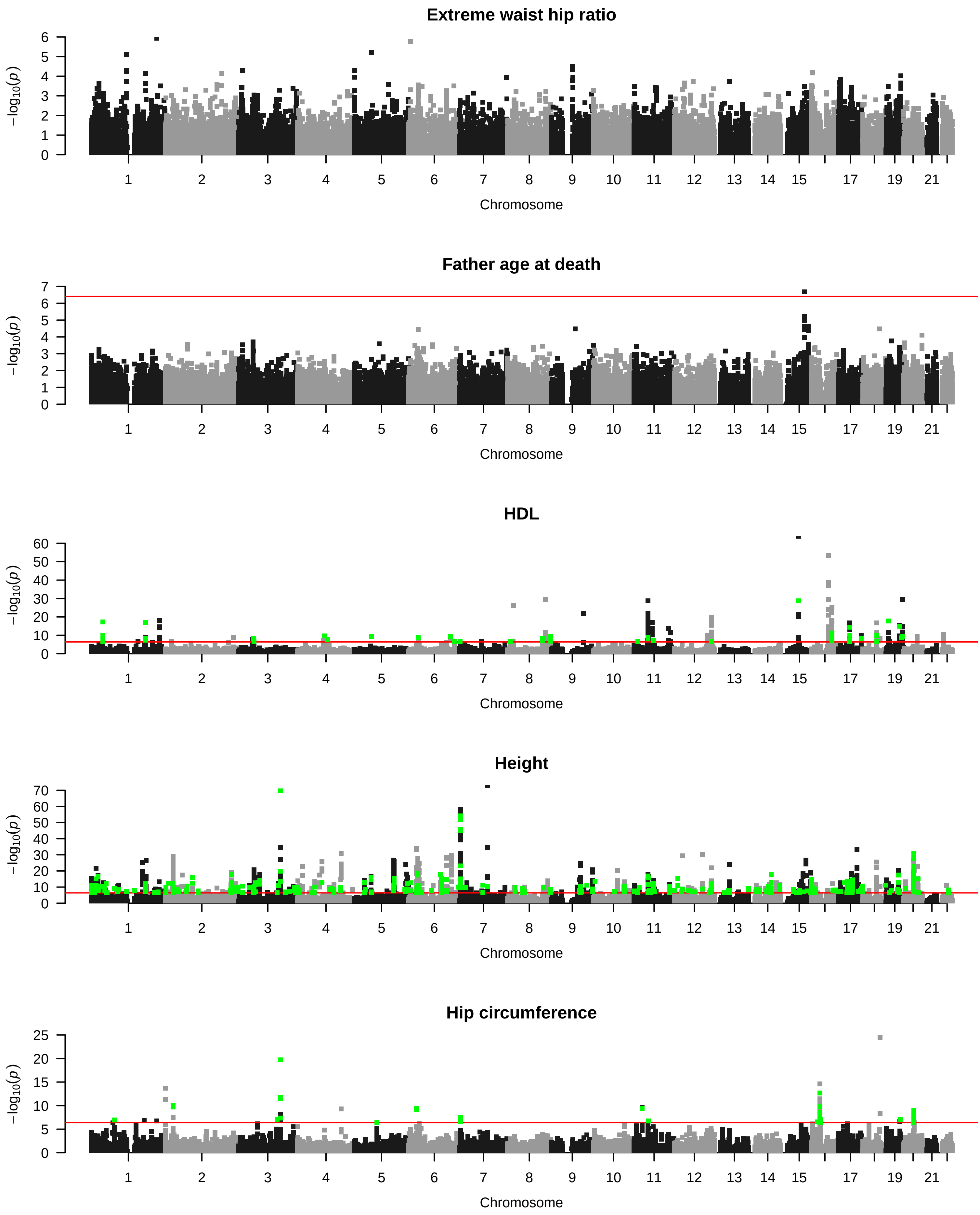
Extreme waist hip ratio
Father age at death
HDL
Height
Hip circumference
−log10(p)
Chromosome
1
2
3
4
5
6
7
8
9
10
11
12
13
14
15
17
19
21

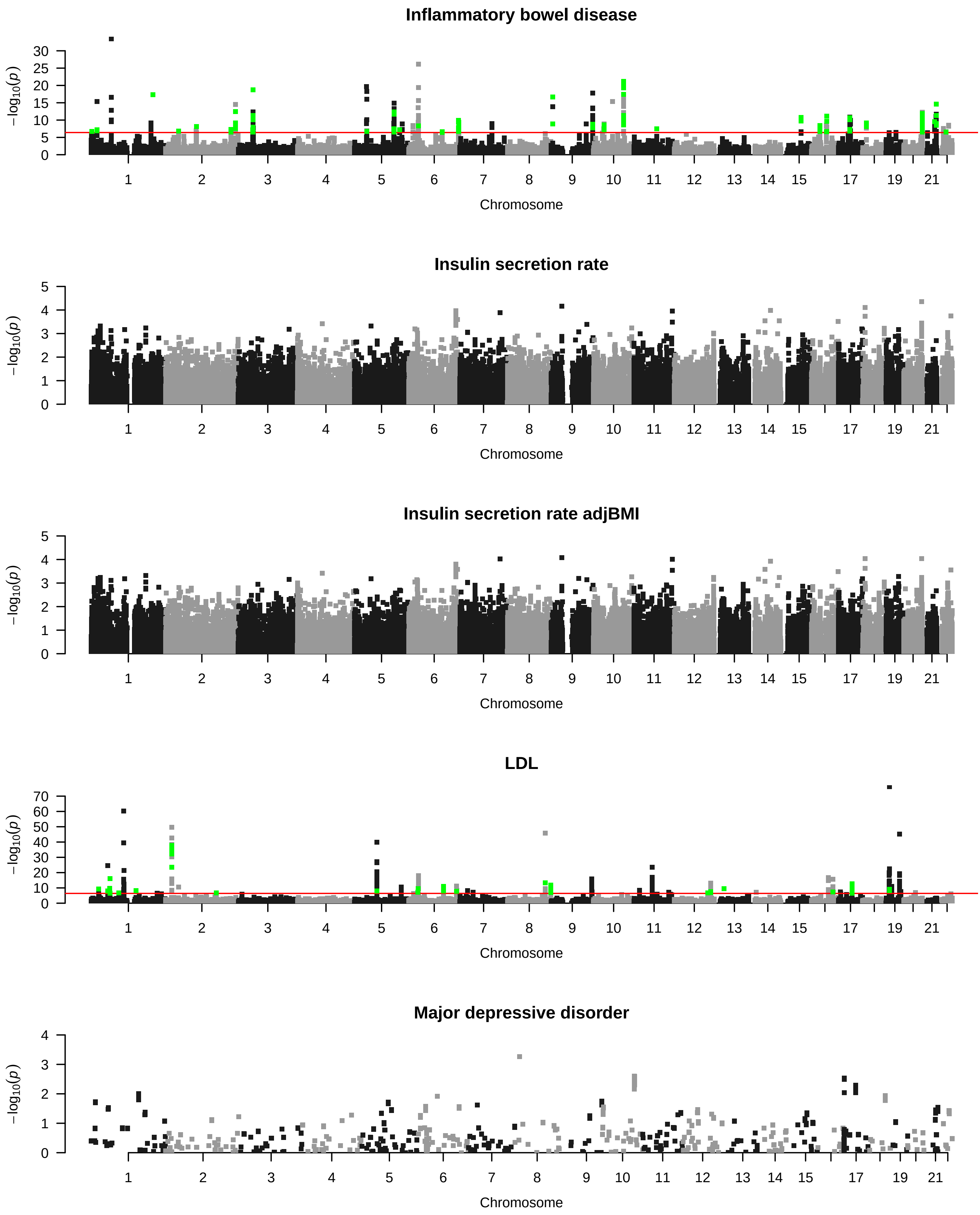

Inflammatory bowel disease
Insulin secretion rate
Insulin secretion rate adjBMI
LDL
Major depressive disorder
$-\log_{10}(p)$
Chromosome
1
2
3
4
5
6
7
8
9
10
11
12
13
14
15
17
19
21

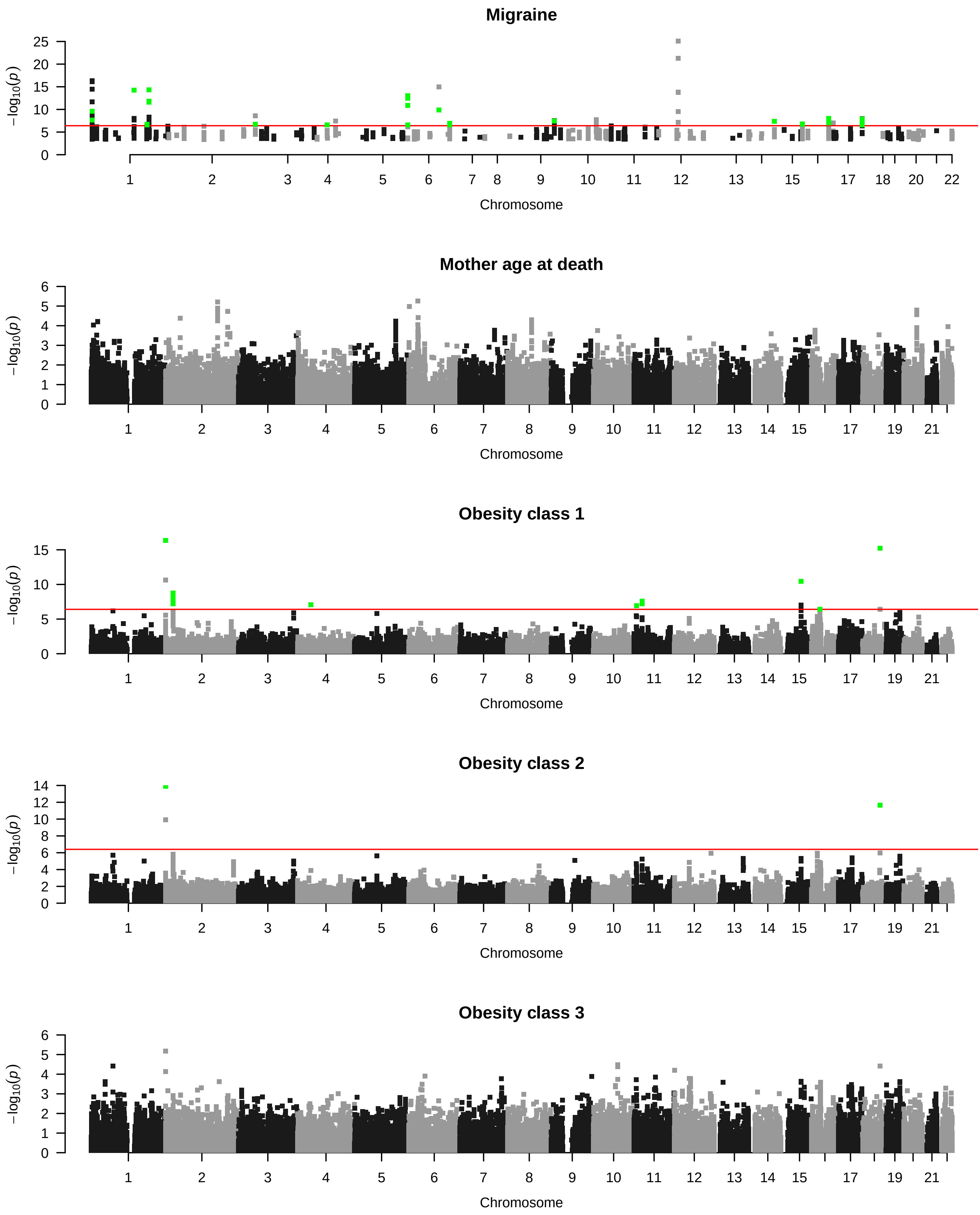
Migraine
−log10(p)
Chromosome
Mother age at death
−log10(p)
Chromosome
Obesity class 1
−log10(p)
Chromosome
Obesity class 2
−log10(p)
Chromosome
Obesity class 3
−log10(p)
Chromosome

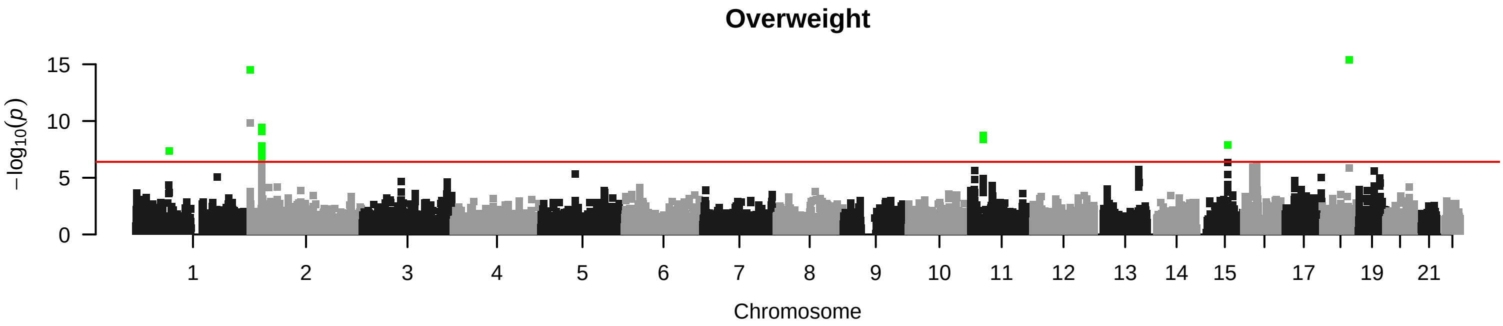
Overweight
15
10
5
0
−log10(p)
1 2 3 4 5 6 7 8 9 10 11 12 13 14 15 17 19 21
Chromosome

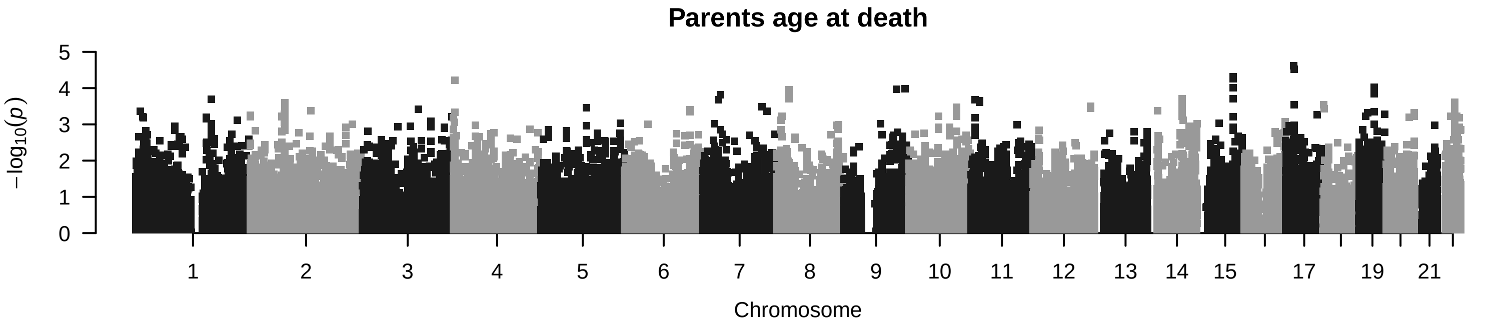
Parents age at death
5
4
3
2
1
0
−log10(p)
1 2 3 4 5 6 7 8 9 10 11 12 13 14 15 17 19 21
Chromosome

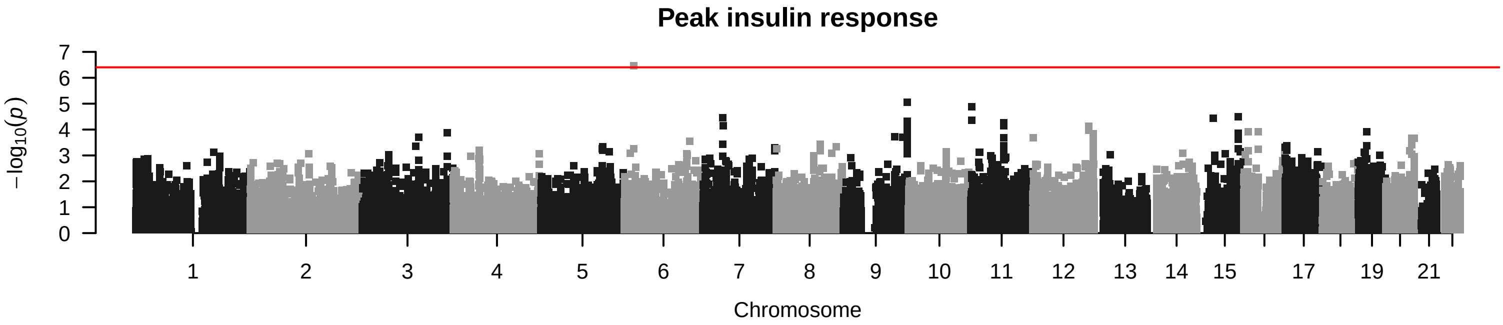
Peak insulin response
7
6
5
4
3
2
1
0
−log10(p)
1 2 3 4 5 6 7 8 9 10 11 12 13 14 15 17 19 21
Chromosome

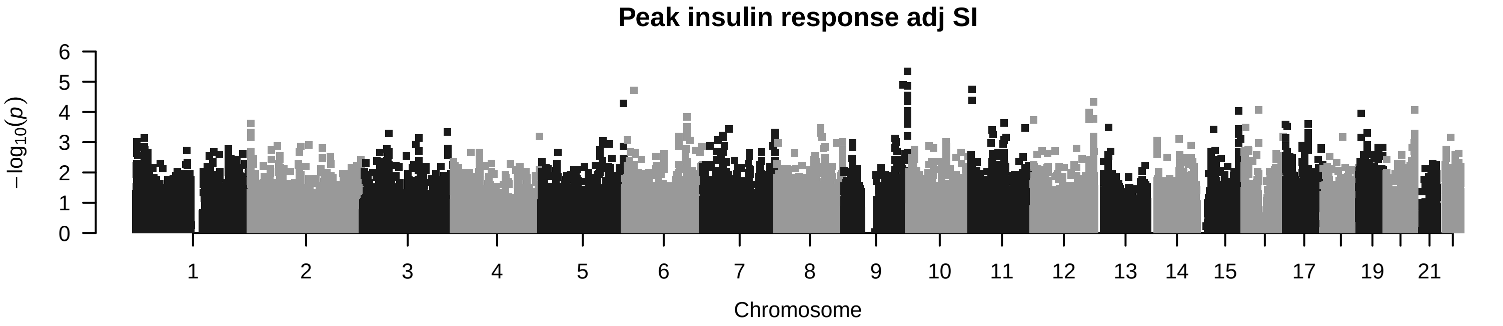
Peak insulin response adj SI
6
5
4
3
2
1
0
−log10(p)
1 2 3 4 5 6 7 8 9 10 11 12 13 14 15 17 19 21
Chromosome

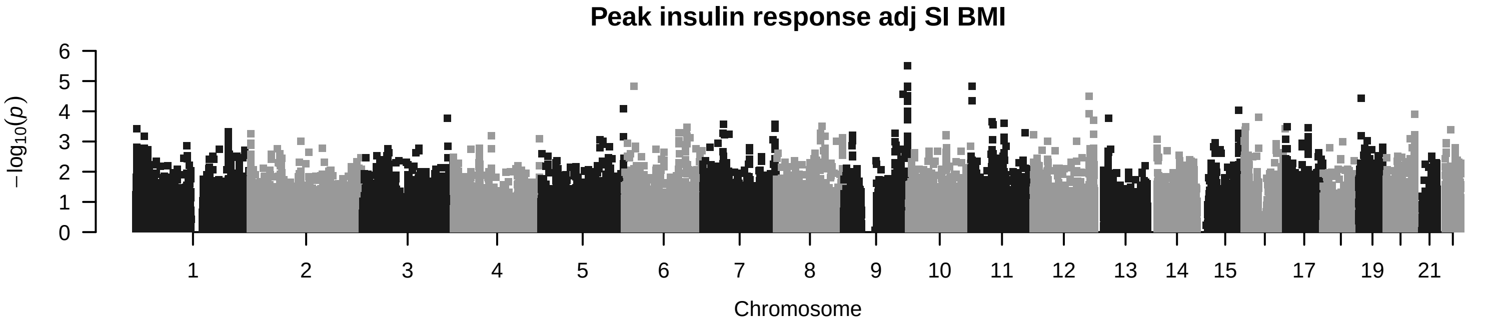
Peak insulin response adj SI BMI
6
5
4
3
2
1
0
−log10(p)
1 2 3 4 5 6 7 8 9 10 11 12 13 14 15 17 19 21
Chromosome

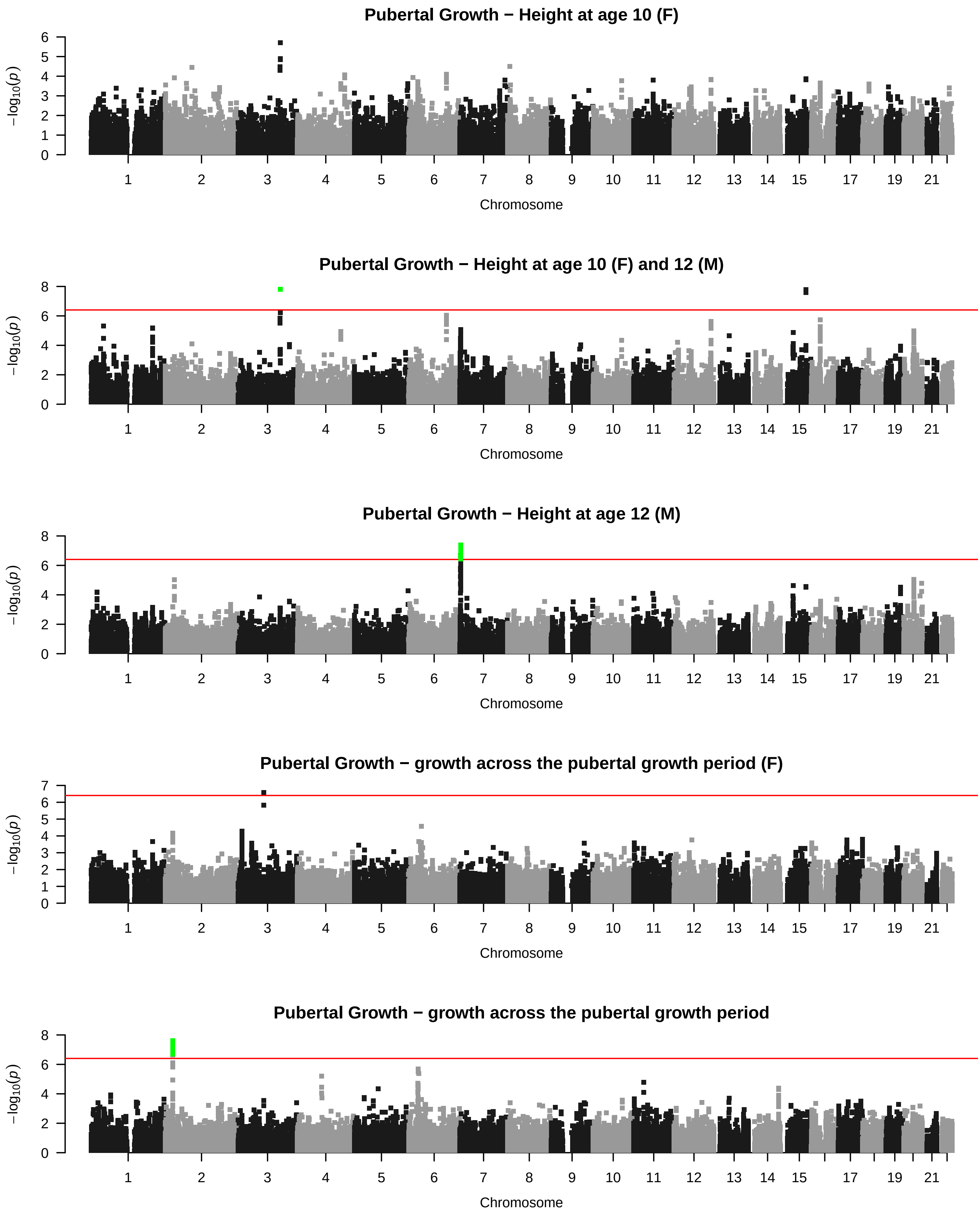
Pubertal Growth − Height at age 10 (F)
Pubertal Growth − Height at age 10 (F) and 12 (M)
Pubertal Growth − Height at age 12 (M)
Pubertal Growth − growth across the pubertal growth period (F)
Pubertal Growth − growth across the pubertal growth period
−log10(p)
Chromosome
1 2 3 4 5 6 7 8 9 10 11 12 13 14 15 17 19 21

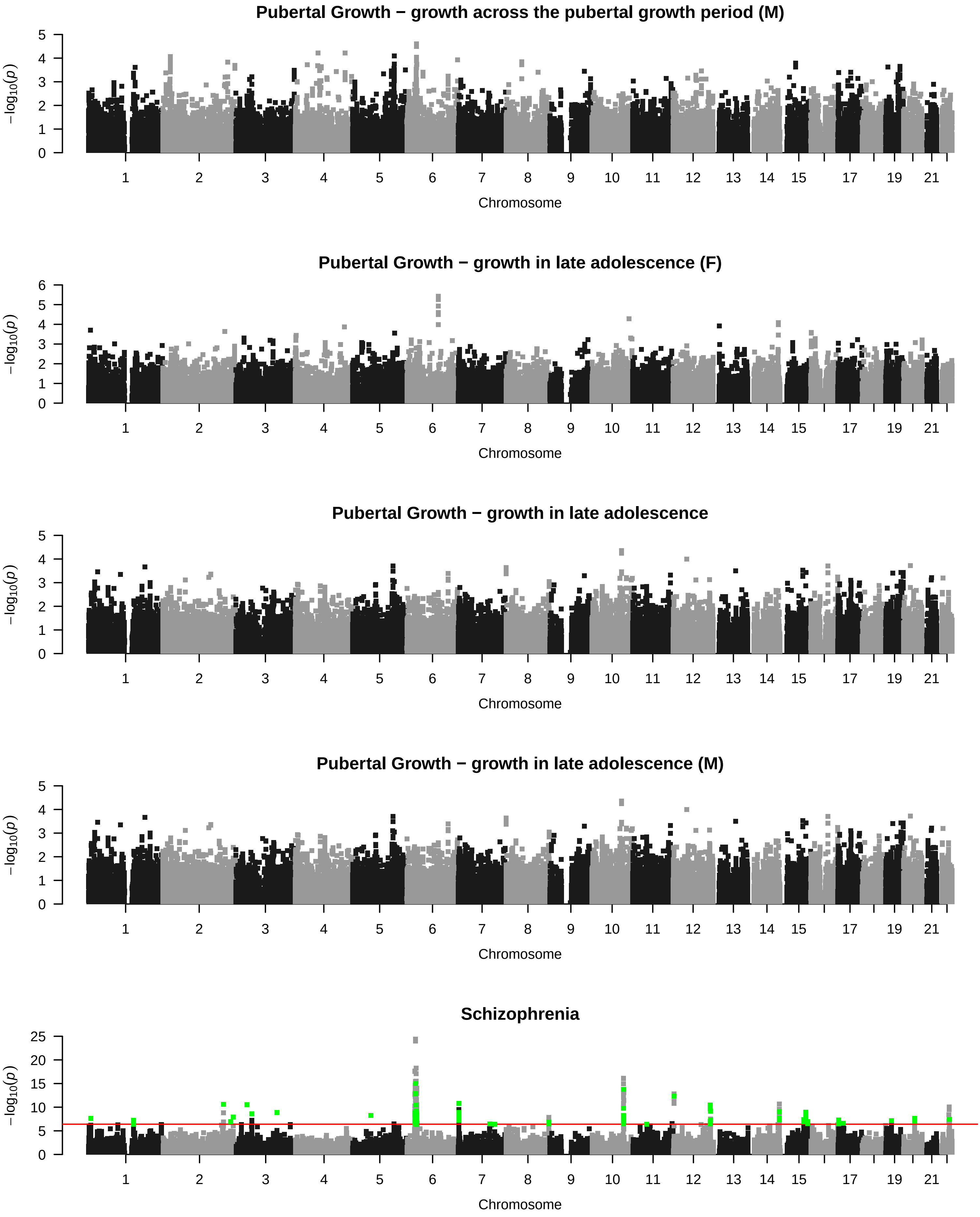
Pubertal Growth − growth across the pubertal growth period (M)
Pubertal Growth − growth in late adolescence (F)
Pubertal Growth − growth in late adolescence
Pubertal Growth − growth in late adolescence (M)
Schizophrenia
$-\log_{10}(p)$
Chromosome
1 2 3 4 5 6 7 8 9 10 11 12 13 14 15 17 19 21

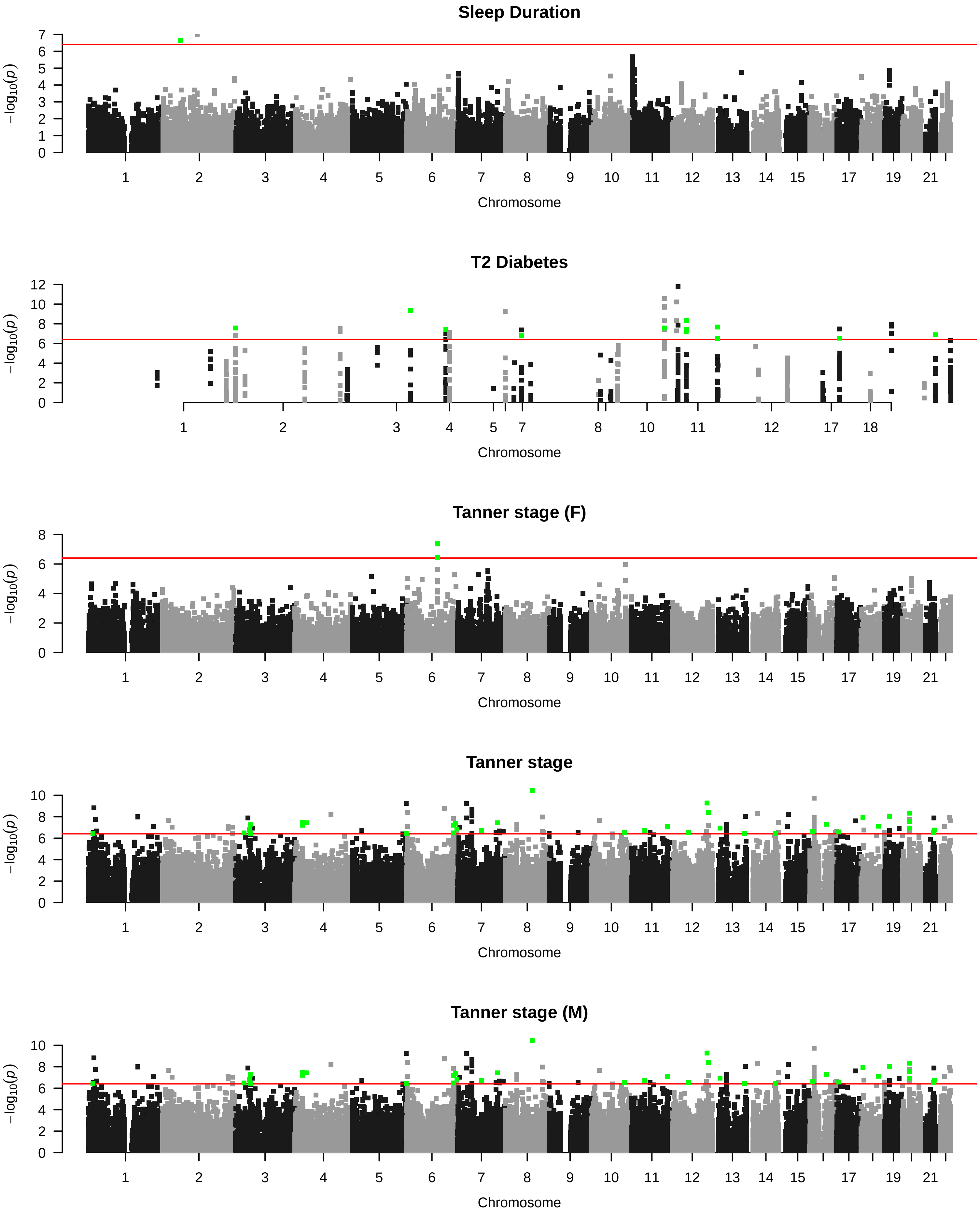
Sleep Duration
−log10(p)
0 1 2 3 4 5 6 7
1 2 3 4 5 6 7 8 9 10 11 12 13 14 15 17 19 21
Chromosome
T2 Diabetes
−log10(p)
0 2 4 6 8 10 12
1 2 3 4 5 7 8 10 11 12 17 18
Chromosome
Tanner stage (F)
−log10(p)
0 2 4 6 8
1 2 3 4 5 6 7 8 9 10 11 12 13 14 15 17 19 21
Chromosome
Tanner stage
−log10(p)
0 2 4 6 8 10
1 2 3 4 5 6 7 8 9 10 11 12 13 14 15 17 19 21
Chromosome
Tanner stage (M)
−log10(p)
0 2 4 6 8 10
1 2 3 4 5 6 7 8 9 10 11 12 13 14 15 17 19 21
Chromosome

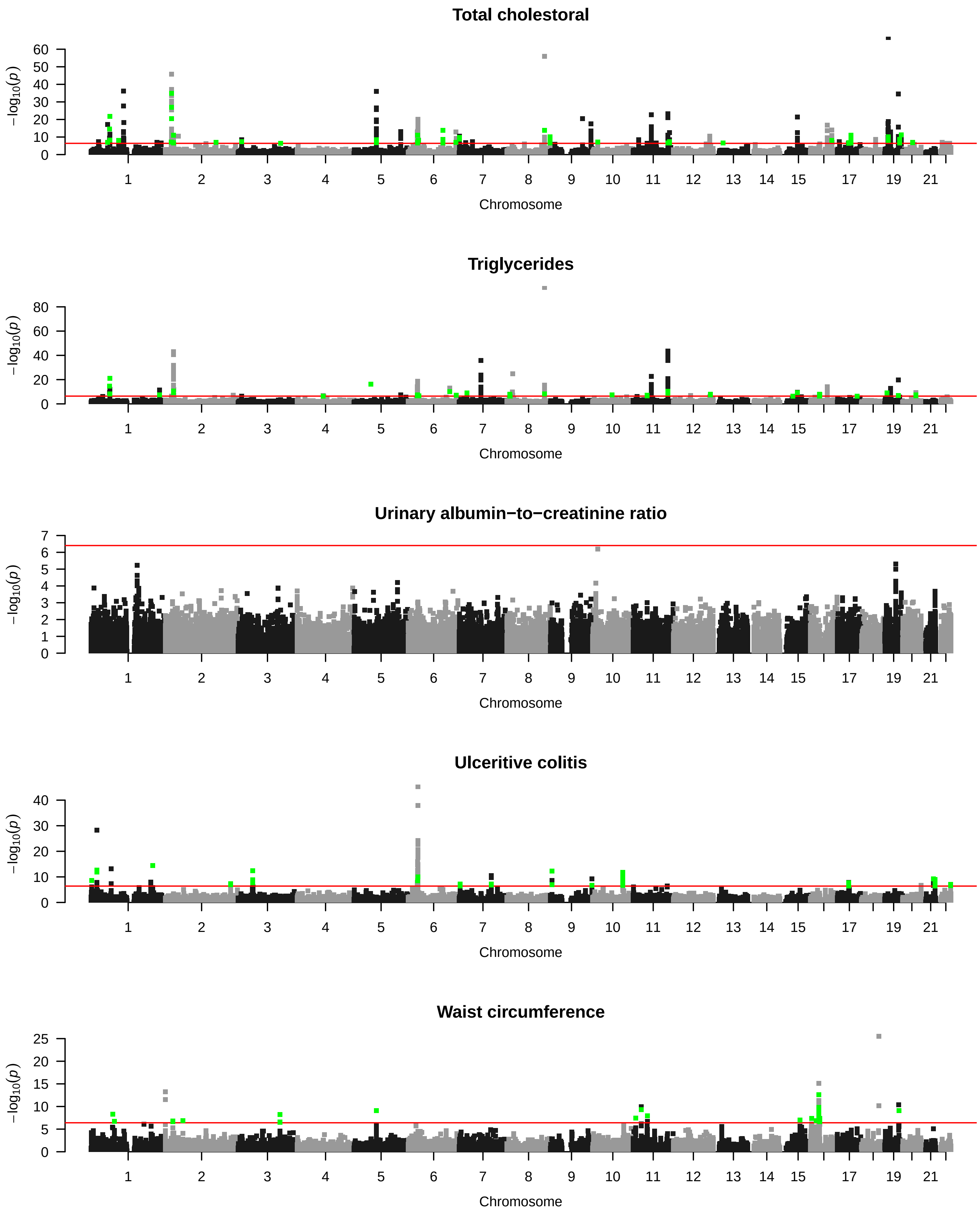

Total cholestoral
Triglycerides
Urinary albumin−to−creatinine ratio
Ulceritive colitis
Waist circumference
−log10(p)
Chromosome
1 2 3 4 5 6 7 8 9 10 11 12 13 14 15 17 19 21

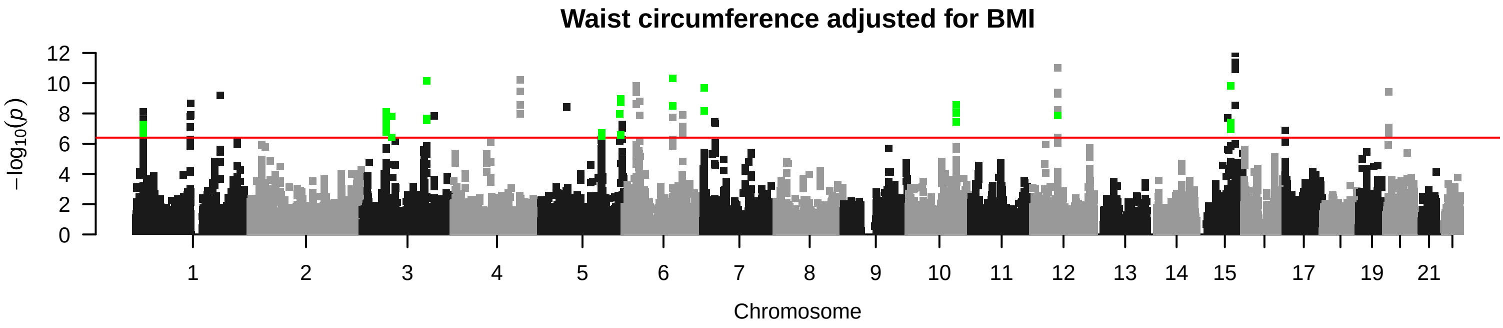
Waist circumference adjusted for BMI
−log10(p)
0 2 4 6 8 10 12
1 2 3 4 5 6 7 8 9 10 11 12 13 14 15 17 19 21
Chromosome

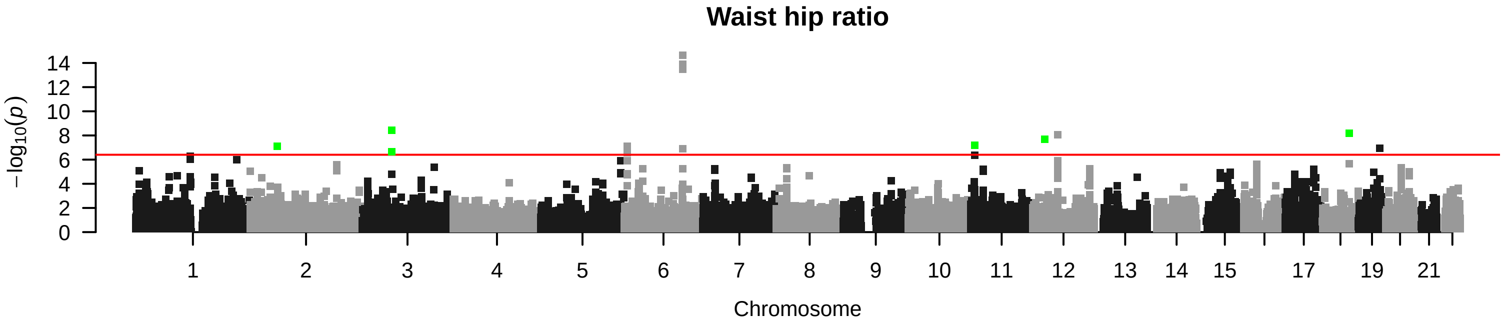
Waist hip ratio
−log10(p)
0 2 4 6 8 10 12 14
1 2 3 4 5 6 7 8 9 10 11 12 13 14 15 17 19 21
Chromosome

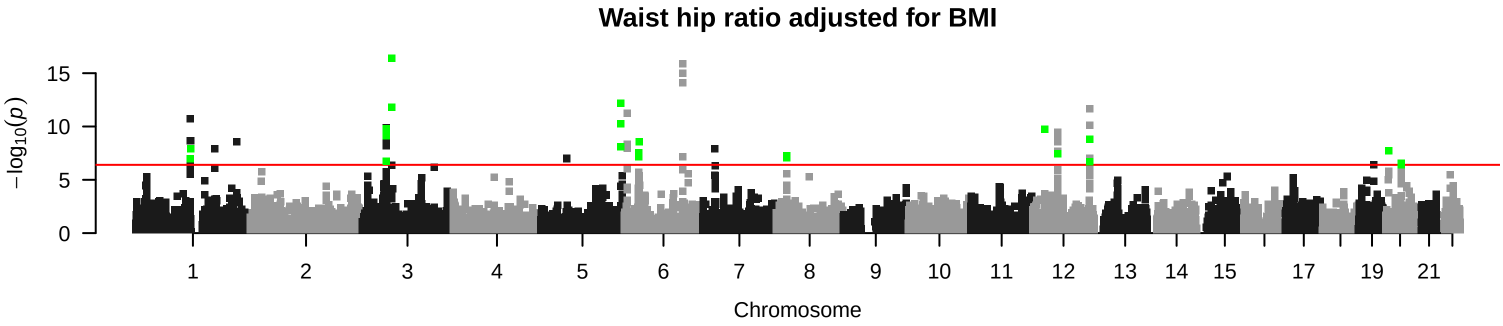
Waist hip ratio adjusted for BMI
−log10(p)
0 5 10 15
1 2 3 4 5 6 7 8 9 10 11 12 13 14 15 17 19 21
Chromosome

Figure S14: Replication of significant pleiotropic associations across two independent datasets. Scatterplots of estimated causal effect from Summary data-based Mendelian Randomization (SMR) analysis between the UKHLS (x-axis) and our previous study (y-axis)(Hannon et al. 2017). Each point represents a significant pleiotropic associations identified in UKHLS also tested in the replication dataset. Green points indicate associations significant in both datasets.

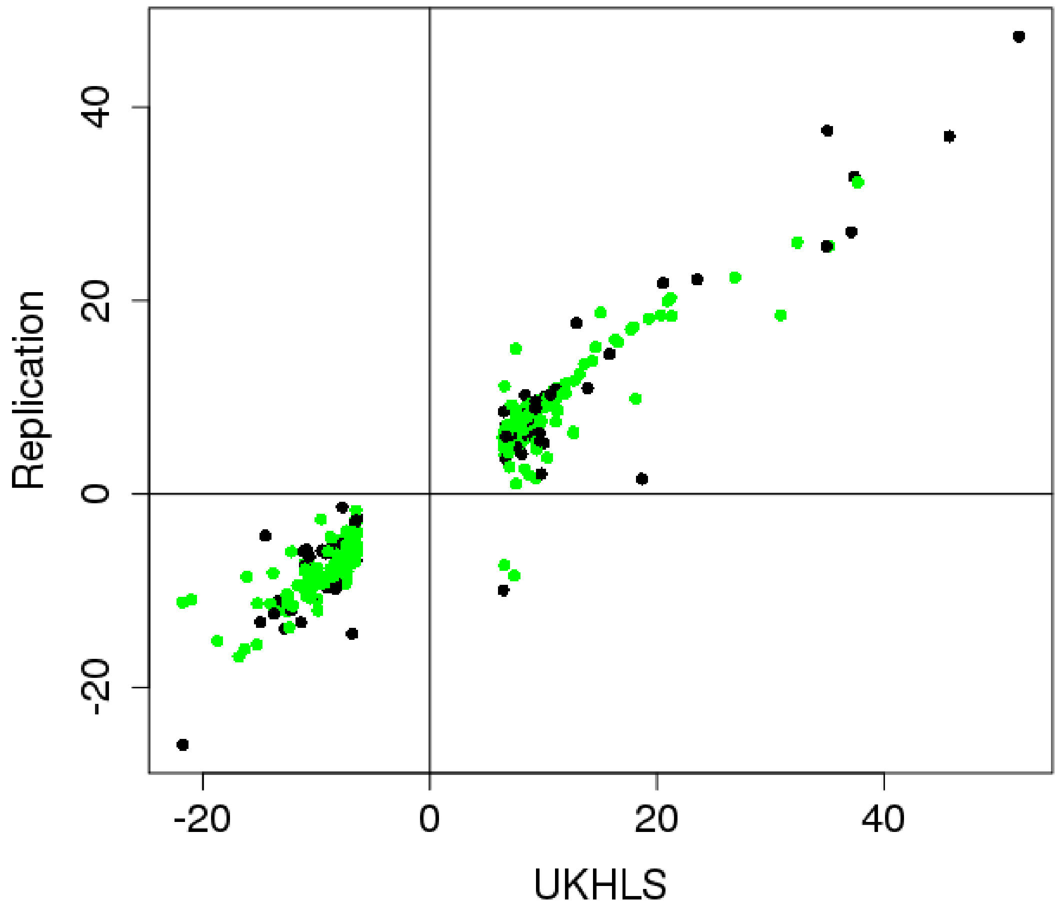

Figure S15: Flowchart showing the number of gene expression probes, DNAm sites and pairs significant at each stage of the analysis.

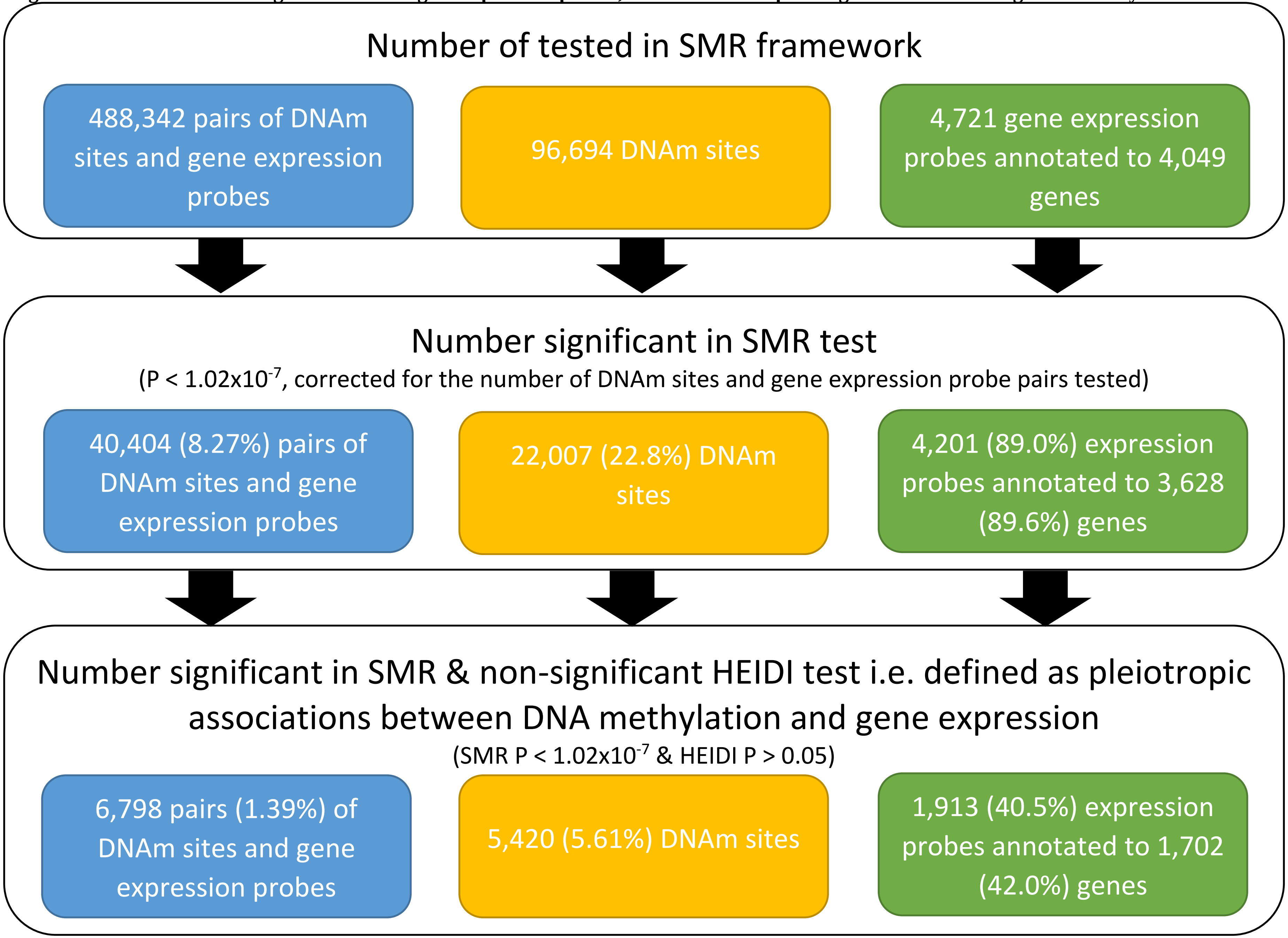

Figure S16: Genomic distribution of DNA methylation sites pleiotropically associated with gene expression. Barplot demonstrating the genic location of DNA methylation sites pleiotropically associated with gene expression (blue bars) compared to the background rate inferred from all DNA methylation sites included in this analysis (gray bars). There was enrichment for DNA methylation sites to be located in the gene body and promotor (TSS1500, TSS200, 5’UTR) and depleted in intergenic regions, see **Table S9** for frequency statistics.

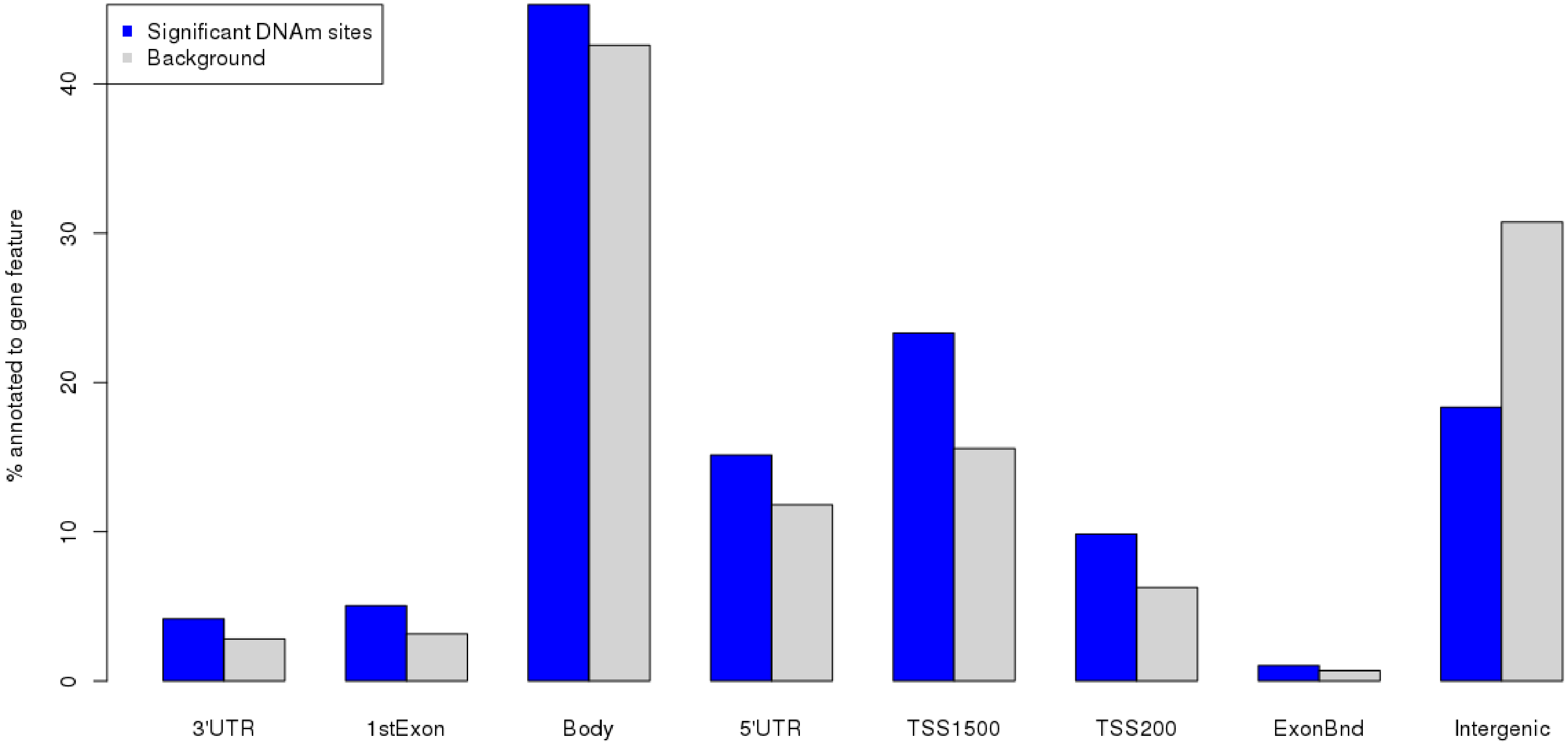

Figure S17: Distribution of estimated effect of pleiotropic associations between DNA methylation and gene expression, stratified by genic location of DNA methylation site. Violin plots of effect size estimated from the SMR analysis between DNA methylation and gene expression. Each violin plot represents pleiotropic associations where the DNA methylation site is annotated to that genic feature. All features are associated with both positive and negative relationships with gene expression, however there is a significant shift towards negative associations for 1st Exon (P = 6.19e-05) 5'UTR (P = 0.00108), TSS200 (P = 6.38e-07), TSS1500 (P = 5.82e-11), gene body (P = 0.0230).

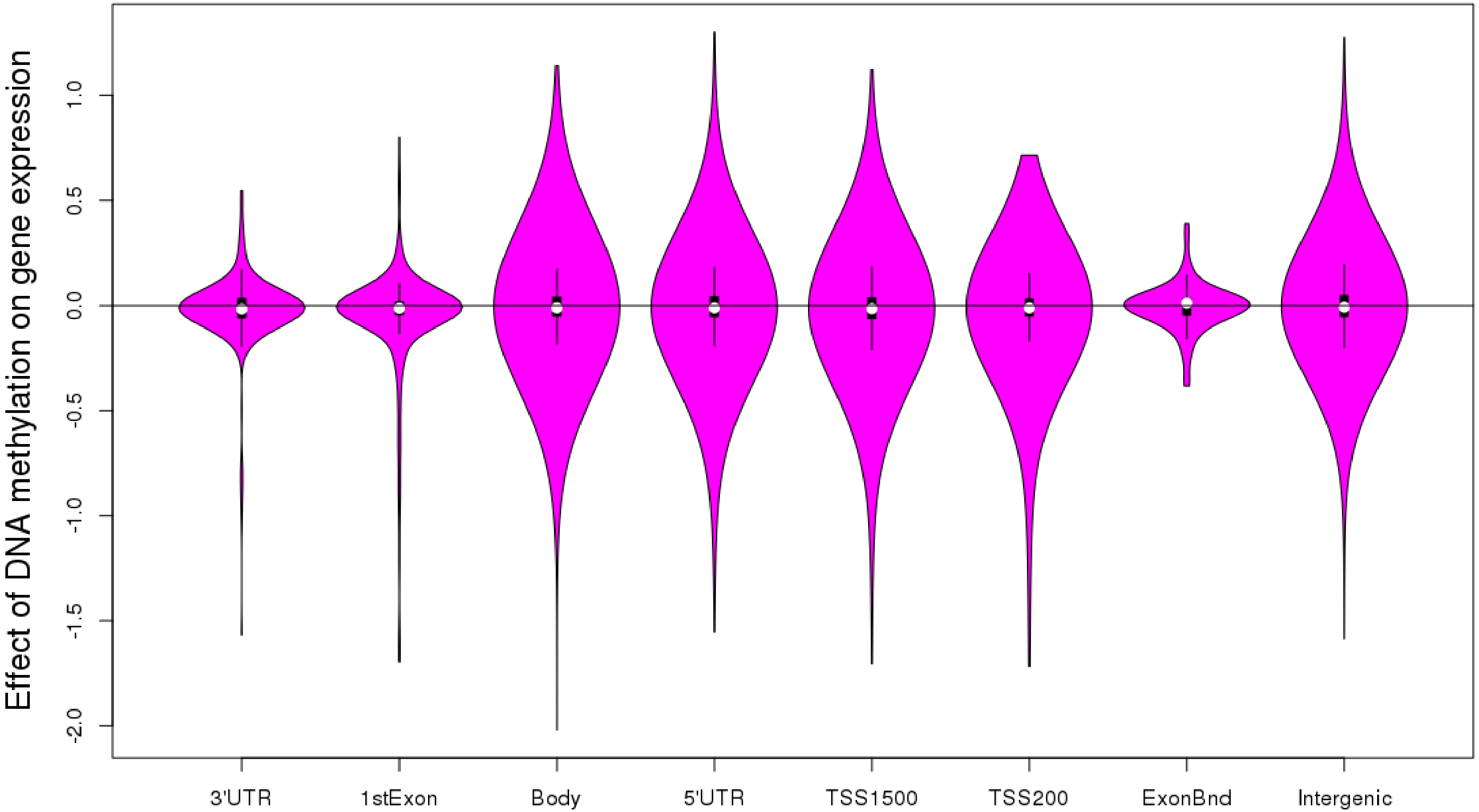

Table S1: Summary of DNA methylation quantitative trait loci analysis in Understanding Society (n = 1,111). A) all mQTL; B) mQTL classed as cis (distance between genetic variant and DNA methylation site < 500kb); C) mQTL classed as trans.

A)

P value threshold	nMQTL	nSNPs	nProbes	DNA methylation change per allele	
				mean	SD
6.52E-14	12689548	2907234	93268	3.46%	3.01%
1.00E-13	12886785	2926784	94209	3.44%	3.00%
1.00E-12	14040864	3036038	99833	3.31%	2.93%
1.00E-11	15399232	3152092	106408	3.18%	2.85%
1.00E-10	17051673	3281391	114595	3.03%	2.76%

B)

P value threshold	nMQTL	nSNPs	nProbes	DNA methylation change per allele	
				mean	SD
6.52E-14	11679376	2837748	89325	3.48%	3.03%
1.00E-13	11854125	2857053	90197	3.46%	3.02%
1.00E-12	12866269	2964911	95439	3.33%	2.95%
1.00E-11	14055426	3079629	101414	3.20%	2.87%
1.00E-10	15479136	3204842	108405	3.05%	2.79%

C)

P value threshold	nMQTL	nSNPs	nProbes	DNA methylation change per allele	
				mean	SD
6.52E-14	1010172	398965	7791	3.26%	2.78%
1.00E-13	1032660	404570	7948	3.23%	2.76%
1.00E-12	1174595	438405	8935	3.10%	2.66%
1.00E-11	1343806	478896	10224	2.98%	2.56%
1.00E-10	1572537	531470	12347	2.83%	2.45%

Table S2: Summary of additional associations from novel content on Illumina EPIC BeadArray compared to Illumina 450K BeadArray.

P value threshold	DNAm probes associated with an mQTL				Genes associated with an mQTL			Intergenic DNA methylation sites associated with an mQTL	
	All	EPIC specific			All	Only associated with EPIC specific	Additional association with EPIC of 450K association	All	EPIC specific
		Total	within 1kb of 450k association	within 5kb of 450k association					
6.52E-14	93268	48099	8509	15627	16276	5172	6521	31087	17780
1.00E-13	94209	48593	8613	15809	16360	5185	6577	31408	17955
1.00E-12	99833	51407	9180	17020	16802	5211	6905	33310	19010
1.00E-11	106408	54715	9931	18513	17266	5235	7277	35399	20185
1.00E-10	114595	58884	10929	20451	17848	5268	7711	38063	21677

Table S3: Frequency of DNAm sites in A) genic feature and B) CpG Island feature annotation categories.

A)

Genic annotation category	DNAm sites associated with mQTL (P < 6.52x10-14)		All tested DNAm sites	
	Number	%	Number	%
**Body**	38495	41.27353433	330180	42.11656868
**TSS200**	5805	6.223999657	75322	9.607802369
**TSS1500**	13567	14.54625381	116805	14.89922407
**5'UTR**	10478	11.23429258	103362	13.18448353
**3'UTR**	2349	2.518548698	22363	2.852543538
**ExonBnd**	524	0.561821847	6857	0.874654163
**1stExon**	2963	3.176866664	43993	5.611588243
**Intergenic**	31087	33.33083158	217106	27.6932575

B)

CpG island annotation category	DNAm sites associated with mQTL (P < 6.52x10-14)		All tested DNAm sites	
	Number	%	Number	%
**Island**	10878	11.66316421	149471	19.06598109
**Shore**	19850	21.28275507	141724	18.07780174
**Shelf**	6278	6.73114037	54623	6.967512663
**Sea**	56262	60.32294034	438149	55.8887045

Table S9: Frequency of DNAm sites associated with gene expression in gene feature annotation categories.

Genic annotation category	DNAm sites associated with gene expression (SMR P < 1.02x10-7 & HEIDI P > 0.05)		All DNAm sites tested against gene expression	
	Number	%	Number	%
3'UTR	226	4.169741697	2728	2.821271227
1stExon	274	5.055350554	3044	3.148075372
Body	2456	45.31365314	41186	42.59416303
5'UTR	820	15.12915129	11425	11.81562455
TSS1500	1263	23.30258303	15063	15.57800898
TSS200	533	9.833948339	6050	6.256851511
ExonBnd	56	1.033210332	668	0.690839142
Intergenic	994	18.33948339	29727	30.74337601

References

Hannon E, Weedon M, Bray N, O'Donovan M, Mill J. 2017. Pleiotropic Effects of Trait-Associated Genetic Variation on DNA Methylation: Utility for Refining GWAS Loci. *Am J Hum Genet* doi:10.1016/j.ajhg.2017.04.013.

van Dongen J, Nivard MG, Willemsen G, Hottenga JJ, Helmer Q, Dolan CV, Ehli EA, Davies GE, van Iterson M, Breeze CE et al. 2016. Genetic and environmental influences interact with age and sex in shaping the human methylome. *Nat Commun* **7**: 11115.
