# Supplementary material for: Leveraging DNA-Methylation Quantitative-Trait Loci to Characterize the Relationship between Methylomic Variation, Gene Expression, and Complex Traits
Source: Am J Hum Genet. 2018 Oct 25;103(5):654–65. doi: 10.1016/j.ajhg.2018.09.007 (PMC6217758; doi:10.1016/j.ajhg.2018.09.007)
Supplement: Document S3. Article plus Supplemental Data [file mmc3.pdf]

ARTICLE

Leveraging DNA-Methylation Quantitative-Trait Loci to Characterize the Relationship between Methylomic Variation, Gene Expression, and Complex Traits

Eilis Hannon,[1] Tyler J. Gorrie-Stone,[2] Melissa C. Smart,[3] Joe Burrage,[1] Amanda Hughes,[3] Yanchun Bao,[3] Meena Kumari,[3] Leonard C. Schalkwyk,[2] and Jonathan Mill[1,*]

Characterizing the complex relationship between genetic, epigenetic, and transcriptomic variation has the potential to increase understanding about the mechanisms underpinning health and disease phenotypes. We undertook a comprehensive analysis of common genetic variation on DNA methylation (DNAm) by using the Illumina EPIC array to profile samples from the UK Household Longitudinal study. We identified 12,689,548 significant DNA methylation quantitative trait loci (mQTL) associations ($p < 6.52 \times 10^{-14}$) occurring between 2,907,234 genetic variants and 93,268 DNAm sites, including a large number not identified by previous DNAm-profiling methods. We demonstrate the utility of these data for interpreting the functional consequences of common genetic variation associated with > 60 human traits by using summary-data-based Mendelian randomization (SMR) to identify 1,662 pleiotropic associations between 36 complex traits and 1,246 DNAm sites. We also use SMR to characterize the relationship between DNAm and gene expression and thereby identify 6,798 pleiotropic associations between 5,420 DNAm sites and the transcription of 1,702 genes. Our mQTL database and SMR results are available via a searchable online database as a resource to the research community.

Introduction

DNA methylation (DNAm), an epigenetic modification to cytosine, is involved in mediating the developmental regulation of gene expression and function, as well as transcriptional processes such as genomic imprinting and X chromosome inactivation.[1,2] Although often regarded as a mechanism of transcriptional repression, the relationship between DNAm and gene expression is highly complex and not fully understood.[3] Gene-body DNAm, for example, is often associated with active expression[4] and also influences other transcriptional processes, including alternative splicing and promoter usage.[5] This dynamic property of DNAm means it can vary across samples and might underlie phenotypic differences. There is growing interest in characterizing the variation of DNAm across populations[6,7] and in the role of DNAm in disease, and recent epigenome-wide association studies (EWASs) have identified robust associations between variable DNAm and cancer,[8] as well as a diverse range of other complex phenotypes, including rheumatoid arthritis [MIM: 180300],[9] body-mass index,[10] schizophrenia [MIM: 181500],[11] and Alzheimer disease [MIM: 104300].[12] Characterizing the complex relationship between genetic, epigenetic, and transcriptomic variation will increase understanding about the mechanisms underpinning health and disease phenotypes. Twin and family studies have demonstrated that population-level variation in DNAm is under considerable genetic control, although these effects vary across genomic loci, developmental stages, and different cell and tissue types.[13–17] Studies in a variety of tissues, including brain, whole blood, pancreatic islet cells, and adipose tissue, have identified widespread associations between common DNA sequence variants and DNAm.[17–22] These DNAm quantitative trait loci (mQTLs) are primarily *cis*-acting, are enriched in regulatory chromatin domains and transcription-factor binding sites, and have been shown to colocalize with gene expression quantitative trait loci (eQTLs).[3,17,23]

There is considerable interest in using mQTLs, along with other types of molecular QTLs, to interpret the functional consequences of common genetic variation associated with human traits, especially because the actual gene(s) involved in mediating phenotypic variation are not necessarily the most proximal to the lead SNPs identified in genome-wide association studies (GWASs). Of note, GWAS variants are enriched in enhancers and regions of open chromatin,[24,25] reinforcing the hypothesis that most common genetic risk factors influence gene regulation rather than directly affecting the coding sequences of transcribed proteins.[26] Importantly, evidence for the co-localization of genetic variants associated with both phenotypic and regulatory variation is not sufficient to show that the overlapping association signals are causally related; additional analytical steps are needed to distinguish pleiotropic effects—i.e., where the same variant is influencing both outcomes, although not necessarily dependently—from those that are an artifact of linkage disequilibrium (LD). We recently extended the use of one approach—summary-data-based Mendelian randomization (SMR), which was initially used in conjunction with expression quantitative trait loci (eQTL) data[27]—to prioritize genes for GWAS-nominated loci using mQTL data.[28]

[1]University of Exeter Medical School, University of Exeter, Exeter EX2 5DW, United Kingdom; [2]School of Biological Sciences, University of Essex, Colchester, CO4 3SQ, United Kingdom; [3]Institute for Social and Economic Research, University of Essex, Colchester CO3 3LG, United Kingdom
*Correspondence: j.mill@exeter.ac.uk
https://doi.org/10.1016/j.ajhg.2018.09.007.
© 2018 The Authors. This is an open access article under the CC BY license (http://creativecommons.org/licenses/by/4.0/).

Building on our previous work, we used the Illumina EPIC array and imputed SNP data to identify mQTLs associated with variable DNAm at ~850,000 sites across the genome in samples from the Understanding Society UK Household Longitudinal Study (UKHLS) (n = 1,111). We then used these mQTLs within the SMR framework to refine genetic association data from publicly available GWAS datasets in order to prioritize genes involved in 63 complex traits and diseases. We subsequently used the SMR approach to identify pleiotropic relationships between DNAm and variable gene expression by using publicly available whole-blood gene eQTL data. Our mQTL database and SMR results are available via a searchable online database as a resource to the research community (see Web Resources).

Subjects and Methods

Sample Description

The British Household Panel Survey (BHPS) began in 1991, and in 2010 it was incorporated into the larger UKHLS[29] (also known as Understanding Society), which is a longitudinal panel survey of 40,000 UK households from England, Scotland, Wales, and Northern Ireland. Since 1991, annual interviews have collected sociodemographic information, and in 2011–2012, biomedical measures and blood samples for BHPS participants were collected during a nurse visit in the participant's home. Respondents were eligible to give a blood sample if they had taken part in the previous main interview in English; were 16 or older; lived in England, Wales, or Scotland; were not pregnant; and met other conditions detailed in the user guide.[30] For each participant, non-fasting blood samples were collected through venipuncture; these were subsequently centrifuged so that plasma and serum were separated, and samples were aliquoted and frozen at −80°C. DNA has been extracted and stored for genetic and epigenetic analyses.

Genome-wide Quantification of DNAm

DNAm was profiled in DNA extracted from whole blood for 1,193 individuals who were aged from 28 to 98; who were eligible for and consented to both blood sampling and genetic analysis; who had been present at all annual interviews between 1999 and 2011; and whose time between blood sample collection and processing did not exceed 3 days. Eligibility requirements for genetic analyses meant that the epigenetic sample was restricted to participants of white ethnicity. The EZ-96 DNA Methylation-Gold kit (Zymo Research) was used for treating 500 ng of DNA from each sample with sodium bisulfite. DNAm was quantified with the Illumina Infinium HumanMethylationEPIC BeadChip run on an Illumina iScan System according to the manufacturer's standard protocol. Samples were randomly assigned to chips and plates so that batch effects would be minimized. In addition, the inclusion of a fully methylated control (CpG Methylated HeLa Genomic DNA; New England BioLabs) in a random position on each plate facilitated sample tracking and helped to resolve experimental inconsistencies and confirm data quality.

DNAm Data Preprocessing

Raw signal intensities were imported from.idat files into the R statistical environment[31] and converted into beta values (the proportion of DNA methylation at individual sites was measured) with the *bigmelon* package.[32] These data were processed via a standard pipeline including the following steps: (1) detection of outlier samples via principal-component analysis and Mahalanobis distance equivalents, (2) confirmation of complete bisulphite conversion via control probes, (3) comparison of estimated age from the data via the Horvath Epigenetic Clock algorithm[33] and reported age at sampling, and (4) visualization of principal components. Data were normalized with the *dasen* function within the *wateRmelon* package,[34] which performs background adjustment and between-sample quantile normalization of methylated (M) and unmethylated (U) intensities separately for type I and type II probes. Samples that were dramatically altered as a result of normalization were excluded on the basis of the difference between the normalized and raw data; those with a root mean square and standard deviation > 0.05 were removed. Samples were then filtered so that those with >1% of sites with a detection p value > 0.05 were excluded. Finally, DNA-methylation sites with a bead count <3 were excluded along with those in which >1% of the sample had a detection p value > 0.05. The raw DNA methylation data from the final sample set was then re-normalzsed with the *dasen* function. The final dataset included 857,071 DNA-methylation sites and 1,175 individuals for subsequent analysis. These DNAm data are available upon request through the European Genome-Phenome Archive under accession code EGAS00001001232.

Annotation of DNAm Sites

The genomic location of DNAm sites along with genic, DNase hypersensitivy sites and open chromatin annotation were taken from the manifest files provided by Illumina and downloaded from the product support pages (see Web Resources).

Genotyping and Imputation

UKHLS samples were genotyped with the Illumina Infinium HumanCoreExome BeadChip Kit as previously described (12v1-0).[35] This array contains a set of >250,000 highly informative genome-wide tagging single-nucleotide polymorphisms (SNPs) as well as a panel of functional (protein-altering) exonic markers, including a large proportion of low-frequency (MAF 1%–5%) and rare (MAF < 1%) variants. Genotype calling was performed with the *gencall* algorithm within GenomeStudio (Illumina). After only the samples with matched DNAm data were selected, variants were refiltered prior to imputation. PLINK[36] was used for removing samples with >5% missing data. We also excluded SNPs characterized by >5% missing values, a Hardy-Weinberg equilibrium p value < 0.001, and a minor-allele frequency of <5%. For identification of related samples, SNPs underwent LD pruning, and the *–genome* command in PLINK was used for calculating the proportion of identity-by-descent for all pairs of samples; 58 pairs of related samples (PI_HAT > 0.2) were identified, and randomly excluding one individual from each pair ensured that the samples were independent. These data were then imputed with the 1000 Genomes phase 3 version5 reference panels SHAPEIT and minimac3.[37] Best-guess genotypes were called, and variants were filtered to those with a minor-allele frequency >0.01 and an INFO score >0.8. Because variants were named using their locations ("chr:pos") and variant type (SNP/INDEL), duplicate variants were also excluded. Principal components were calculated from the imputed genotype data via GCTA (a tool for genome-wide complex-trait analysis).[38] 16 samples

were identified as being outliers (defined as more than 2 standard deviations from the mean) in a scatterplot of the first two principal components and were excluded from subsequent genetic analyses. Principal components were then recalculated for inclusion as covariates in QTL analyses. The imputed genetic variants were then filtered so that variants characterized by >5% missing values, a Hardy-Weinberg equilibrium p value <0.001, a minor-allele frequency of <5%, and a minimum of five observations in each genotype group were excluded. These genotype data are available on application through the European Genome-phenome Archive under accession code EGAS00001001232.

DNAm Quantitative-Trait Loci

Cross-hybridizing probes, probes with a common SNP (European population minor-allele frequency > 0.01) within 10 bp of the CpG site or a single base extension[39,40] and probes on the sex chromosomes were excluded from the QTL analysis. In addition, 977 substandard probes identified by Illumina were also excluded. We performed a genome-wide mQTL analysis; in total, we tested 766,714 DNAm sites against 5,210,475 genetic variants by using the R package *MatrixEQTL*.[41] This package enables fast computation of QTLs by only saving those more significant than a predefined threshold (set to $p = 1 \times 10^{-8}$ for this analysis). We fitted an additive linear model to test whether the number of alleles (coded 0,1,2) predicted DNAm at each site; we included covariates for age, sex, six estimated cellular composition variables (B cells, CD8 T cells, CD4 T cells, monocytes, granulocytes, natural killer T cells),[42,43] two binary batch variables, and the first ten principal components from the genotype data to control for ethnicity differences. We used a Bonferroni-corrected multiple-testing threshold, set to genome-wide significance for GWAS and divided by the number of DNAm sites tested (i.e., $5 \times 10^{-8}/766714 = 6.52 \times 10^{-14}$). We used the *clump* command in *PLINK*[36] to identify the number of independent associations for each DNAm site with more than 1 significant mQT by using the following parameters: *–clump-p1 1e-8–clump-p2 1e-8–clump-r2 0.1–clump-kb 250*.

Bayesian Co-localization

Out of all DNAm sites with at least 1 significant mQTL ($p < 1 \times 10^{-10}$), all pairs of DNAm sites located on the same chromosome and within 250 kb of each other were tested for co-localization. Because data for all SNPs (regardless of significance) are required for this analysis, first, the mQTL analysis was rerun for these DNAm sites so that all association statistics (p value, regression coefficient, and t-statistic, so that the standard error could be inferred) could be recorded for all SNPs within 500 kb of the DNAm site. Co-localization analysis was performed as previously described[44] with the R *coloc* package (see Web Resources). From our mQTL results we input the regression coefficients, their variances, and SNP minor-allele frequencies, and we left the prior probabilities as their default values. This methodology allowed us to quantify the support across the results of each GWAS for five hypotheses by calculating the posterior probabilities, denoted as *PPi* for hypothesis *Hi*.

H_0: there exist no causal variants for either CpG site;
H_1: there exists a causal variant for CpG_1 only;
H_2: there exists a causal variant for CpG_2 only;
H_3: there exist two distinct causal variants, one for each CpG; or
H_4: there exists a single causal variant common to both CpGs.

Summarized Mendelian Randomization Analysis 1: Identifying Putative Pleiotropic Relationships between DNAm and Complex Traits

SMR analysis between DNAm and complex traits was performed with publicly available software (see Web Resources) as previously described.[27,28] Publicly available genome-wide association study (GWAS) results were downloaded from a range of sources and converted to the appropriate format for the SMR analysis. We renamed SNPs in the 1000 Genomes format (chr:bp) to align them with the mQTL output by using dbSNP version 141 (where SNP locations for hg19 were not provided in the results file). Where allele frequency was not provided, it was taken from the European subset of 1000 Genomes (phase 3, version 5). Details for how each set of results was processed can be found in Table S4. We used significant mQTLs ($p < 1 \times 10^{-10}$) calculated in the UKHLS sample to identify genetic instruments for 126,457 DNAm sites that were included in the SMR analysis. The SMR test comprises of two steps. First, we performed a two-sample Mendelian randomization with the two-step least-squares (2SLS) approach by using the effect size of the top cis-QTL SNP and its corresponding effect in the GWAS. The significance threshold for this part of test was set at 3.95×10^{-7}, calculated by the Bonferroni correction method and adjusted for the number of DNAm sites tested (0.05/126,457). Second, we tested for heterogeneity of effects by using alternative SNPs as the instrumental variable, on the basis of the theory that if both DNAm and the GWAS trait were associated with the same causal variant, the choice of SNP would be irrelevant, whereas if they were associated with different causal variants, the differing linkage disequilibrium relationships between the instruments and each causal variant would lead to variation in the estimated effect between the trait and DNAm. Non-significant heterogeneity (heterogeneity in dependent instruments [HEIDI] $p > 0.05$) indicates that there is a pleiotropic effect on a GWAS trait and DNAm. This approach was repeated with publicly available eQTL data from Westra et al.;[45] in this analysis, significant pleotropic associations between gene expression and complex traits were selected as those with SMR p $< 8.38 \times 10^{-6}$ (corrected for 5,966 gene expression probes tested) and HEIDI $p > 0.05$.

Summarized Mendelian Randomization Analysis 2: Identifying Putative Pleiotropic Relationships between DNAm and Gene Expression

We used a second application of the SMR analysis to identify pleiotropic relationships between DNAm and gene expression. Gene eQTL results from the Westra eQTL study[45] were downloaded along with the SMR software. SNP IDs were converted to the 1000 Genomes format (so they would match the mQTL output), and SNP frequencies were taken from the European subset of 1000 Genomes (phase 3, version 5). These data included eQTLs at 5,966 probes. All pairs of CpG and genes where tested as long as (1) the CpG had a significant mQTL ($p < 1 \times 10^{-10}$), (2) the gene had a significant eQTL ($p < 5 \times 10^{-8}$), and (3) there was a common genetic variant tested within 500 kilobases of the gene expression probe and DNAm site. In total, 488,342 pairs of DNAm sites and gene expression transcripts were tested; therefore, the significance threshold for the first stage of the SMR test was set to $p < 1.02 \times 10^{-7}$ after a Bonferroni correction for the number of tests was applied. Consistent with all other SMR analyses in this manuscript, a non-significant heterogeneity test (HEIDI $p > 0.05$) in step 2 of the SMR analysis was used for classifying pleiotropic relationships from artifacts of linkage disequilibrium.

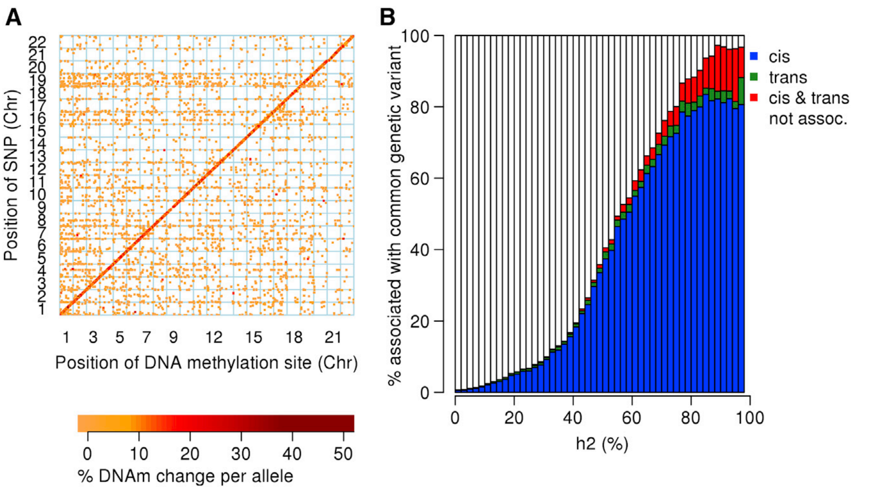

Figure 1. DNA-Methylation Quantitative-Trait Loci Are Predominantly *cis*-Acting and Enriched in Sites at Which DNAm Is Highly Heritable
(A) The genomic distribution of Bonferroni-significant ($p = 6.52 \times 10^{-14}$) mQTLs in whole blood; the position on the x axis indicates the location of Illumina EPIC array probes, and the position on the y axis indicates the location of genetic variants. The color of the point corresponds to the difference in DNA methylation per allele compared to the reference allele; the largest effects are plotted in dark red. A clear positive diagonal can be observed, demonstrating that the majority of mQTLs are associated with genotype in *cis*.
(B) A bar plot of the percentage of DNA-methylation sites associated with common genetic variation and grouped by previous reported estimates of heritability (percent variation in DNAm is explained by additive genetic factors taken from van Dongen et al.[13]). Each bar plot demonstrates the percentage of DNA-methylation sites with Bonferroni significant genetic effects in *cis* only (blue), *trans* only (green), and both *cis* and *trans* (red) and with no significant genetic effects (white).

Enrichment Analyses

DNAm sites were annotated to genes and CpG islands with the information provided in the Illumina manifest file, which is based on the UCSC RefGene and CpG island databases. Sites are annotated to genes if they are located within the gene body or up to 1,500 base pairs form the transcription start site. Sites are annotated to CpG islands if they are located within the boundaries of a CpG island, to a shore if they are located up to 2,000 base pairs from an island, or to a shelf if they are between 2,000 and 4,000 base pairs from an island. Frequency tables were used for recording the number of sites annotated to each feature category, and Chi-square tests were used for identifying different distributions across these annotation categories between all tested DNAm sites and the subset of sites considered for enrichment analysis (e.g., all DNAm sites with at least one significant mQTL).

Data Availability

Summary statistics for all Bonferroni-significant DNA-methylation quantitative-trait loci are available for download from the Complex Disease Epigenomics Group website, where readers can also explore many of the results included in this manuscript through our interactive web application. Analysis scripts used in this manuscript are available on GitHub, and data on phenotypes linked to DNA methylation are available on METADAC. See the Web Resources and the Accession Numbers sections.

Results

Additional mQTL Associations Identified with the Illumina EPIC Array

An overview of our study design is presented in Figure S1. We tested 5,210,475 imputed genetic variants against the 766,714 DNAm sites that were on the Illumina EPIC array and that passed our stringent QC criteria (see Subjects and Methods). We identified 12,689,548 significant mQTL associations (we used a conservative Bonferroni-corrected threshold of $p < 6.52 \times 10^{-14}$) between 2,907,234 genetic variants and 93,268 DNAm sites (Table S1; Figure 1A); there was a mean percentage point change in DNAm per additional reference allele of 3.46% (SD = 3.01%) across all mQTL-associated sites. Existing mQTL databases have been almost exclusively generated with the Illumina 450K array; more than half of the DNAm sites (n = 48,099, 51.6%; Table S2) that we identify as being associated with genetic variation with the Illumina EPIC array involve additional content not previously interrogated (Figure S2). Importantly, these additional mQTL associations are annotated to 5,172 genes not included in mQTL databases generated with the Illumina 450K array (Figure S3). DNAm sites associated with genetic variation are associated with a median of 65 (interquartile range = 22–162) mQTLs, probably reflecting linkage disequilibrium (LD) relationships between proximal variants. In contrast, each mQTL variant is associated with a median of two (interquartile range = 1–5) DNAm sites, and the majority of mQTL SNPs (n = 1,003,238, 34.5%) are associated with DNAm at only a single site (Figure S4). We performed LD clumping of the results for each DNAm site to identify the number of *independent* associations for each DNAm site (see Subjects and Methods); this process reduced the number of mQTL associations ($p < 6.52 \times 10^{-14}$) to 161,761 (1.27% of the total number of unclumped significant mQTL associations); a median of 1 (interquartile range = 1–2) mQTL variant associated with each DNAm site (Figure S5). At a more relaxed "discovery" threshold ($p < 1 \times 10^{-10}$), we identified a total of 17,051,673 mQTL associations between 3,281,391 genetic variants and 114,595 DNAm sites; these results are available in a searchable database (see Web Resources).

mQTL Associations Predominantly Occur in *cis* and Influence DNAm at Sites Known to Be Influenced by Heritable Factors

Consistent with the results of previous studies, we found that the majority of mQTL associations (n = 11,679,376;

92%) occur in *cis*, defined as situations where the distance between mQTL SNP and DNAm site is ≤500 kb[17,18,20,22] (Figure 1A). *Cis* mQTL variants are typically associated with larger effects on DNAm than those acting in *trans* (average *cis* effect = 3.48% change in DNAm per allele, average *trans* effect = 3.26% change in DNAm per allele; Mann-Whitney $p < 2.23 \times 10^{-308}$) (Figure S6). Furthermore, among *cis* mQTL associations, both significance and effect size increase as the distance between the genetic variant and DNAm site decreases (Figure S7). Compared to other tested DNAm sites, those associated with at least one mQTL variant (after correction for the number of tests performed [see Subjects and Methods], $p < 6.52 \times 10^{-14}$) are significantly enriched in intergenic regions and less likely to be located within both gene bodies (Chi square test: $p < 2.23 \times 10^{-308}$; Figure S8; Table S3A) and CpG islands (Chi square test $p < 2.23 \times 10^{-308}$; Figure S9; Table S3B). We used quantitative genetic data from a study of DNAm in monozygotic and dizygotic twins[13] to show that DNAm at sites associated with at least one mQTL variant is more strongly influenced by heritable (additive genetic) factors than are other tested DNAm sites (mQTL sites: median heritability, h^2, = 55% [interquartile range = 38%–71%]; all DNAm sites: median h^2 = 12% [interquartile range = 5%–31%]; Mann-Whitney $p < 2.23 \times 10^{-308}$; Figure S10). Overall, the proportion of sites at which DNAm is associated with an mQTL variant increases as a function of the estimated additive genetic influence derived from twin analyses (Figure 1B). Interestingly, there is no significant difference in the contribution of additive genetic effects to variance in DNAm at sites associated with *cis* (median h^2 = 56%; interquartile range = 39%–72%) and *trans* (median h^2 = 57%; interquartile range = 32%–76%) mQTL variants (Mann-Whitney p = 0.910).

Proximal DNA-Methylation Sites Share Genetic Associations

Similar to the LD relationships that exist between proximal genetic variants, DNAm levels are often correlated between proximally located DNAm sites.[14,46] To further characterize the genetic architecture of DNA methylation, we investigated whether shared genetic effects on multiple DNAm sites underlies this regional correlation structure. Although genetic variants are often associated with variation at multiple DNAm sites (Figure S4), this does not establish a shared genetic effect; shared genetic signals influencing a pair of DNAm sites might result from two distinct causal genetic variants that are in strong LD. To formally test whether neighboring DNAm sites are influenced by the same causal variant, we used a Bayesian co-localization approach[44] to interrogate all pairs of DNAm sites characterized as being located within 250 kb of each other and associated with at least one significant mQTL variant at our "discovery" significance threshold ($p < 1 \times 10^{-10}$). Our analyses assessed 3,535,812 pairs of DNAm sites with a median distance between DNAm sites of 110,493 bp (interquartile range = 47,914–178,085) and compared the pattern of mQTL associations for both DNAm sites to test whether they index an association with either the same causal variant or two distinct causal variants. We found that the posterior probabilities for virtually all of these (n = 3,520,781 [99.6%], median distance of 110,319 bp [interquartile range = 47,803–177,948]) supported a co-localized association within the same genomic region ($PP_3 + PP_4 > 0.99$). Of these, 281,898 pairs (8%) had sufficient support for the association of both DNAm sites with the same causal mQTL variant ($PP_3 + PP_4 > 0.99$ and $PP_4/PP_3 > 1$; Table S4); 234,460 pairs (6.6%) had "convincing" evidence ($PP_3 + PP_4 > 0.99$ and $PP_4/PP_3 > 5$) for co-localization of the same mQTL association according to the criteria of Guo and colleagues.[47] DNAm sites that shared genetic effects with at least one other DNAm site co-localize with a median of three other DNAm sites, indicating a complex relationship between genetic variation and DNAm in *cis*. Figure 2, for example, demonstrates that chromosome 9 contains a broad genomic region (>400 kb) where 38 DNAm sites—spanning seven genes—have a common underlying genetic signal. Of note, these DNAm sites are not contiguous; a small number of genetically mediated DNAm sites located within this region do not share the same mQTL signal. Pairs of DNAm sites with a shared causal mQTL variant are enriched for concordant directions of effect (71.2% pairs with positive correlations versus 28.8% pairs with negative correlations, binomial test $p = 1.48 \times 10^{-323}$; Figure S11). Furthermore, these pairs are located relatively close together (median distance between convincing co-localized pairs = 12,394 bp [interquartile range = 1,004–49,110]), with evidence that the shared genetic architecture is structured around annotated genomic features. Co-localized pairs of DNAm sites are significantly more likely to be annotated to the same gene (OR = 6.08, Fisher's test $p < 2.23 \times 10^{-308}$) or CpG island (OR = 1.54, Fisher's test $p < 2.23 \times 10^{-308}$) than non-co-localized pairs. Where pairs of DNAm sites with a shared genetic signal are annotated to the same gene, they are nominally less likely to be annotated to the same feature than are pairs of DNAm sites annotated to different genes (OR = 0.956, Fisher's test $p = 2.52 \times 10^{-7}$), suggesting that where genetic variation influences DNAm at multiple sites across a gene these sites do not necessarily cluster by genic feature and can be located anywhere from the transcription start site to the end of the last exon. DNAm is more likely to be positively correlated between pairs of co-localized sites annotated to the same gene than between pairs of sites annotated to different genes (OR = 1.85, Fisher's $p < 2.23 \times 10^{-308}$), a result driven predominantly by pairs of DNAm sites annotated to the same feature within that gene (OR = 1.57, Fisher's test $p = 3.41 \times 10^{-135}$) rather than those annotated to different features within a gene. Finally, pairs of DNAm sites with shared genetic effects annotated to the same genic feature, although not necessarily the same gene, are more likely to be positively correlated than pairs annotated to different

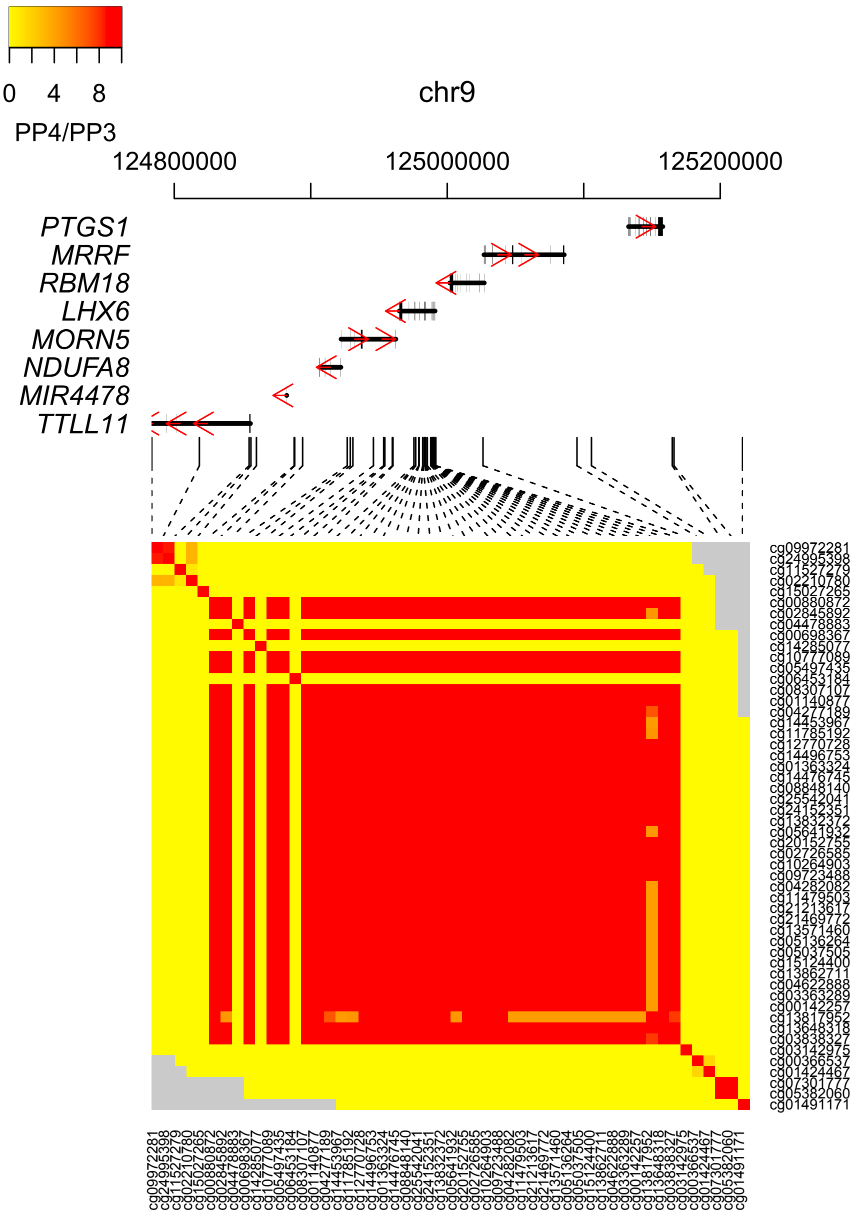

Figure 2. Shared Genetic Architecture between Neighboring DNA-Methylation Sites
Heatmap of Bayesian co-localization results for all pairs of DNA-methylation sites with at least one significant mQTL ($p < 1 \times 10^{-10}$) in a genomic region on chromosome 9 (chr9:124783559–125216341). Columns and rows represent individual DNA-methylation sites (ordered by genomic location). The color of each square indicates the strength of the evidence for a shared genetic signal (from yellow [weak] to red [strong]); this strength is calculated as the ratio of the posterior probabilities that they share the same causal variant (PP4) compared to two distinct causal variants (PP3). The ratio was bounded to a maximum value of 10; gray indicates pairs of DNA-methylation sites that were not tested for co-localization.

genic features (OR = 1.73, Fisher's $p < 2.23 \times 10^{-308}$; Figure S12).

DNAm QTL Have Utility for Refining GWAS Signals for Complex Traits

Genetic variants identified in GWAS analyses rarely index protein-coding changes. Instead, they are hypothesized to influence gene regulation because they are enriched in regulatory motifs, including enhancers and regions of open chromatin.[24,26] There is considerable interest in using regulatory QTLs to refine genetic association signals and prioritize potentially causal genes within the extended genomic regions identified in GWASs.[17,27,48,49] We next extended our previous application of the SMR approach[28] to test 126,457 DNAm sites identified at our "discovery" mQTL threshold ($p < 1 \times 10^{-10}$) against 63 complex phenotypes with GWAS data (Table S5). The first stage of the SMR approach uses the most significantly associated mQTL SNP—that has also been tested in the GWAS dataset—as an instrumental variable and implements a two-step least-squares (2SLS) approach to compare the estimated associations. Using this approach, we identified 5,848 associations ($p < 3.95 \times 10^{-7}$ corrected for 126,457 DNAm sites) between 40 complex traits and 5,849 unique DNAm sites (Figure S13). Because the associations identified in this way potentially reflect two highly correlated but different causal variants for the GWAS trait and DNAm, the second stage of the SMR method repeats the analysis with alternative mQTL SNPs as the instrument. If there is a single causal variant associated with both the phenotype and DNAm, the association statistics will be identical regardless of the selected instrument. In contrast, if there are two separate causal variants, each correlated with the instrument, there will be variation in the results. To distinguish between these scenarios, we applied the heterogeneity in dependent instruments (HEIDI) test to select associations with non-significant heterogeneity (HEIDI $p > 0.05$) and identified a refined set of 1,662 associations between 36 complex traits and 1,246 DNAm sites (Table S6).

Because the power of the SMR approach to detect pleiotropic associations reflects, in part, the power of the initial complex-trait GWAS, it is unsurprising that the highest number of SMR associations was found for traits characterized by the largest number of GWAS signals, such as height (423 significant GWAS loci, 506 SMR pleiotropic associations)[50] and inflammatory bowel disease [MIM: 266600] (168 significant GWAS variants, 127 SMR pleiotropic associations).[51] In contrast, no SMR associations were found

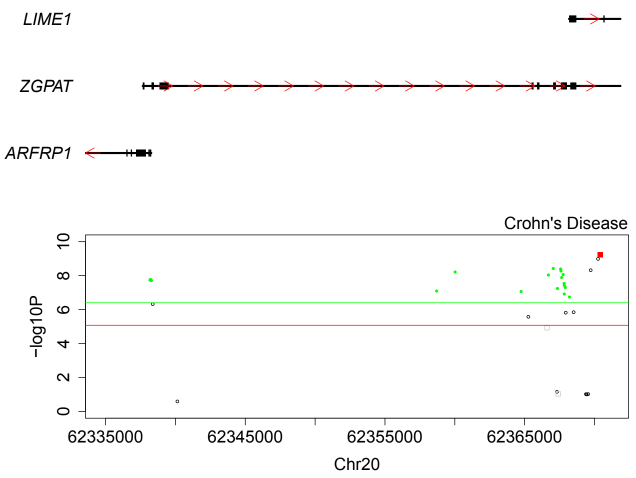

Figure 3. Summary-Data-Based Mendelian Randomization (SMR) Analysis Using Quantitative Trait Loci Associated with DNA Methylation (mQTL) and Gene Expression (eQTL) Implicates a Role for *LIME1* in Crohn Disease
Shown is a genomic region on chromosome 20 (chr20: 62335000–62371000) identified in a recent Crohn disease GWAS performed by Liu et al.[51] Genes located in this region are shown at the top, exons are indicated by thicker bars, and the red arrows indicate the direction of transcription. The scatterplot depicts the –log10 p value (y axis) against genomic location (x axis) from the SMR analysis (where circles represent Illumina EPIC array DNA-methylation sites, squares represent gene expression probes, and solid green and red highlight those with a non-significant HEIDI test for DNA methylation and gene expression, respectively). The green and red horizontal lines represent the multiple-testing corrected threshold for the SMR test using mQTL and eQTL, respectively.

for traits with few or no genome-wide-significant SNPs; such traits included parental age at death (0–1 significant GWAS variants),[52] insulin secretion rate (no significant GWAS variants),[53] and whether a person has ever smoked (no significant GWAS variants).[54] We compared our SMR results to those obtained with our previous mQTL dataset—generated from a smaller number of samples—and observed high rates of replication for loci that were tested in both analyses. Because our previous SMR analysis was based on a subset of 43 traits and the reduced content of the Illumina 450K array, 842 pleiotropic associations reported in the current analysis were taken forward for replication; DNAm at 519 (33.0%) of these was associated with an mQTL variant, and therefore these associations had been tested in our previous SMR study; 268 (51.6%) were characterized by significant pleiotropic association in both studies. Furthermore, the vast majority of associations tested in both datasets (516; 99.4%) were in the same direction; this was significantly more than would be expected by chance (sign test $p = 2.72 \times 10^{-149}$; Figure S14), suggesting that there are many additional true signals in those that did not meet the stringent criteria for significance used in both studies.

In order to prioritize genes for each complex trait, we characterized the genic location of associated DNAm sites. 1,269 (76.3%) of the identified pleiotropic associations involve DNAm sites located either within a gene or less than 1500 bp from the transcription start site; this rate is significantly higher rate than that for all DNAm sites tested in our SMR analysis (OR = 1.64, Fisher's test $p = 1.12 \times 10^{-18}$). To further explore these 786 pleiotropic associations—occurring between 577 genes and 32 complex traits—we extended our SMR analyses to incorporate a publicly available whole-blood gene eQTL (n = 5,311 individuals) dataset.[45] Expression of 232 (40.2%) of our identified genes was significantly associated with an eQTL variant, and we used these to test for pleiotropic associations between gene expression level and the GWAS trait. These analyses provided additional support for 138 of the pleiotropic associations identified with mQTL data, supporting a relationship between 33 genes and 17 complex traits (Table S7). Figure 3, for example, highlights an association between the regulation and expression of *LIME1* and Crohn disease [MIM: 266600]; this association is supported by SMR analyses incorporating both mQTL and eQTL data.

Pleiotropic Associations between DNAm and Gene Expression

Although it is widely hypothesized that DNAm influences gene expression, its relationship with transcriptional activity is not fully understood. DNAm across CpG-rich promoter regions, for example, is often assumed to repress gene expression via the blockage of transcription-factor binding and the attraction of methyl-binding proteins.[55] DNAm in the gene body, in contrast, is hypothesized to be a marker of active gene transcription[5,56] and to potentially play a role in regulating alternative splicing and isoform diversity. To identify associations between DNAm and gene expression, we applied the SMR approach to DNAm sites identified as being associated with an mQTL at our "discovery" significance threshold, located within a megabase of a gene expression probe included in the eQTL dataset generated by Westra and colleagues.[45] In total, we tested 488,342 pairs and explored relationships between 96,694 DNAm sites and 4,721 gene expression probes annotated to 4,049 genes (Figure S15). On average, each DNAm site was tested against a median of four expression probes (interquartile range = 2–7) mapping to a median of three genes (interquartile range = 2–6). In contrast, each expression probe was tested against a median of 85 DNAm sites (interquartile range = 56–130). Of these, 40,404 pairs (8.27%)—comprising 22,007 (22.8%) DNAm sites and 4,201 (89.0%) expression probes mapping to 3,628 (89.6%) genes—were characterized by a significant SMR result (significance threshold corrected for the number of DNAm sites and gene expression probe pairs tested = $p < 1.02 \times 10^{-7}$). 6,798 of these significant SMR pairs—comprising 5,420 (5.61%) DNAm sites and 1,913 (40.5%) expression probes mapping to 1,702 (42.0%) genes—also had a HEIDI $p > 0.05$ (Table S8; Figure S15). These results suggest that although expression of a large proportion of genes is associated with DNAm sites, not all DNAm sites are associated with gene expression in *cis*.

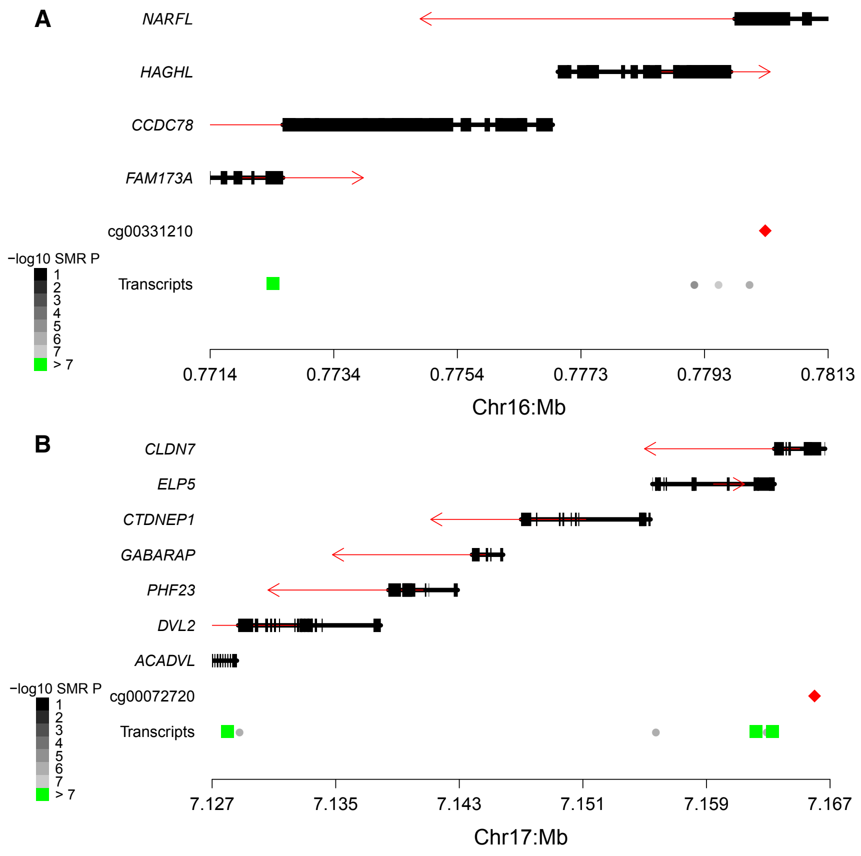

Figure 4. Regional Plots Demonstrating the Complex Relationship between Gene Expression and DNA Methylation as Identified by SMR

Shown is an example of (A) a DNA-methylation site (cg00331210) that is associated with expression of a gene (*FAM173A*) that is not the most proximal to it and (B) a DNA-methylation site (cg00072720) associated with the expression of multiple genes (*CLDN7* and *ELP5*). Each plot contains a gene track, where red arrows indicate the direction of transcription and a red diamond indicates the position of the pleiotropically significant DNA-methylation site. Circles and squares indicate the location of the gene expression probes that DNA-methylation sites were tested against. Color indicates the significance level of the SMR test (black to gray), and green indicates significant associations ($p < 1.02 \times 10^{-7}$). For significant associations, squares indicate tests that have non-significant heterogeneity ($p > 0.05$) and are indicative of pleiotropic associations.

The majority of significant gene expression probes (n = 1,192; 62.3%) are associated with a median of two DNAm sites (interquartile range = 1–4) spanning a median distance of 66,846 bp (interquartile range = 19,062–155,737) at a median density of 19,959 bp (interquartile range = 6,387–54,445) between sites. Interestingly, DNAm sites pleiotropically associated with gene expression are enriched in the gene body and transcription start sites of genes and depleted intergenically (Chi square test $p = 7.08 \times 10^{-133}$; Figure S16; Table S9). We identified a small but significant enrichment of scenarios where DNAm is negatively associated with gene expression at sites located in the 5′ UTR (mean effect = −0.0211; p = 0.00108), TSS200 (mean effect = −0.0479; $p = 6.38 \times 10^{-7}$), TSS1500 (mean effect = −0.0350; $p = 5.82 \times 10^{-11}$) and 1st exon (mean effect = −0.0506; $p = 6.19 \times 10^{-5}$), consistent with the hypothesis that promoter DNAm often represses gene expression (Figure S17).

Using QTL Data to Refine the Genic Annotation Associated with DNAm Sites

A key challenge in epigenetic epidemiology relates to the genic annotation of DNAm sites; such annotation is critical for the biological interpretation of significant EWAS associations. DNAm sites are usually annotated to specific genes on the basis of proximity, although the extent to which this approach is valid for inferring downstream transcriptional effects is not known. Among the identified pleiotropic associations between DNAm and gene expression, we selected instances where the DNAm site is not intergenic—i.e., <1500 bp from the transcription start site of a gene (n = 5,593 [82.3%])—and found that these were annotated to the same gene whose expression level they were associated with at a much higher rate than were DNAm sites significantly associated with expression levels at another gene (OR = 9.67; Fisher's test $p < 2.23 \times 10^{-308}$). Of the 5,460 DNAm sites significantly associated with expression of at least one gene, 1,790 (32.8%) were associated with the gene they were annotated to, although 276 (5.05%) of these were also associated to an additional gene and 2,686 (50.0%) were associated with a different gene. Of note, not all CpGs were tested against the gene they were annotated to because the gene lacked a significant eQTL; this was the case for the majority of DNAm sites (n = 2,701; 80.4%) identified as being associated with a gene other than the one they were annotated to. Of particular interest are the 944 (18.3%) intergenic sites that are associated with gene expression; these potentially enable additional gene annotations for interpreting the results of EWAS analyses. Overall, although the proximity-based annotation of DNAm sites appears to be appropriate in many instances, we identified notable exceptions. For example, Figure 4A shows that the DNAm site cg00331210, located within the body of *NARFL* on chromosome 16, is not associated with expression of that gene but with the *FAM173A* gene, which is located 7.9 kilobases away. Likewise, Figure 4B shows that the DNAm site cg00072720, located within the gene body of *CLDN7*, is not associated with expression of that gene but with that of two other genes (*ACADVL* and *ELP5/C17ORF81*) on chromosome 17.

Discussion

In this study we present a comprehensive assessment of the genetic architecture of DNAm and identify associations between common genetic variants and specific DNAm sites (mQTLs) by using the Illumina EPIC array. We utilized our database of mQTL associations to characterize genetic influences on individual and proximally located DNAm sites. We show that there are many instances of shared genetic signals on neighboring DNAm sites and that these associations are structured around both genes and CpG islands. Our results are in line with the GeMes groups reported by Liu et al., who observed that multiple DNAm sites were influenced by overlapping genetic variants; their observations included examples where these DNAm sites were not contiguous.[46] Moreover, we report that these shared genetic effects on DNAm are generally associated with positive correlations between the DNAm sites. This has implications for studies of trait-associated differentially methylated regions (DMRs) because it suggests that associations with phenotypic variation could be genetically mediated.

In an extension of our work prioritizing genes in GWAS-nominated regions,[28] we found robust agreement with our previous SMR findings (obtained from mQTLs identified with the Illumina 450K array) for shared content by using independent datasets. The additional content present on the EPIC array, however, enabled us to identify gene-trait associations not detected with the older array technology, increasing the potential yield of biological information. This augments the existing literature integrating results from GWASs of complex traits and quantitative trait loci (QTL) studies of gene expression and DNA methylation[27,57–59] and substantiates the hypothesis that GWAS variants act via gene regulation. Finally, we use these data to explore the relationship between DNAm and gene expression by using genetic instruments rather than correlations to infer associations between specific DNAm sites and genes. Although most DNAm sites associated with gene expression were found to be located within the gene body or close to the transcription start site, there are many relationships that challenge the commonly used genic annotation on the sole basis of physical proximity. Furthermore, although the expression of most genes is associated with one or more DNAm sites, not all DNAm sites are associated with gene expression, implying that variable DNAm does not always have an effect on gene expression. These findings are consistent with those reported previously by Bonder et al.[60] in their expression quantitative trait methylation (eQTM) analysis; they also report the association of multiple DNAm sites with each gene, the presence of both negative and positive correlations between DNAm and gene expression, and an enrichment of DNAm sites associated with gene expression in the TSS and enhancers. Although we could only test for associations between DNAm sites with significant mQTLs and the expression of genes with a significant eQTL, our results provide a potentially effective method for annotating results from EWAS, particularly where the influence of DNAm on gene expression is hypothesized and candidates are taken forward for transcriptional analysis.

Our study has a number of important limitations. The analyses presented here are based on an unrelated subset of participants from the UKHLS; although these represent a large sample (>1,000) of European ancestry with a broad age range, the extent to which our results are applicable to other ethnic groups characterized by a different genetic architecture is not known. Despite using the most comprehensive, high-throughput technology for profiling DNAm across the genome (the Illumina EPIC array), our study only assayed a small proportion of the total number of DNAm sites and included sparse coverage of regulatory features that are often represented by a single DNAm site.[40] Moreover, DNAm was profiled in whole blood, which potentially limits the interpretation of candidate disease genes where the presumed tissue of interest is not blood. Given the tissue-specific nature of some mQTL and eQTL effects, these associations should be confirmed in additional disease-relevant tissues and cell types. Although Mendelian randomization is proposed as a methodology for quantifying causal relationships between variables, it relies on a number of key assumptions,[61] all of which also apply to SMR. Therefore, our approach did not seek to establish the direction of association between DNA methylation and outcome; we are consequently careful in our use of terminology and refrain from describing our associations as “causal,” especially because the SMR approach is unable to distinguish two causal variants in approximately perfect LD from one causal variant;[27] instead, we refer to these as “pleiotropic” associations. Furthermore, given that our application of MR is based on a single genetic variant, we cannot rule out the possibility of horizontal pleiotropy. Finally, a limitation of the HEIDI approach to distinguishing pleiotropic associations from LD artifacts is that it looks to accept the null hypothesis of homogeneity of effects rather than reject it. However, we are confident in the set of pleiotropic associations we report given the strong replication of our previous results based on mQTLs estimated in an independent dataset.[28]

Taken together, our results add to an increasing body of evidence showing that genetic influences on DNA methylation are widespread across the genome. We show that integrating these relationships with the results from GWAS of complex traits and genetic studies of gene expression can improve our understanding about the interplay between gene regulation and expression and facilitate the prioritization of candidate genes implicated in disease etiology.

Accession Numbers

Individual-level DNA methylation and genetic data are available upon application through the European Genome-Phenome Archive under accession code EGAS00001001232.

Supplemental Data

Supplemental Data include seventeen figures and nine tables and can be found with this article online at https://doi.org/10.1016/j.ajhg.2018.09.007.

Acknowledgments

We acknowledge the Wellcome Trust Sanger Institute and Ele Zeggini for generating the genotype data. Both genotyping and DNA methylation in UKHLS were funded through enhancements to the Economic and Social Research Council (ESRC) grants ES/K005146/1 and ES/N00812X/1. A.H., M.C.S., and Y.B. are supported by the ESRC (ES/M008592/1). M.K. is supported by the University of Essex and ESRC (RES-596-28-0001). E.H., J.M., and L.C.S. were supported by Medical Research Council grant K013807 to J.M.. Analysis was facilitated by access to the Genome High-Performance Computing Cluster at the University of Essex School of Biological Sciences.

Declaration of Interests

The authors declare no conflict of interest.

Received: June 15, 2018
Accepted: September 14, 2018
Published: October 25, 2018

Web Resources

Complex Disease Epigenomics Group, http://www.epigenomicslab.com/online-data-resources/
European Genome-Phenome Archive (EGA), https://www.ebi.ac.uk/ega/home
GitHub, https://github.com/ejh243/UKHLS_mQTL.git
Illumina product support, http://emea.support.illumina.com/array/array_kits/infinium-methylationepic-beadchip-kit/downloads.html#
METADAC, www.metadac.ac.uk
Online Mendelian Inheritance in Man: http://www.omim.org
R *coloc* package, http://cran.r-project.org/web/packages/coloc
SMR, http://cnsgenomics.com/software/smr/download.html
Understanding Society, https://www.understandingsociety.ac.uk

References

1. Bird, A. (2002). DNA methylation patterns and epigenetic memory. Genes Dev. *16*, 6–21.
2. Jones, P.A. (2012). Functions of DNA methylation: islands, start sites, gene bodies and beyond. Nat. Rev. Genet. *13*, 484–492.
3. Wagner, J.R., Busche, S., Ge, B., Kwan, T., Pastinen, T., and Blanchette, M. (2014). The relationship between DNA methylation, genetic and expression inter-individual variation in untransformed human fibroblasts. Genome Biol. *15*, R37.
4. Yang, X., Han, H., De Carvalho, D.D., Lay, F.D., Jones, P.A., and Liang, G. (2014). Gene body methylation can alter gene expression and is a therapeutic target in cancer. Cancer Cell *26*, 577–590.
5. Maunakea, A.K., Nagarajan, R.P., Bilenky, M., Ballinger, T.J., D'Souza, C., Fouse, S.D., Johnson, B.E., Hong, C., Nielsen, C., Zhao, Y., et al. (2010). Conserved role of intragenic DNA methylation in regulating alternative promoters. Nature *466*, 253–257.
6. Moen, E.L., Zhang, X., Mu, W., Delaney, S.M., Wing, C., McQuade, J., Myers, J., Godley, L.A., Dolan, M.E., and Zhang, W. (2013). Genome-wide variation of cytosine modifications between European and African populations and the implications for complex traits. Genetics *194*, 987–996.
7. Zhao, L., Liu, D., Xu, J., Wang, Z., Chen, Y., Lei, C., Li, Y., Liu, G., and Jiang, Y. (2018). The framework for population epigenetic study. Brief. Bioinform. *19*, 89–100.
8. Baylin, S.B., and Jones, P.A. (2016). Epigenetic determinants of cancer. Cold Spring Harb. Perspect. Biol. *8*, 8.
9. Liu, Y., Aryee, M.J., Padyukov, L., Fallin, M.D., Hesselberg, E., Runarsson, A., Reinius, L., Acevedo, N., Taub, M., Ronninger, M., et al. (2013). Epigenome-wide association data implicate DNA methylation as an intermediary of genetic risk in rheumatoid arthritis. Nat. Biotechnol. *31*, 142–147.
10. Wahl, S., Drong, A., Lehne, B., Loh, M., Scott, W.R., Kunze, S., Tsai, P.C., Ried, J.S., Zhang, W., Yang, Y., et al. (2017). Epigenome-wide association study of body mass index, and the adverse outcomes of adiposity. Nature *541*, 81–86.
11. Hannon, E., Dempster, E., Viana, J., Burrage, J., Smith, A.R., Macdonald, R., St Clair, D., Mustard, C., Breen, G., Therman, S., et al. (2016). An integrated genetic-epigenetic analysis of schizophrenia: evidence for co-localization of genetic associations and differential DNA methylation. Genome Biol. *17*, 176.
12. Lunnon, K., Smith, R., Hannon, E., De Jager, P.L., Srivastava, G., Volta, M., Troakes, C., Al-Sarraj, S., Burrage, J., Macdonald, R., et al. (2014). Methylomic profiling implicates cortical deregulation of ANK1 in Alzheimer's disease. Nat. Neurosci. *17*, 1164–1170.
13. van Dongen, J., Nivard, M.G., Willemsen, G., Hottenga, J.J., Helmer, Q., Dolan, C.V., Ehli, E.A., Davies, G.E., van Iterson, M., Breeze, C.E., et al.; BIOS Consortium (2016). Genetic and environmental influences interact with age and sex in shaping the human methylome. Nat. Commun. *7*, 11115.
14. Bell, J.T., Tsai, P.C., Yang, T.P., Pidsley, R., Nisbet, J., Glass, D., Mangino, M., Zhai, G., Zhang, F., Valdes, A., et al.; MuTHER Consortium (2012). Epigenome-wide scans identify differentially methylated regions for age and age-related phenotypes in a healthy ageing population. PLoS Genet. *8*, e1002629.
15. Grundberg, E., Meduri, E., Sandling, J.K., Hedman, A.K., Keildson, S., Buil, A., Busche, S., Yuan, W., Nisbet, J., Sekowska, M., et al.; Multiple Tissue Human Expression Resource Consortium (2013). Global analysis of DNA methylation variation in adipose tissue from twins reveals links to disease-associated variants in distal regulatory elements. Am. J. Hum. Genet. *93*, 876–890.
16. McRae, A.F., Powell, J.E., Henders, A.K., Bowdler, L., Hemani, G., Shah, S., Painter, J.N., Martin, N.G., Visscher, P.M., and Montgomery, G.W. (2014). Contribution of genetic variation to transgenerational inheritance of DNA methylation. Genome Biol. *15*, R73.
17. Hannon, E., Spiers, H., Viana, J., Pidsley, R., Burrage, J., Murphy, T.M., Troakes, C., Turecki, G., O'Donovan, M.C., Schalkwyk, L.C., et al. (2016). Methylation QTLs in the developing brain and their enrichment in schizophrenia risk loci. Nat. Neurosci. *19*, 48–54. Published online November 30, 2015.
18. Gibbs, J.R., van der Brug, M.P., Hernandez, D.G., Traynor, B.J., Nalls, M.A., Lai, S.L., Arepalli, S., Dillman, A., Rafferty, I.P., Troncoso, J., et al. (2010). Abundant quantitative trait loci exist for DNA methylation and gene expression in human brain. PLoS Genet. *6*, e1000952.

19. Gamazon, E.R., Badner, J.A., Cheng, L., Zhang, C., Zhang, D., Cox, N.J., Gershon, E.S., Kelsoe, J.R., Greenwood, T.A., Nievergelt, C.M., et al. (2013). Enrichment of cis-regulatory gene expression SNPs and methylation quantitative trait loci among bipolar disorder susceptibility variants. Mol. Psychiatry *18*, 340–346.
20. Drong, A.W., Nicholson, G., Hedman, A.K., Meduri, E., Grundberg, E., Small, K.S., Shin, S.Y., Bell, J.T., Karpe, F., Soranzo, N., et al.; MolPAGE Consortia (2013). The presence of methylation quantitative trait loci indicates a direct genetic influence on the level of DNA methylation in adipose tissue. PLoS ONE *8*, e55923.
21. Gaunt, T.R., Shihab, H.A., Hemani, G., Min, J.L., Woodward, G., Lyttleton, O., Zheng, J., Duggirala, A., McArdle, W.L., Ho, K., et al. (2016). Systematic identification of genetic influences on methylation across the human life course. Genome Biol. *17*, 61.
22. Olsson, A.H., Volkov, P., Bacos, K., Dayeh, T., Hall, E., Nilsson, E.A., Ladenvall, C., Rönn, T., and Ling, C. (2014). Genome-wide associations between genetic and epigenetic variation influence mRNA expression and insulin secretion in human pancreatic islets. PLoS Genet. *10*, e1004735.
23. Gutierrez-Arcelus, M., Lappalainen, T., Montgomery, S.B., Buil, A., Ongen, H., Yurovsky, A., Bryois, J., Giger, T., Romano, L., Planchon, A., et al. (2013). Passive and active DNA methylation and the interplay with genetic variation in gene regulation. eLife *2*, e00523.
24. Schaub, M.A., Boyle, A.P., Kundaje, A., Batzoglou, S., and Snyder, M. (2012). Linking disease associations with regulatory information in the human genome. Genome Res. *22*, 1748–1759.
25. Ernst, J., Kheradpour, P., Mikkelsen, T.S., Shoresh, N., Ward, L.D., Epstein, C.B., Zhang, X., Wang, L., Issner, R., Coyne, M., et al. (2011). Mapping and analysis of chromatin state dynamics in nine human cell types. Nature *473*, 43–49.
26. Maurano, M.T., Humbert, R., Rynes, E., Thurman, R.E., Haugen, E., Wang, H., Reynolds, A.P., Sandstrom, R., Qu, H., Brody, J., et al. (2012). Systematic localization of common disease-associated variation in regulatory DNA. Science *337*, 1190–1195.
27. Zhu, Z., Zhang, F., Hu, H., Bakshi, A., Robinson, M.R., Powell, J.E., Montgomery, G.W., Goddard, M.E., Wray, N.R., Visscher, P.M., and Yang, J. (2016). Integration of summary data from GWAS and eQTL studies predicts complex trait gene targets. Nat. Genet. *48*, 481–487.
28. Hannon, E., Weedon, M., Bray, N., O'Donovan, M., and Mill, J. (2017). Pleiotropic Effects of Trait-Associated Genetic Variation on DNA Methylation: Utility for Refining GWAS Loci. Am. J. Hum. Genet. *100*, 954–959.
29. Knies, G. (2015). Understanding Society—UK Household Longitudinal Study: Wave 1–5, User Manual (University of Essex).
30. Benzeval, M., Davillas, A., Kumari, M., and Lynn, P. (2014). Understanding Society: The UK Household Longitudinal Study Biomarker User Guide and Glossary (Institute for Social and Economic Research, University of Essex).
31. R Development Core Team (2008). R: A Language and Environment for Statistical Computing (Vienna, Austria: R Foundation for Statistical Computing).
32. Gorrie-Stone, T.J., Smart, M.C., Saffari, A., Malki, K., Hannon, E., Burrage, J., Mill, J., Kumari, M., and Schalkwyk, L.C. (2018). Bigmelon: Tools for analysing large DNA methylation datasets. Bioinformatics, bty713. Published online August 23, 2018. https://doi.org/10.1093/bioinformatics/bty713.
33. Horvath, S. (2013). DNA methylation age of human tissues and cell types. Genome Biol. *14*, R115.
34. Pidsley, R., Y Wong, C.C., Volta, M., Lunnon, K., Mill, J., and Schalkwyk, L.C. (2013). A data-driven approach to preprocessing Illumina 450K methylation array data. BMC Genomics *14*, 293.
35. Prins, B.P., Kuchenbaecker, K.B., Bao, Y., Smart, M., Zabaneh, D., Fatemifar, G., Luan, J., Wareham, N.J., Scott, R.A., Perry, J.R.B., et al. (2017). Genome-wide analysis of health-related biomarkers in the UK Household Longitudinal Study reveals novel associations. Sci. Rep. *7*, 11008.
36. Purcell, S., Neale, B., Todd-Brown, K., Thomas, L., Ferreira, M.A., Bender, D., Maller, J., Sklar, P., de Bakker, P.I., Daly, M.J., and Sham, P.C. (2007). PLINK: a tool set for whole-genome association and population-based linkage analyses. Am. J. Hum. Genet. *81*, 559–575.
37. Das, S., Forer, L., Schönherr, S., Sidore, C., Locke, A.E., Kwong, A., Vrieze, S.I., Chew, E.Y., Levy, S., McGue, M., et al. (2016). Next-generation genotype imputation service and methods. Nat. Genet. *48*, 1284–1287.
38. Yang, J., Lee, S.H., Goddard, M.E., and Visscher, P.M. (2011). GCTA: A tool for genome-wide complex trait analysis. Am. J. Hum. Genet. *88*, 76–82.
39. McCartney, D.L., Walker, R.M., Morris, S.W., McIntosh, A.M., Porteous, D.J., and Evans, K.L. (2016). Identification of polymorphic and off-target probe binding sites on the Illumina Infinium MethylationEPIC BeadChip. Genom. Data *9*, 22–24.
40. Pidsley, R., Zotenko, E., Peters, T.J., Lawrence, M.G., Risbridger, G.P., Molloy, P., Van Djik, S., Muhlhausler, B., Stirzaker, C., and Clark, S.J. (2016). Critical evaluation of the Illumina MethylationEPIC BeadChip microarray for whole-genome DNA methylation profiling. Genome Biol. *17*, 208.
41. Shabalin, A.A. (2012). Matrix eQTL: Ultra fast eQTL analysis via large matrix operations. Bioinformatics *28*, 1353–1358.
42. Houseman, E.A., Accomando, W.P., Koestler, D.C., Christensen, B.C., Marsit, C.J., Nelson, H.H., Wiencke, J.K., and Kelsey, K.T. (2012). DNA methylation arrays as surrogate measures of cell mixture distribution. BMC Bioinformatics *13*, 86.
43. Koestler, D.C., Christensen, B., Karagas, M.R., Marsit, C.J., Langevin, S.M., Kelsey, K.T., Wiencke, J.K., and Houseman, E.A. (2013). Blood-based profiles of DNA methylation predict the underlying distribution of cell types: a validation analysis. Epigenetics *8*, 816–826.
44. Giambartolomei, C., Vukcevic, D., Schadt, E.E., Franke, L., Hingorani, A.D., Wallace, C., and Plagnol, V. (2014). Bayesian test for colocalisation between pairs of genetic association studies using summary statistics. PLoS Genet. *10*, e1004383.
45. Westra, H.J., Peters, M.J., Esko, T., Yaghootkar, H., Schurmann, C., Kettunen, J., Christiansen, M.W., Fairfax, B.P., Schramm, K., Powell, J.E., et al. (2013). Systematic identification of trans eQTLs as putative drivers of known disease associations. Nat. Genet. *45*, 1238–1243.
46. Liu, Y., Li, X., Aryee, M.J., Ekström, T.J., Padyukov, L., Klareskog, L., Vandiver, A., Moore, A.Z., Tanaka, T., Ferrucci, L., et al. (2014). GeMes, clusters of DNA methylation under genetic control, can inform genetic and epigenetic analysis of disease. Am. J. Hum. Genet. *94*, 485–495.
47. Guo, H., Fortune, M.D., Burren, O.S., Schofield, E., Todd, J.A., and Wallace, C. (2015). Integration of disease association and eQTL data using a Bayesian colocalisation approach highlights six candidate causal genes in immune-mediated diseases. Hum. Mol. Genet. *24*, 3305–3313.

48. Mancuso, N., Shi, H., Goddard, P., Kichaev, G., Gusev, A., and Pasaniuc, B. (2017). Integrating gene expression with summary association statistics to identify genes associated with 30 complex traits. Am. J. Hum. Genet. *100*, 473–487.
49. Hauberg, M.E., Zhang, W., Giambartolomei, C., Franzén, O., Morris, D.L., Vyse, T.J., Ruusalepp, A., Sklar, P., Schadt, E.E., Björkegren, J.L.M., Roussos, P.; and CommonMind Consortium (2017). Large-scale identification of common trait and disease variants affecting gene expression. Am. J. Hum. Genet. *101*, 157.
50. Wood, A.R., Esko, T., Yang, J., Vedantam, S., Pers, T.H., Gustafsson, S., Chu, A.Y., Estrada, K., Luan, J., Kutalik, Z., et al.; Electronic Medical Records and Genomics (eMEMERGEGE) Consortium; MIGen Consortium; PAGEGE Consortium; and LifeLines Cohort Study (2014). Defining the role of common variation in the genomic and biological architecture of adult human height. Nat. Genet. *46*, 1173–1186.
51. Liu, J.Z., van Sommeren, S., Huang, H., Ng, S.C., Alberts, R., Takahashi, A., Ripke, S., Lee, J.C., Jostins, L., Shah, T., et al.; International Multiple Sclerosis Genetics Consortium; and International IBD Genetics Consortium (2015). Association analyses identify 38 susceptibility loci for inflammatory bowel disease and highlight shared genetic risk across populations. Nat. Genet. *47*, 979–986.
52. Pilling, L.C., Atkins, J.L., Bowman, K., Jones, S.E., Tyrrell, J., Beaumont, R.N., Ruth, K.S., Tuke, M.A., Yaghootkar, H., Wood, A.R., et al. (2016). Human longevity is influenced by many genetic variants: evidence from 75,000 UK Biobank participants. Aging (Albany NY) *8*, 547–560.
53. Wood, A.R., Jonsson, A., Jackson, A.U., Wang, N., van Leewen, N., Palmer, N.D., Kobes, S., Deelen, J., Boquete-Vilarino, L., Paananen, J., et al.; Diabetes Research on Patient Stratification (DIRECT) (2017). A genome-wide association study of IVGTT-based measures of first-phase insulin secretion refines the underlying physiology of type 2 diabetes variants. Diabetes *66*, 2296–2309.
54. Tobacco and Genetics Consortium (2010). Genome-wide meta-analyses identify multiple loci associated with smoking behavior. Nat. Genet. *42*, 441–447.
55. Bogdanović, O., and Veenstra, G.J. (2009). DNA methylation and methyl-CpG binding proteins: developmental requirements and function. Chromosoma *118*, 549–565.
56. Ball, M.P., Li, J.B., Gao, Y., Lee, J.H., LeProust, E.M., Park, I.H., Xie, B., Daley, G.Q., and Church, G.M. (2009). Targeted and genome-scale strategies reveal gene-body methylation signatures in human cells. Nat. Biotechnol. *27*, 361–368.
57. Richardson, T.G., Haycock, P.C., Zheng, J., Timpson, N.J., Gaunt, T.R., Davey Smith, G., Relton, C.L., and Hemani, G. (2018). Systematic Mendelian randomization framework elucidates hundreds of CpG sites which may mediate the influence of genetic variants on disease. Hum. Mol. Genet. *27*, 3293–3304.
58. Wu, Y., Zeng, J., Zhang, F., Zhu, Z., Qi, T., Zheng, Z., Lloyd-Jones, L.R., Marioni, R.E., Martin, N.G., Montgomery, G.W., et al. (2018). Integrative analysis of omics summary data reveals putative mechanisms underlying complex traits. Nat. Commun. *9*, 918.
59. Zhu, Z., Zheng, Z., Zhang, F., Wu, Y., Trzaskowski, M., Maier, R., Robinson, M.R., McGrath, J.J., Visscher, P.M., Wray, N.R., and Yang, J. (2018). Causal associations between risk factors and common diseases inferred from GWAS summary data. Nat. Commun. *9*, 224.
60. Bonder, M.J., Luijk, R., Zhernakova, D.V., Moed, M., Deelen, P., Vermaat, M., van Iterson, M., van Dijk, F., van Galen, M., Bot, J., et al.; BIOS Consortium (2017). Disease variants alter transcription factor levels and methylation of their binding sites. Nat. Genet. *49*, 131–138.
61. Smith, G.D., and Ebrahim, S. (2003). 'Mendelian randomization': Can genetic epidemiology contribute to understanding environmental determinants of disease? Int. J. Epidemiol. *32*, 1–22.

The American Journal of Human Genetics, Volume *103*

Supplemental Data

Leveraging DNA-Methylation Quantitative-Trait Loci to Characterize the Relationship between Methylomic Variation, Gene Expression, and Complex Traits

Eilis Hannon, Tyler J. Gorrie-Stone, Melissa C. Smart, Joe Burrage, Amanda Hughes, Yanchun Bao, Meena Kumari, Leonard C. Schalkwyk, and Jonathan Mill

Figure S1: Overview of the study providing a schematic of our analytical plan and the datasets used in analyses.

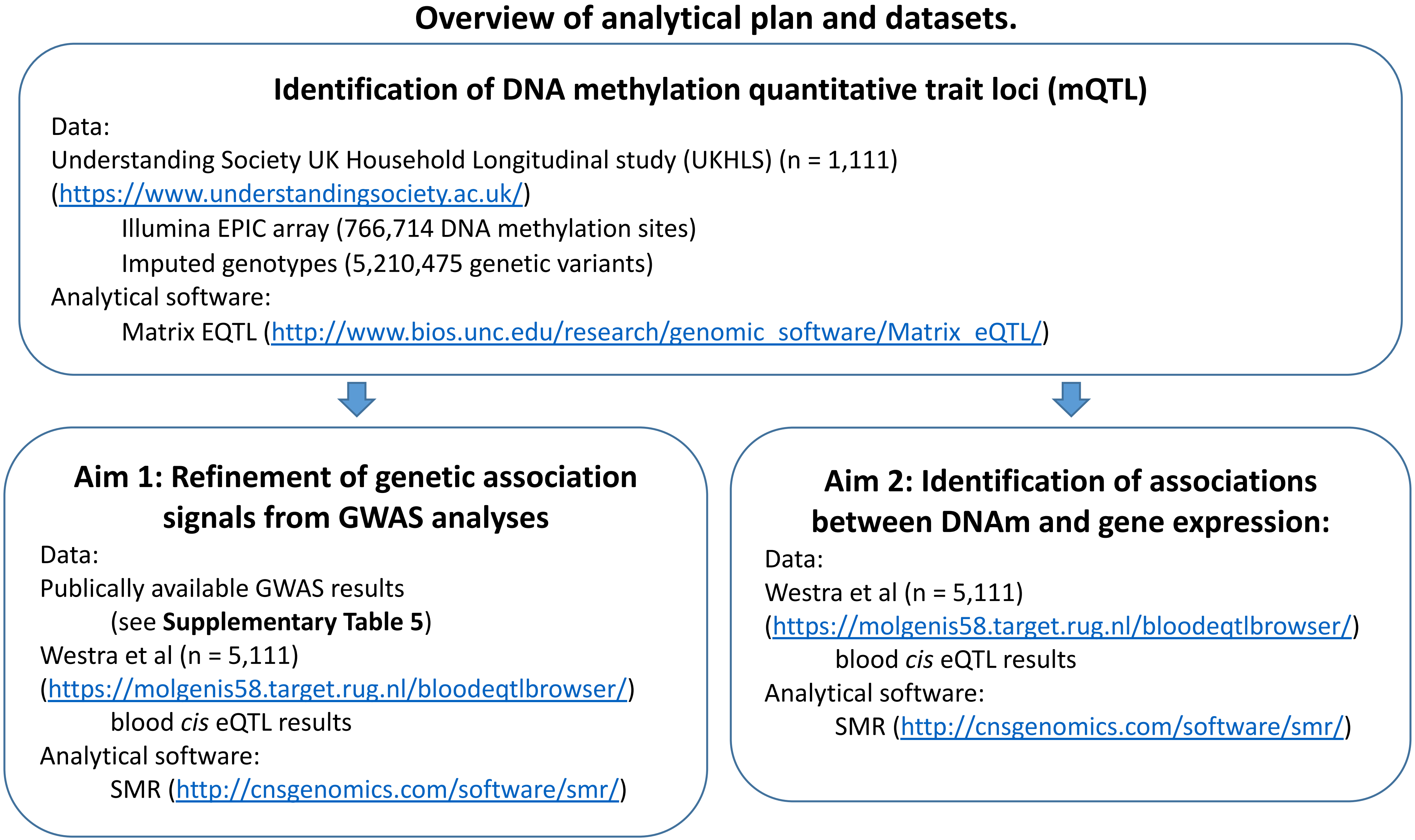

Figure S2: Genic distribution of novel associations between genetic variation and DNA methylation using the Illumina EPIC array. Barplot demonstrating the genic feature annotation of DNA methylation sites associated with common genetic variation split by whether the DNA methylation site was part of the older 450K array (red) or novel to the EPIC array (blue).

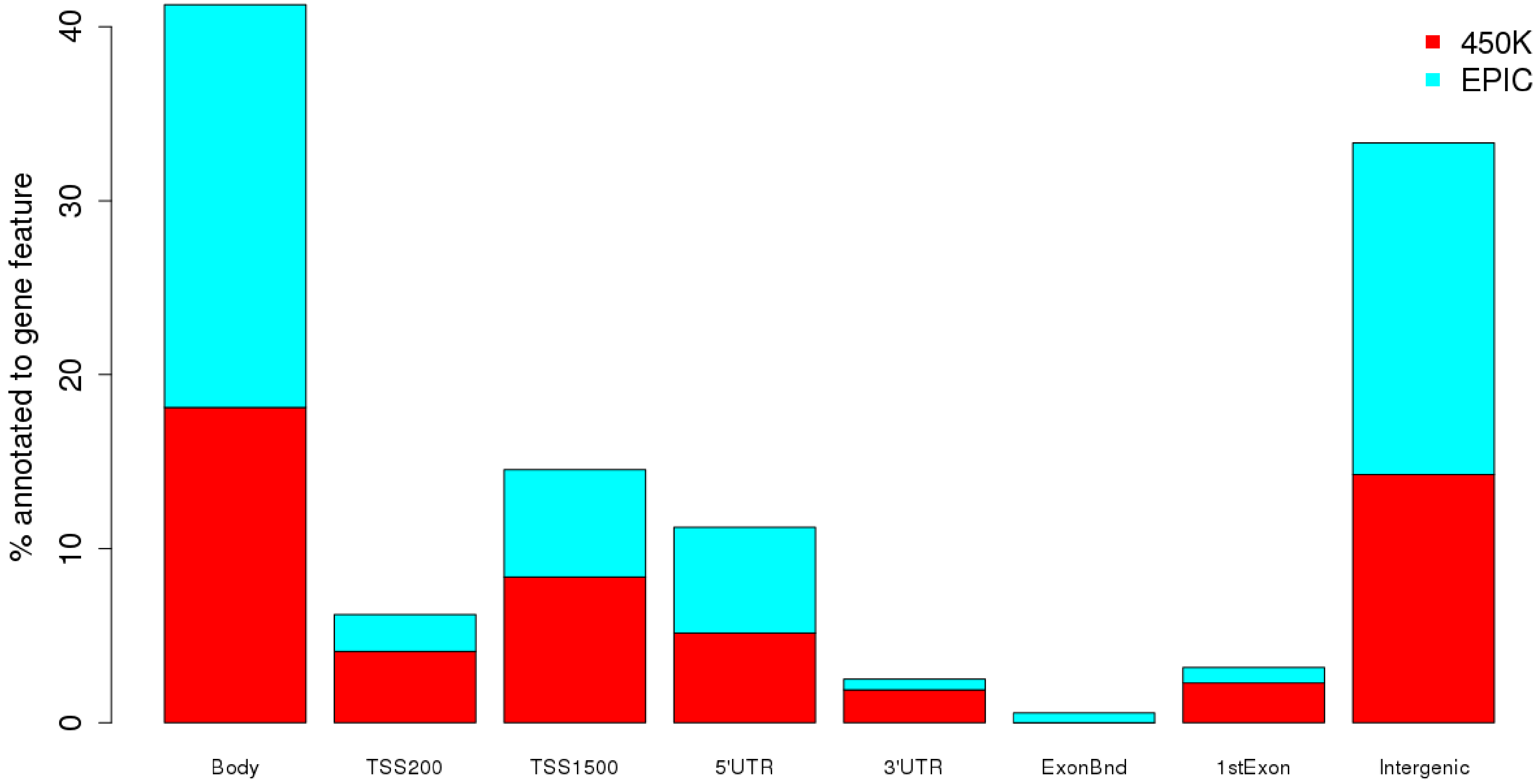

Figure S3: Additional content on the Illumina EPIC array enable the identification of novel DNA methylation quantitative trait loci (mQTL) associations. Venn diagram of the overlap of genes annotated to genetically influenced DNA methylation sites (identified at $P < 6.52x10^{-14}$) through content included on the 450K array and novel content added to the EPIC array. The outside circle includes all genes annotated to any DNA methylation site tested.

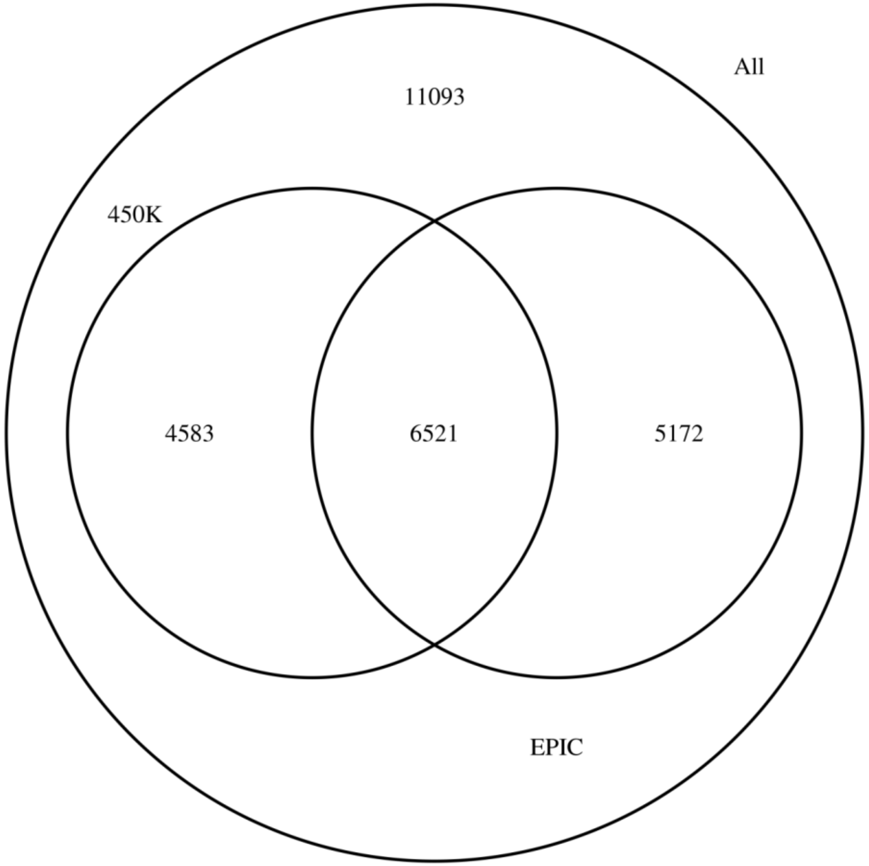

Figure S4: Frequency distribution of DNA methylation quantitative trait loci (mQTL) SNPs and their associated DNA methylation sites. Histograms of **A**) the number of independent genetic variants an individual DNA methylation site is associated with and **B**) the number of DNA methylation sites a genetic variant is associated with. Genetically-mediated DNA methylation sites are often associated with multiple mQTL SNPs (likely reflecting an artefact of linkage disequilibrium), but mQTL SNPs are associated with DNA methylation at relatively few sites.

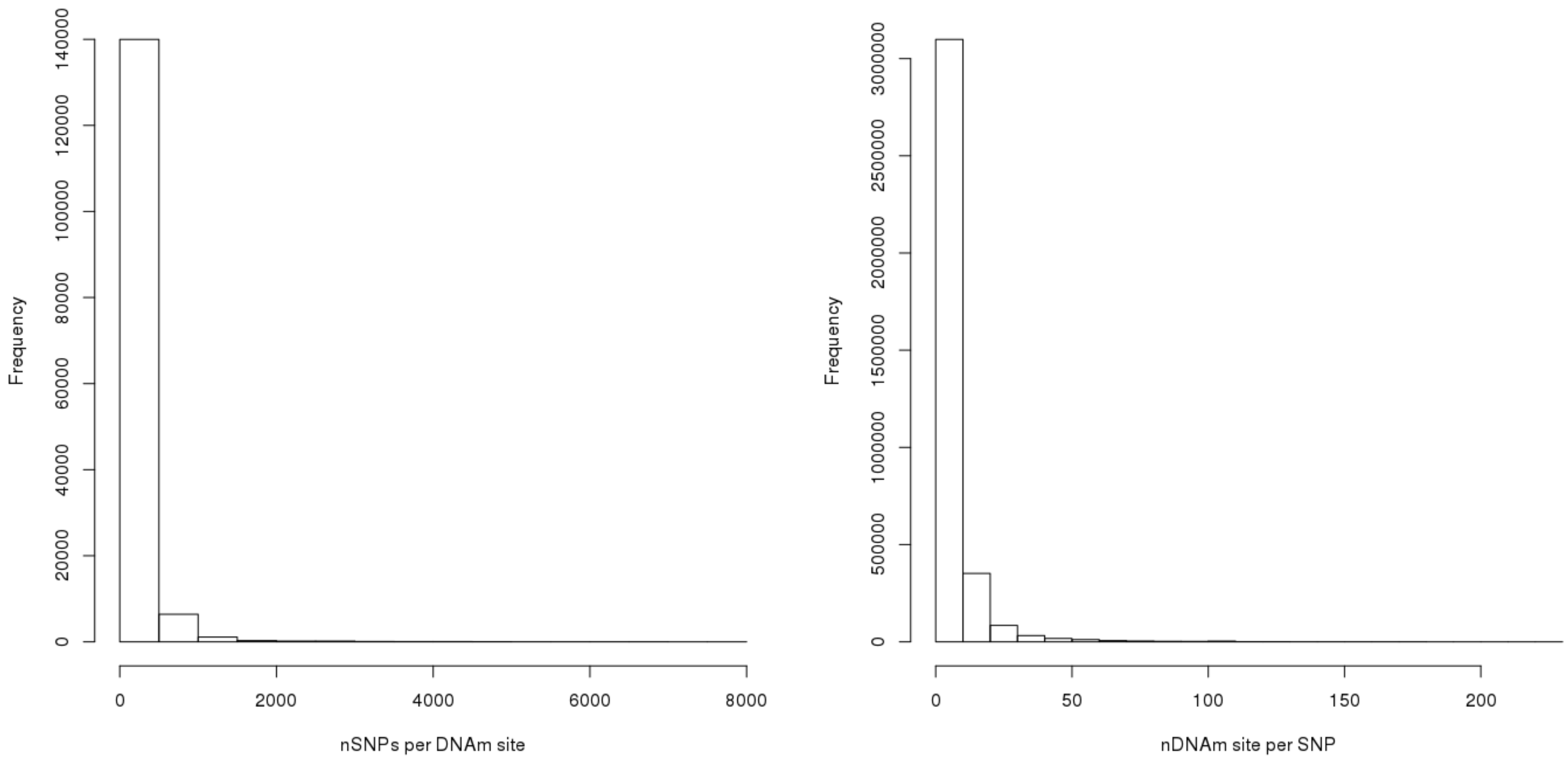

Figure S5: Frequency distribution of DNA methylation quantitative trait loci (mQTL) SNPs and associated DNA methylation sites after filtering for independent associations. Histograms of **A**) the number of independent genetic variants a DNA methylation site is associated with and **B**) the number of DNA methylation sites a genetic variant is associated with. Independent genetic signals were identified by clumping DNA methylation quantitative trait loci with a Bonferroni corrected P value of $P < 6.52 \times 10^{-14}$. After controlling for correlated genetic signals, each DNA methylation site is associated with a median of 1 genetic variant, and each genetic variant is associated with a median of 1 DNA methylation site.

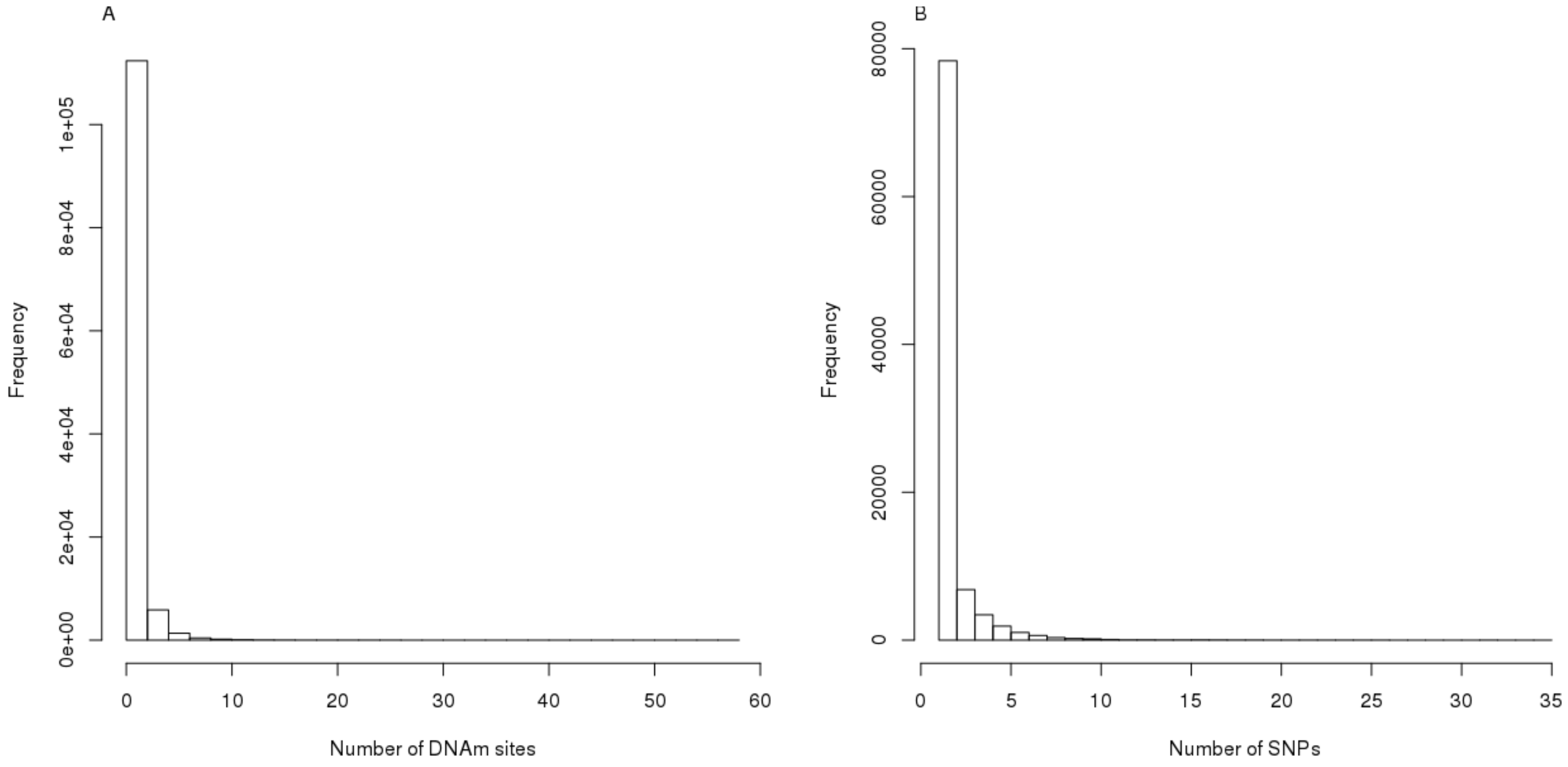

Figure S6: The distribution of effect sizes across all Bonferroni significant DNA methylation quantitative trait loci (mQTL). Shown is the DNA methylation difference (% DNA methylation) associated with each minor allele for all mQTLs ($P < 6.52 \times 10^{-14}$), *cis*-acting mQTLs, and *trans*-acting mQTLs. The average effect size for *trans*-mQTLs (3.26% (SD = 2.78%) per allele) is significantly lower than that observed for *cis*-mQTLs (3.48% (SD = 3.03%) per allele) (two-sided Wilcoxon rank sum test, $P = 1.69 \times 10^{-19}$).

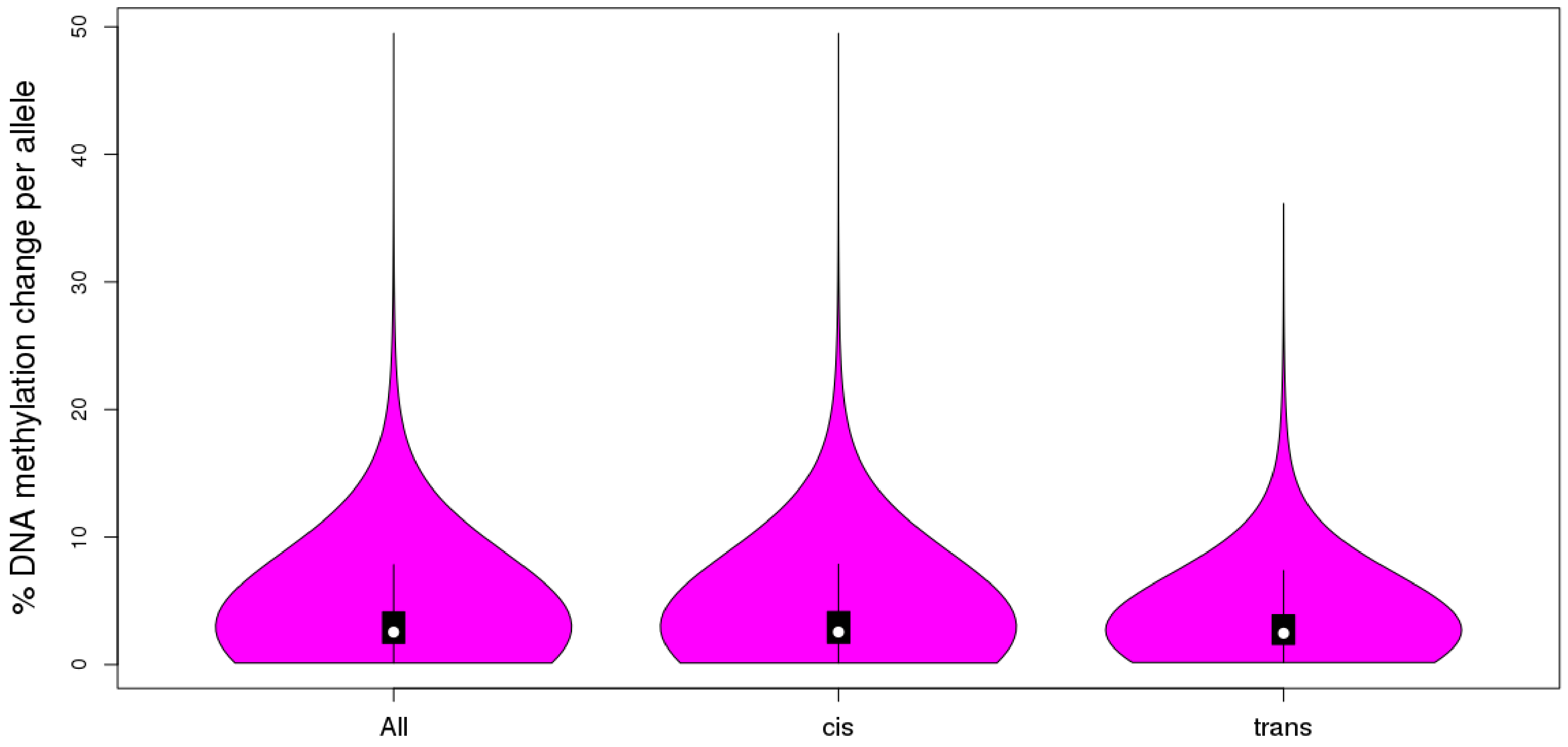

Figure S7: The significance and effect size of cis mQTL associations increases as the distance between the genetic variant and DNAm site decreases. Shown is the relationship between A) mQTL significance (–$\log_{10}$ P value) and B) the mean change in DNA methylation (%) per allele and distance between the DNA methylation site and genetic variant. The color of the points reflects the density of observations at that location, with gray indicating smallest density and red the highest.

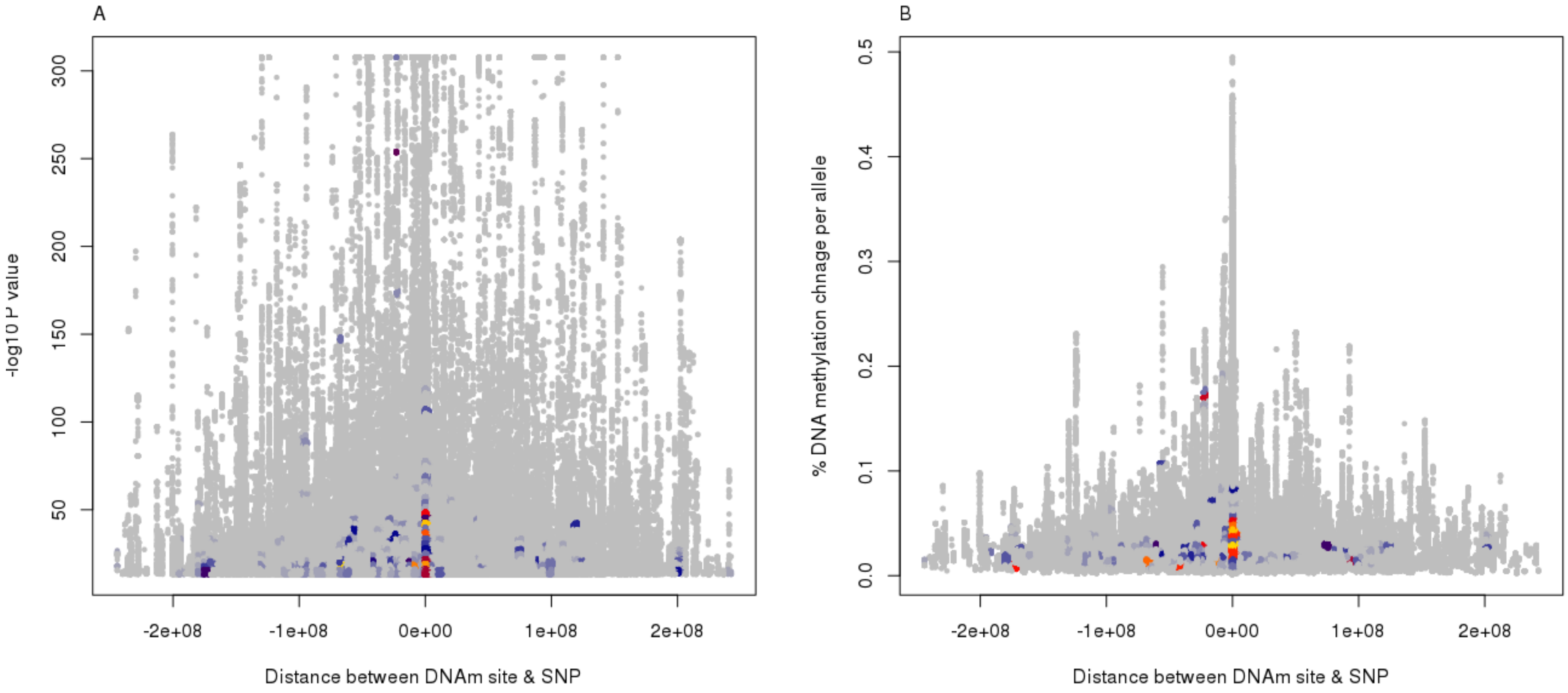

Figure S8: Genic distribution of DNA methylation sites associated with mQTL variation. Bar-plot demonstrating the genic location of DNA methylation sites associated with common genetic variation ($P < 6.52x10^{-14}$; blue bars) compared to the background distribution of all DNA methylation sites tested in the mQTL analysis (gray bars). DNA methylation sites associated with mQTLs are significantly enriched in intergenic regions, and significantly depleted in both the gene body and 1st Exon.

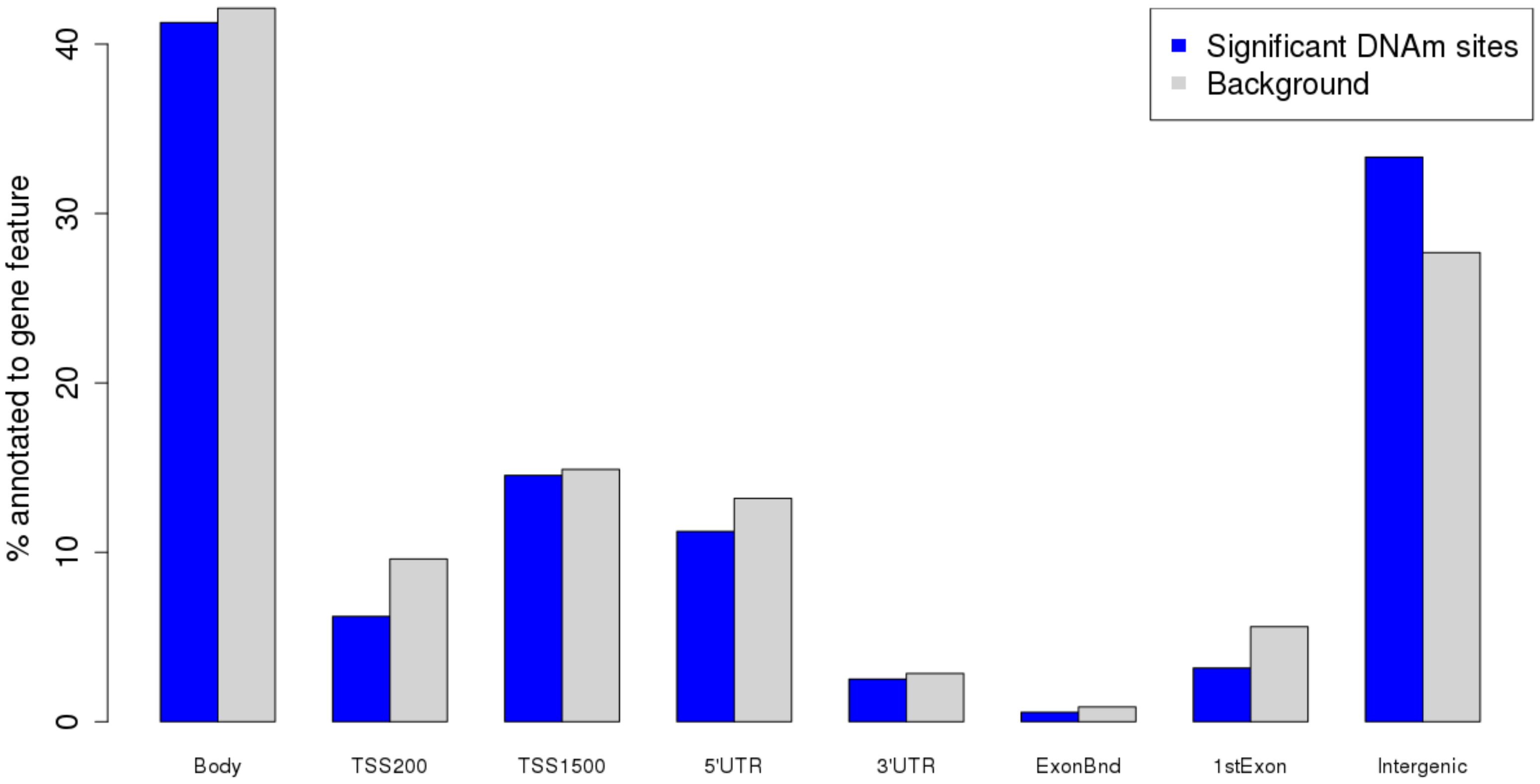

Figure S9: Distribution of DNA methylation sites associated with mQTL variation in CpG island features. Bar-plot demonstrating the genic location of DNA methylation sites associated with common genetic variation ($P < 6.52 \times 10^{-14}$; blue bars) compared to the background distribution of all DNA methylation sites tested in the mQTL analysis (gray bars). DNA methylation sites associated with mQTLs are significantly enriched in CpG shores and regions classified as "open sea", and significantly depleted in CpG islands

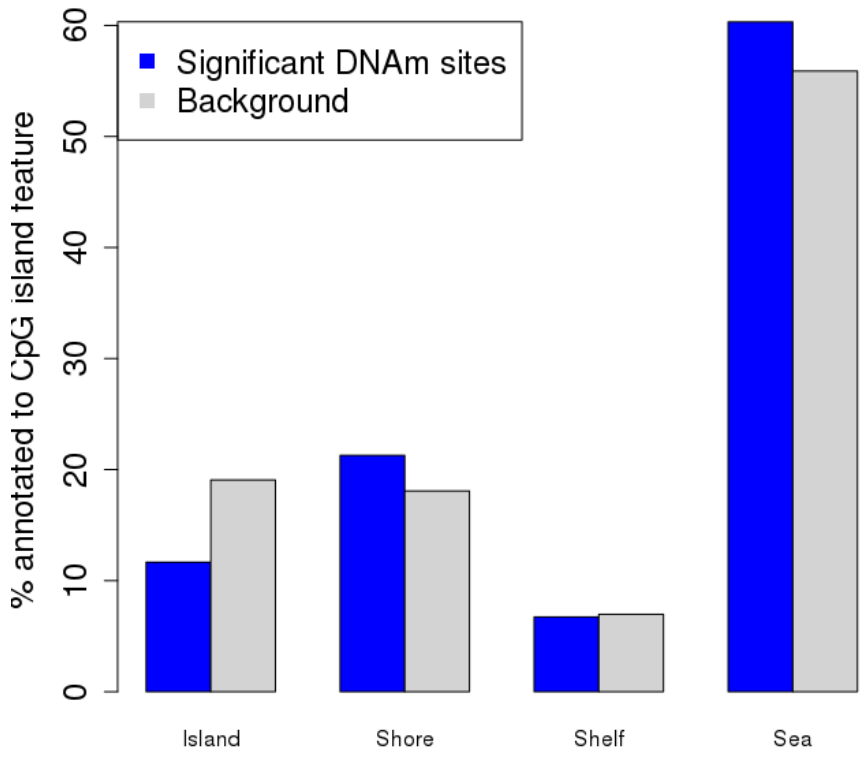

Figure S10: DNA methylation sites associated with common mQTL variants are more strongly influenced by additive genetic variation. Density plots of the distribution of percentage of variance in DNA methylation explained by genetic factors (h^2) as reported by (van Dongen et al. 2016). The distribution of all DNA methylation sites tested (black line), all DNA methylation sites with a Bonferroni significant association ($P < 6.52x10^{-14}$; red line), DNA methylation sites identified with a *cis* acing genetic association (blue line) and DNA methylation sites identified with a *trans* acting genetic association (green line). *cis* effects are defined as those where the DNA methylation site and genetic variants are located within 500kilbases on the same chromosome.

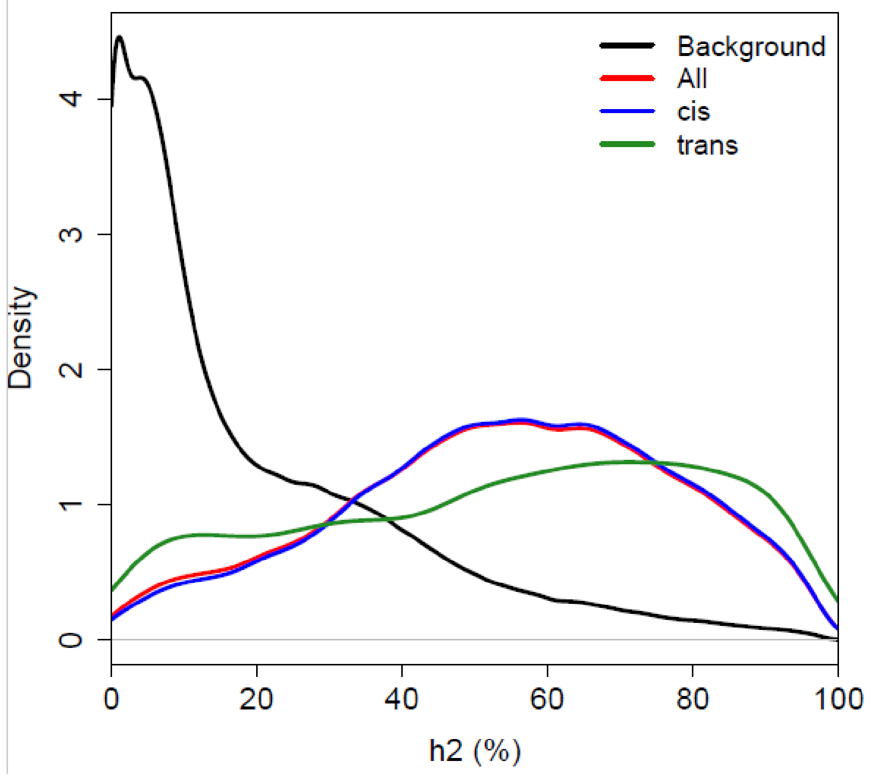

Figure S11: Distribution of correlation coefficients between pairs of DNAm sites with shared genetic effects. Histogram of correlation coefficients between pairs of DNAm sites with convincing evidence for co-localisation of genetic associations (n = 234,460 pairs with $PP_3 + PP_4 > 0.99$ & $PP_4/PP_3 > 5$). These pairs are enriched for concordant directions of effects (71.2% pairs with positive correlations vs 28.8% pairs with negative correlations, binomial test P = 1.48×10^{-323}).

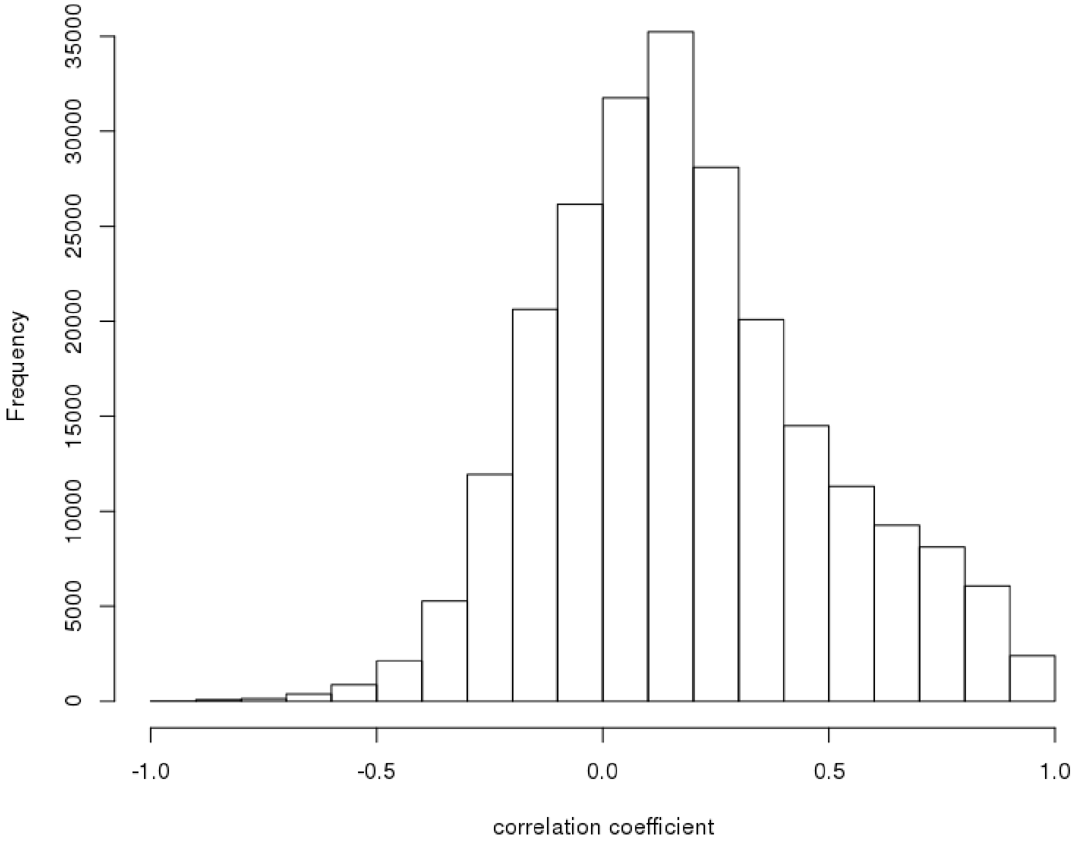

Figure S12: Distribution of correlation coefficients between pairs of DNAm sites with shared genetic effects, split by genic feature annotation. Boxplots of correlation coefficients between pairs of DNAm sites split by the genic feature annotation of the two DNAm sites in the pair for A) all pairs with convincing evidence for co-localisation of genetic associations (n = 234,460 pairs with $PP_3 + PP_4 > 0.99$ & $PP_4/PP_3 > 5$) and B) the subset of convincing pairs annotated to the same gene (n = 83,416). The colour of the boxplot indicates the genic feature category of one DNAm site in the pair and the location of the box indicates the genic feature category of the second DNam site. For example, the first boxplot on the far left of the figure presents the distribution of all pairs of DNAm sites where both sites are located in the 1st exon (indicated by the location in the 1st Exon panel and the colour red), the second yellow boxplot from the left presents the distribution of all pairs of DNAm sites where one DNAm site is located in the 1st Exon (indicated by the location in the 1st Exon panel) and the second is in the 3'UTR (indicated by the colour). In general we observe that where both DNAm sites are annotated the same genic feature the correlations are more likely to be positive (OR = 1.73, fisher's $P < 2.2x10^{-16}$) and higher (Mann-Whitney test $P < 2.2x10^{-16}$) compared to pairs annotated to different genic features.

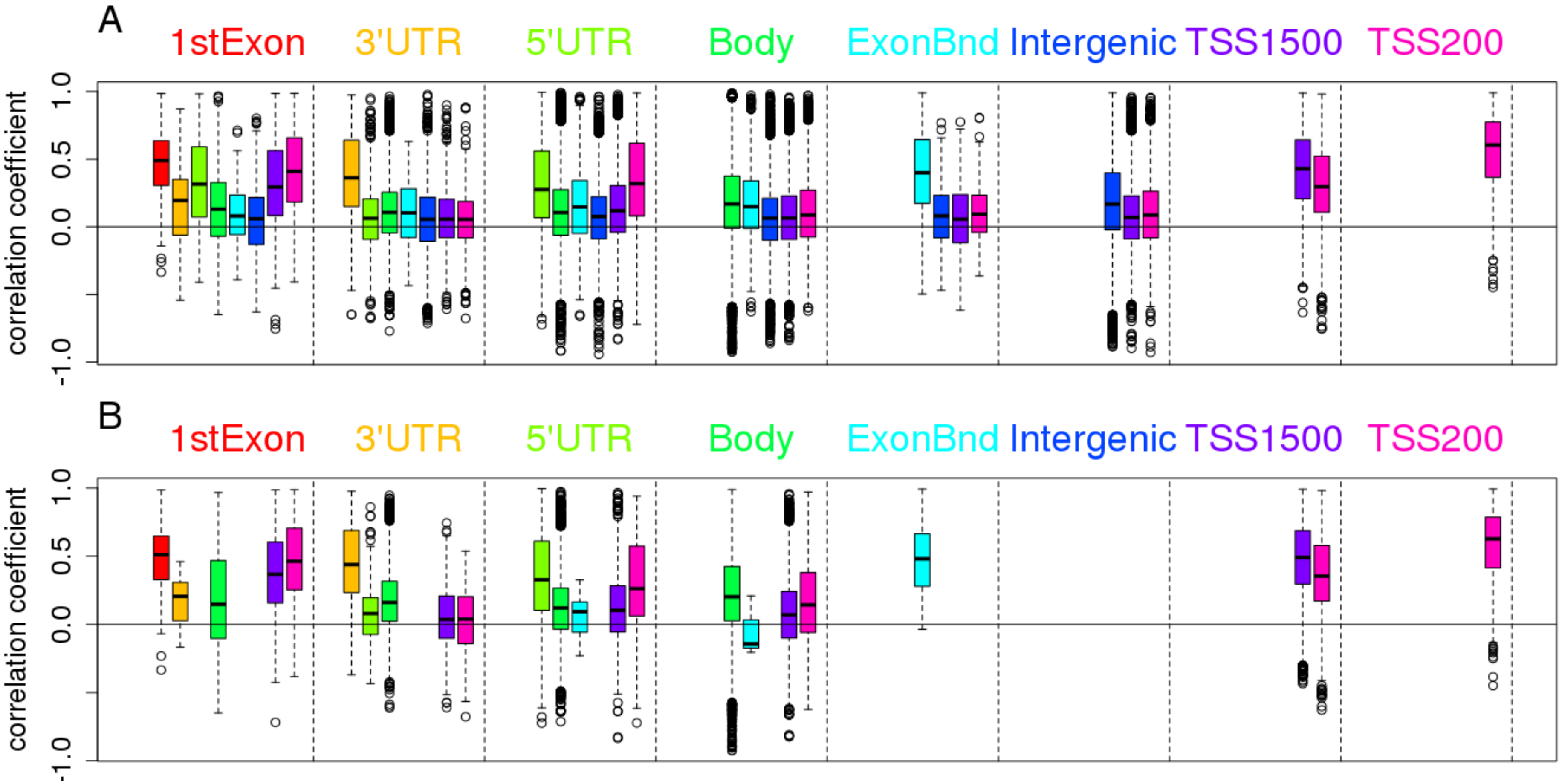
A
1stExon
3'UTR
5'UTR
Body
ExonBnd
Intergenic
TSS1500
TSS200
correlation coefficient
-1.0
0.0
0.5
1.0
B
1stExon
3'UTR
5'UTR
Body
ExonBnd
Intergenic
TSS1500
TSS200
correlation coefficient
-1.0
0.0
0.5
1.0

Figure S13: Manhattan plots of Summary data-based Mendelian Randomisation (SMR) tests for pleiotropic effects between 63 complex traits and DNA methylation. Shown on the y-axis of each plot is the –log10 P-value from the SMR analysis using DNA methylation quantitative trait loci (mQTL) generated from whole blood. Each point represents an SMR test for a particular DNA methylation site. The red horizontal line represents the genome-wide multiple testing significance threshold ($P < 6.42 \times 10^{-7}$); green points highlight the significant SMR tests which are not characterized by significant heterogeneity (i.e. $P > 0.05$), indicating pleiotropic relationships between that trait and either DNA methylation.

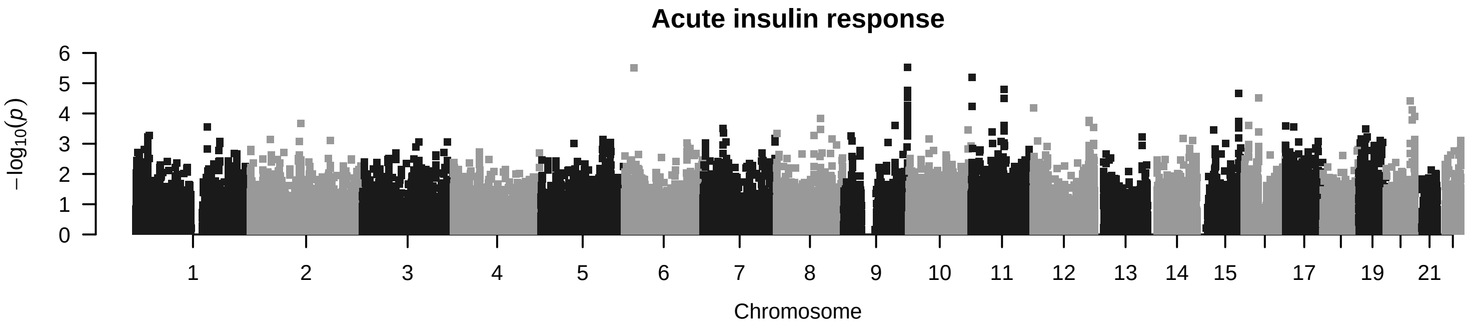
Acute insulin response
−log10(p)
0
1
2
3
4
5
6
1
2
3
4
5
6
7
8
9
10
11
12
13
14
15
17
19
21
Chromosome

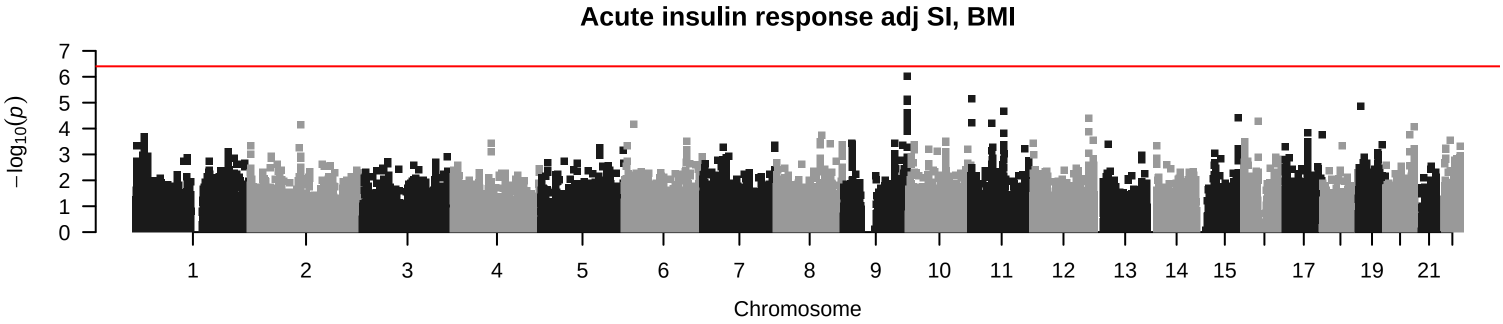
Acute insulin response adj SI, BMI
−log10(p)
0
1
2
3
4
5
6
7
1
2
3
4
5
6
7
8
9
10
11
12
13
14
15
17
19
21
Chromosome

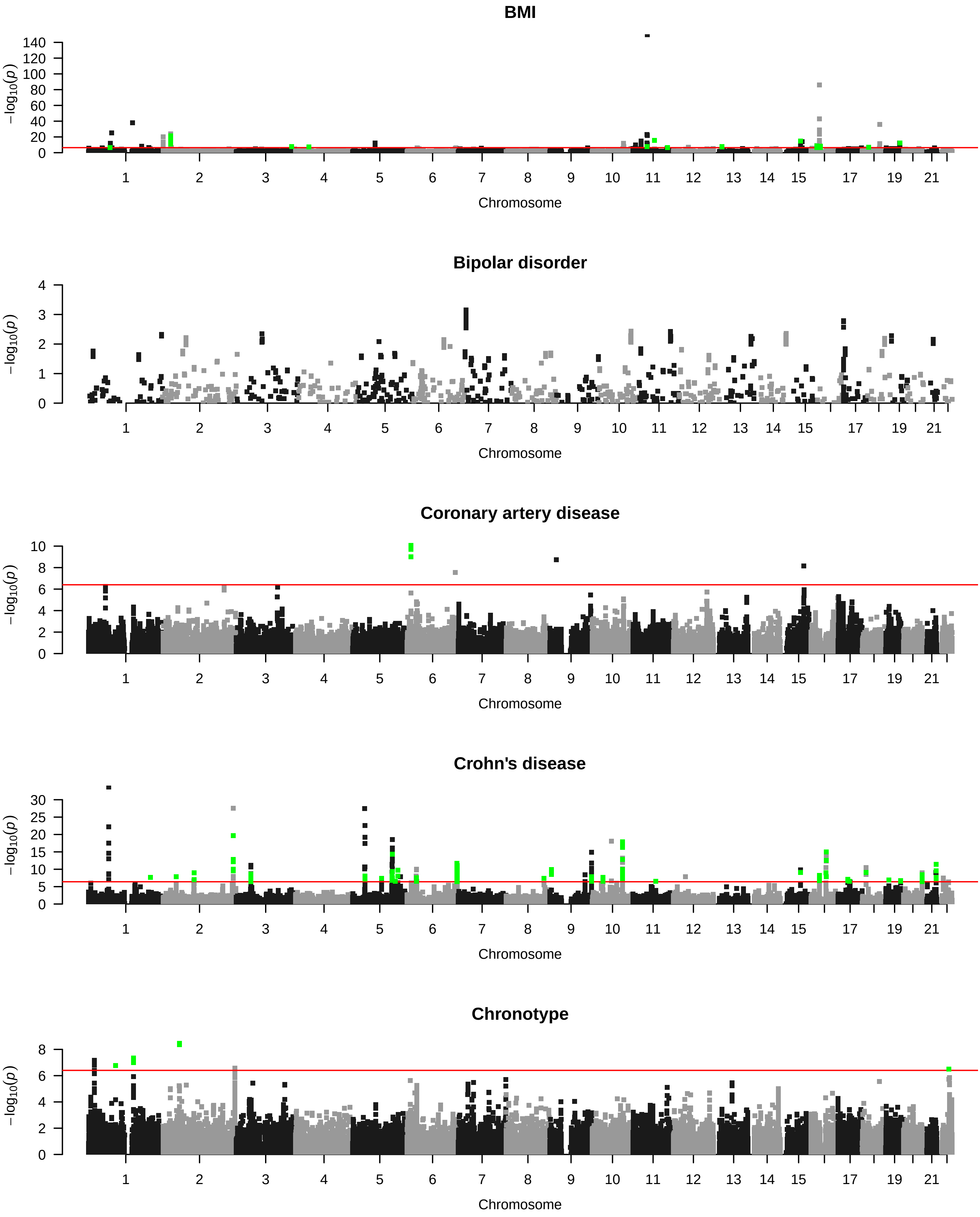
BMI
Bipolar disorder
Coronary artery disease
Crohn's disease
Chronotype
$-\log_{10}(p)$
Chromosome
1
2
3
4
5
6
7
8
9
10
11
12
13
14
15
17
19
21

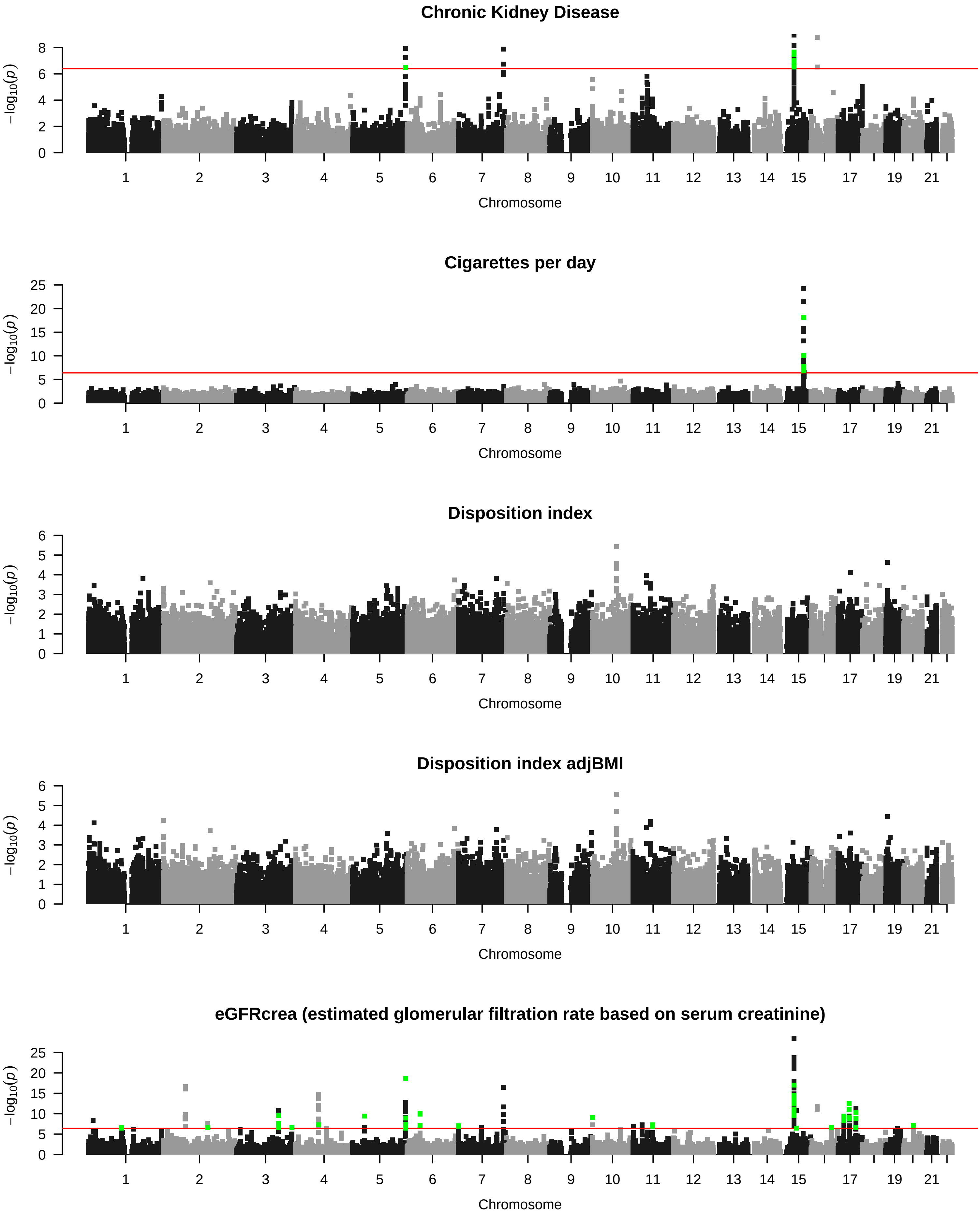

Chronic Kidney Disease
Cigarettes per day
Disposition index
Disposition index adjBMI
eGFRcrea (estimated glomerular filtration rate based on serum creatinine)
$-\log_{10}(p)$
Chromosome
1 2 3 4 5 6 7 8 9 10 11 12 13 14 15 17 19 21

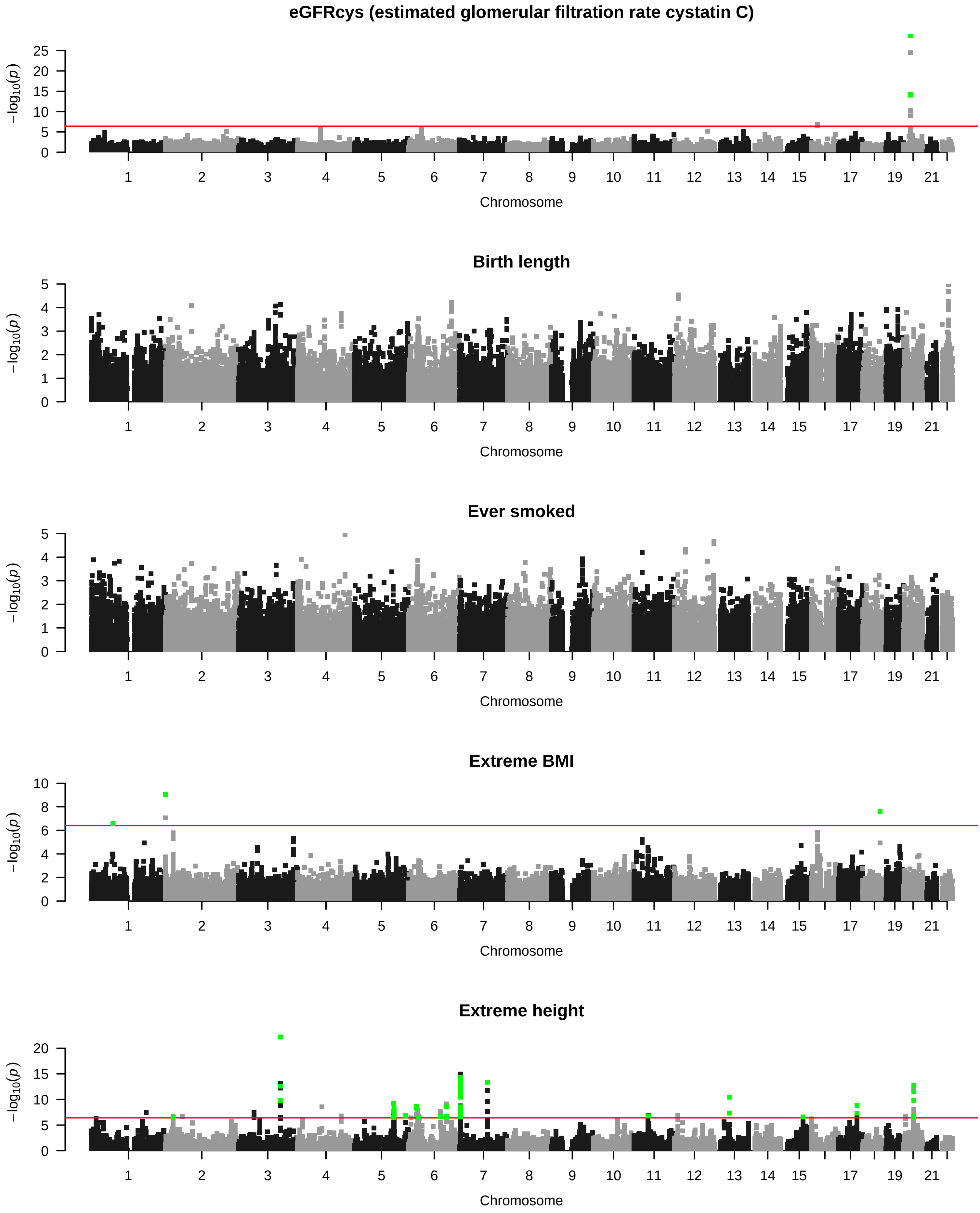
eGFRcys (estimated glomerular filtration rate cystatin C)
Birth length
Ever smoked
Extreme BMI
Extreme height
$-\log_{10}(p)$
Chromosome
1
2
3
4
5
6
7
8
9
10
11
12
13
14
15
17
19
21

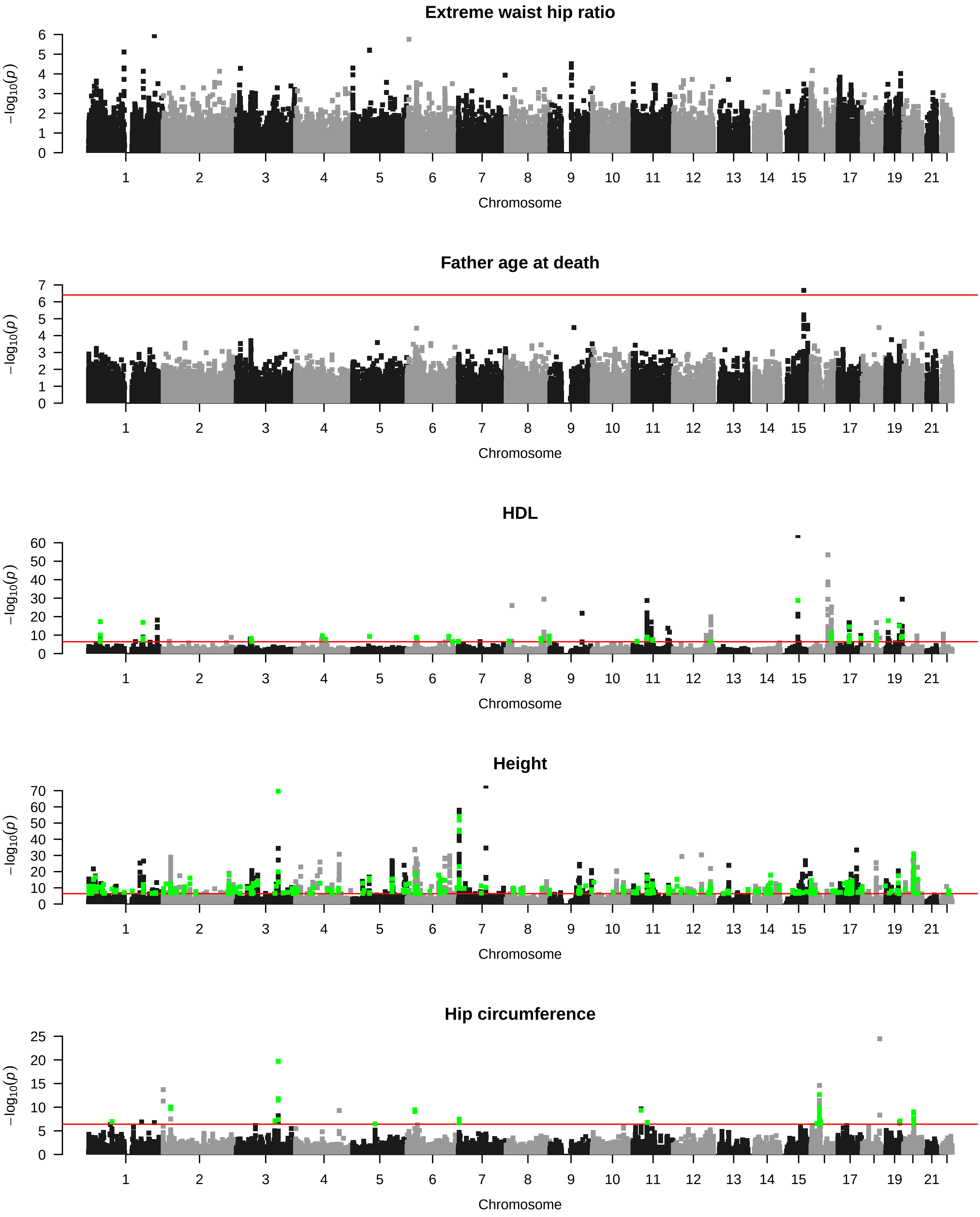
Extreme waist hip ratio
Father age at death
HDL
Height
Hip circumference
−log10(p)
Chromosome
1
2
3
4
5
6
7
8
9
10
11
12
13
14
15
17
19
21

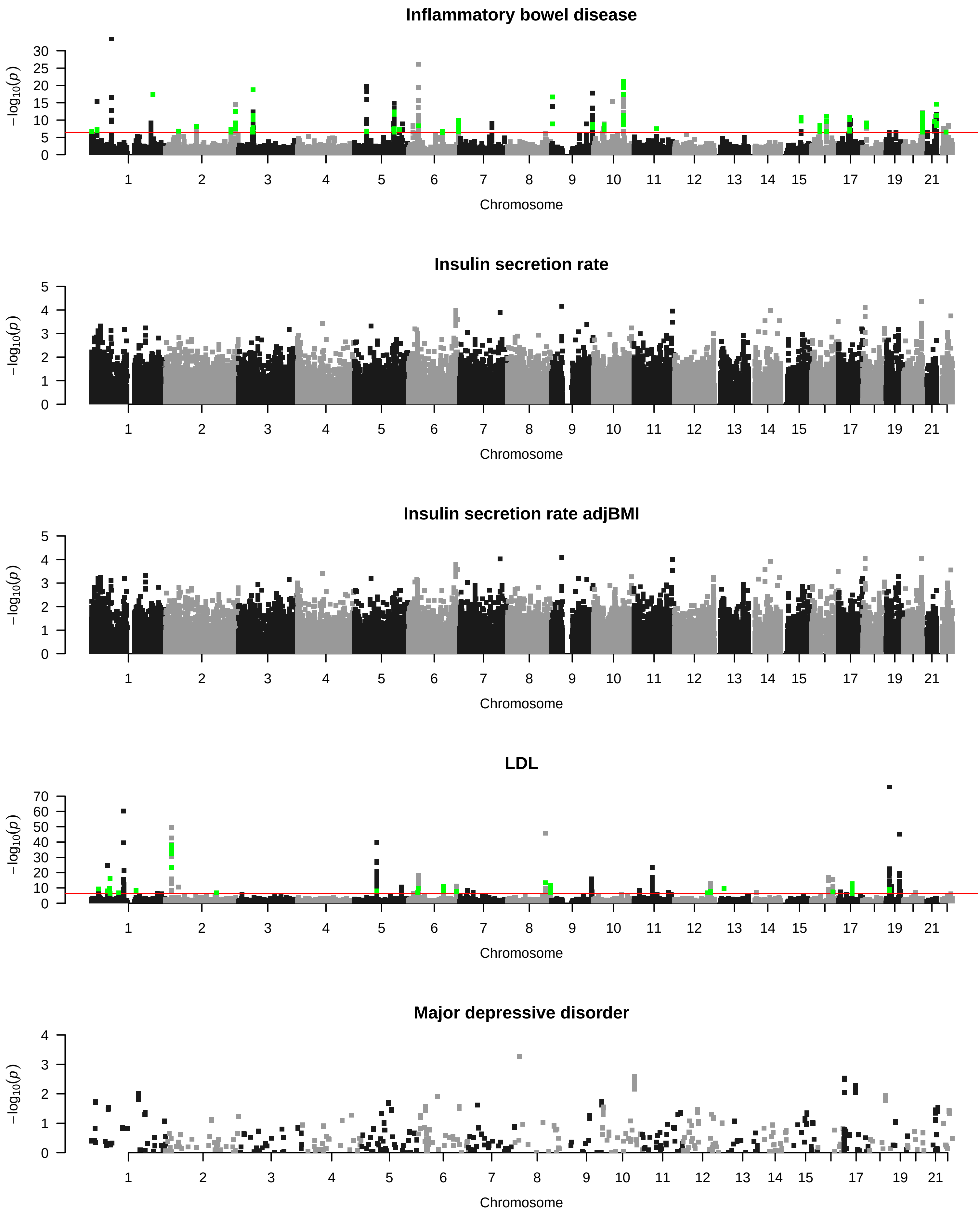

Inflammatory bowel disease
Insulin secretion rate
Insulin secretion rate adjBMI
LDL
Major depressive disorder
$-\log_{10}(p)$
Chromosome
1
2
3
4
5
6
7
8
9
10
11
12
13
14
15
17
19
21

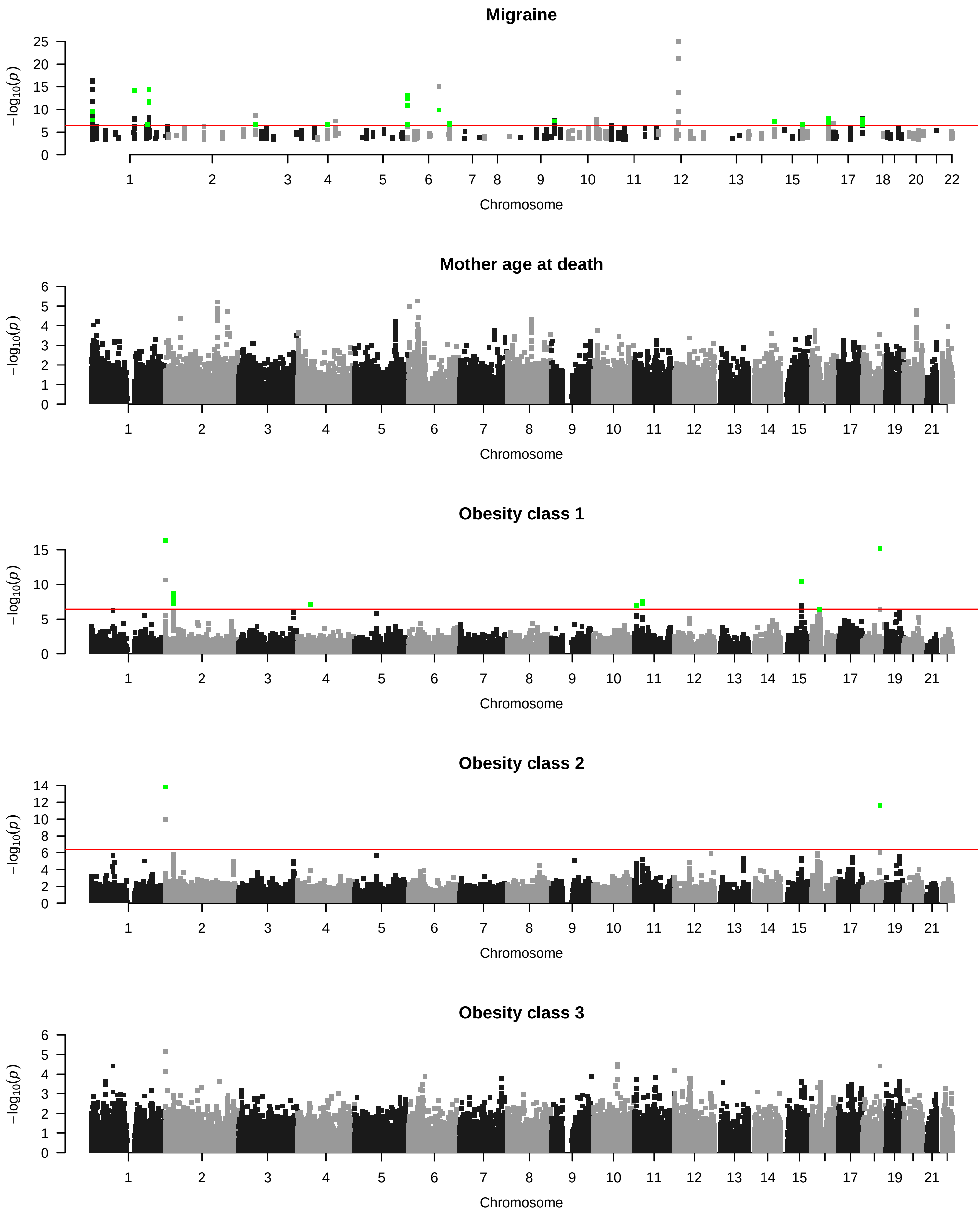
Migraine
−log10(p)
0
5
10
15
20
25
Chromosome
Mother age at death
Obesity class 1
Obesity class 2
Obesity class 3

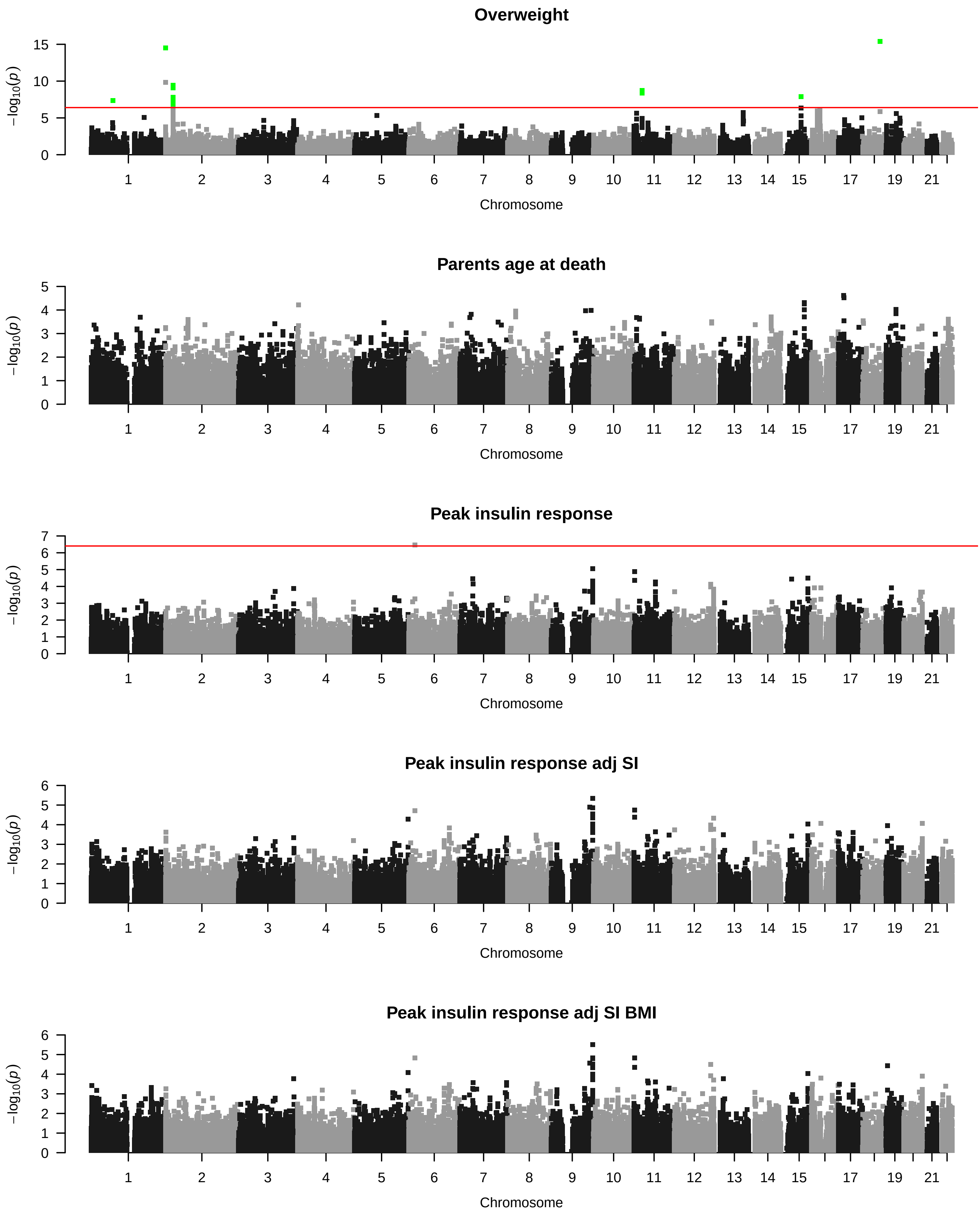

Overweight
$-\log_{10}(p)$
0
5
10
15
Chromosome
1
2
3
4
5
6
7
8
9
10
11
12
13
14
15
17
19
21
Parents age at death
$-\log_{10}(p)$
0
1
2
3
4
5
Chromosome
Peak insulin response
$-\log_{10}(p)$
0
1
2
3
4
5
6
7
Chromosome
Peak insulin response adj SI
$-\log_{10}(p)$
0
1
2
3
4
5
6
Chromosome
Peak insulin response adj SI BMI
$-\log_{10}(p)$
0
1
2
3
4
5
6
Chromosome

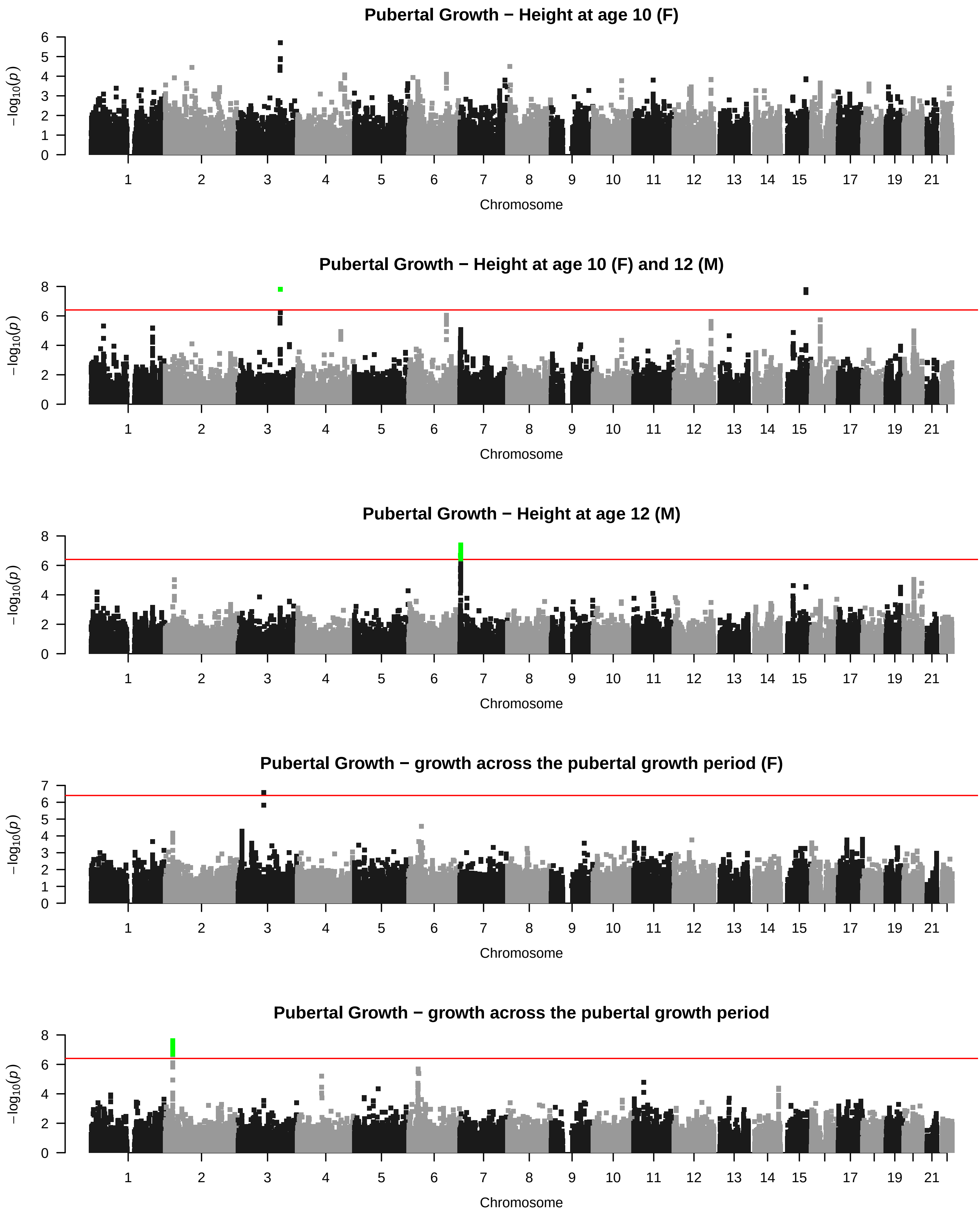
Pubertal Growth − Height at age 10 (F)
Pubertal Growth − Height at age 10 (F) and 12 (M)
Pubertal Growth − Height at age 12 (M)
Pubertal Growth − growth across the pubertal growth period (F)
Pubertal Growth − growth across the pubertal growth period
$-\log_{10}(p)$
Chromosome
1 2 3 4 5 6 7 8 9 10 11 12 13 14 15 17 19 21

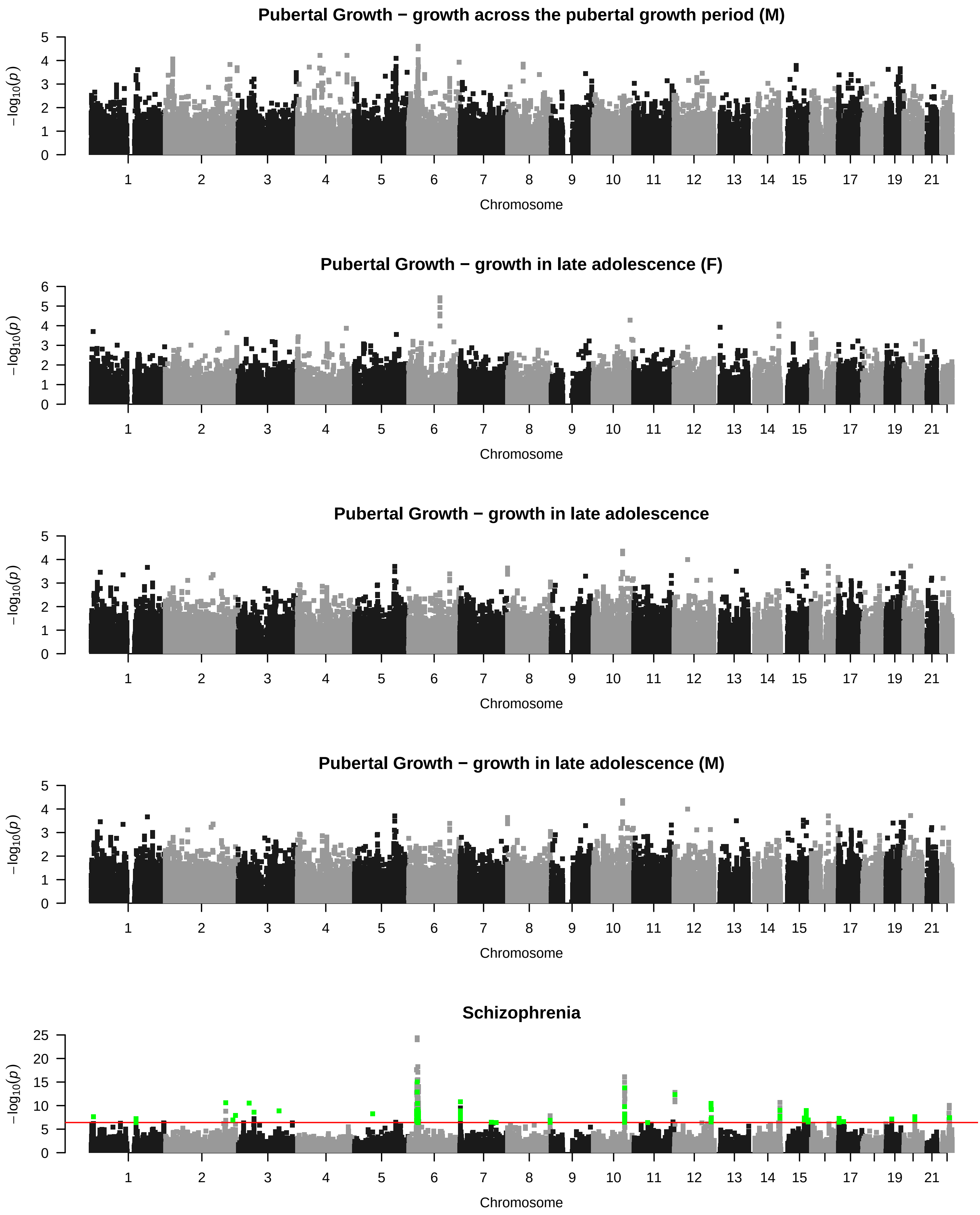

Pubertal Growth − growth across the pubertal growth period (M)
−log10(p)
Chromosome
Pubertal Growth − growth in late adolescence (F)
−log10(p)
Chromosome
Pubertal Growth − growth in late adolescence
−log10(p)
Chromosome
Pubertal Growth − growth in late adolescence (M)
−log10(p)
Chromosome
Schizophrenia
−log10(p)
Chromosome

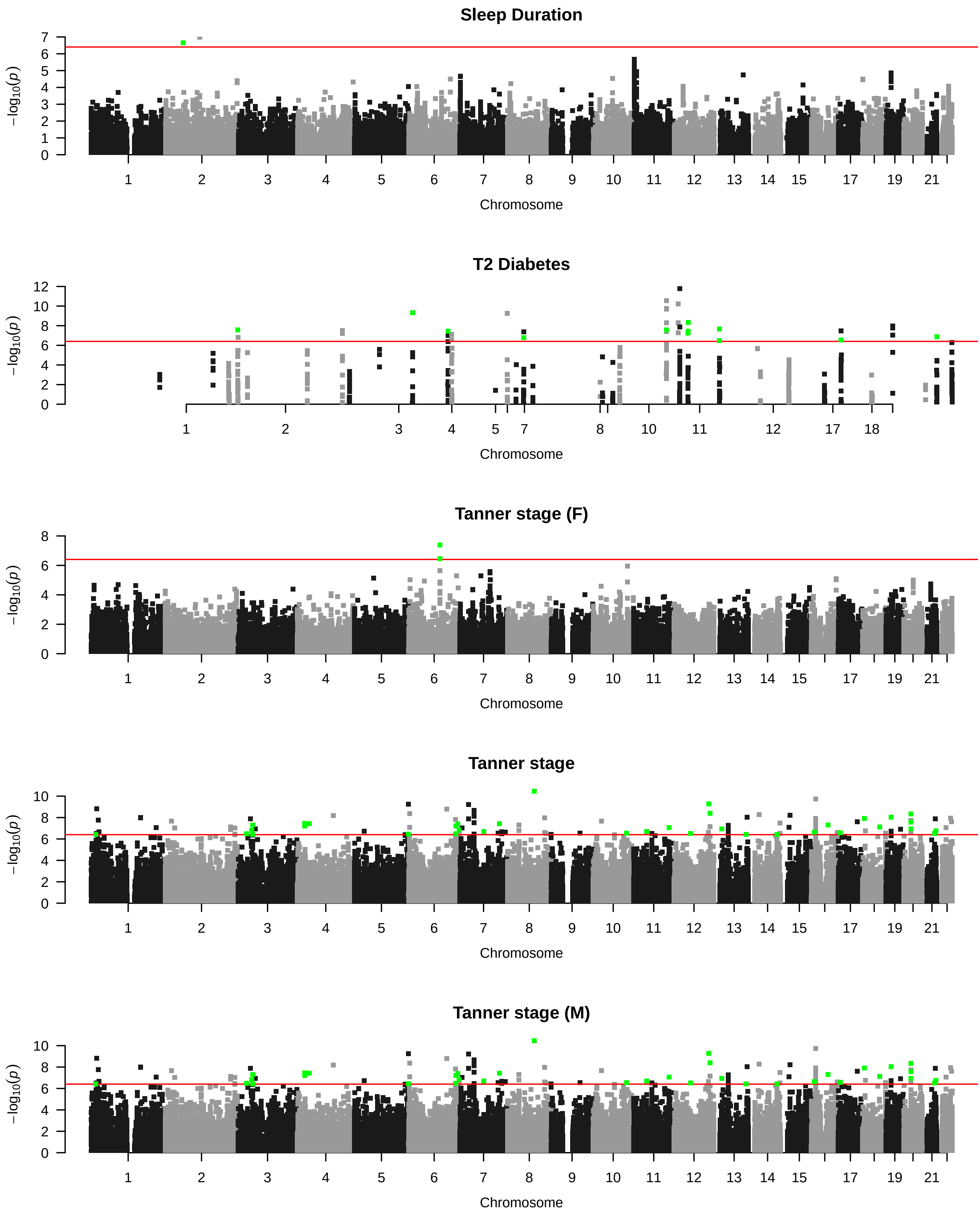
Sleep Duration
−log10(p)
Chromosome
T2 Diabetes
−log10(p)
Chromosome
Tanner stage (F)
−log10(p)
Chromosome
Tanner stage
−log10(p)
Chromosome
Tanner stage (M)
−log10(p)
Chromosome

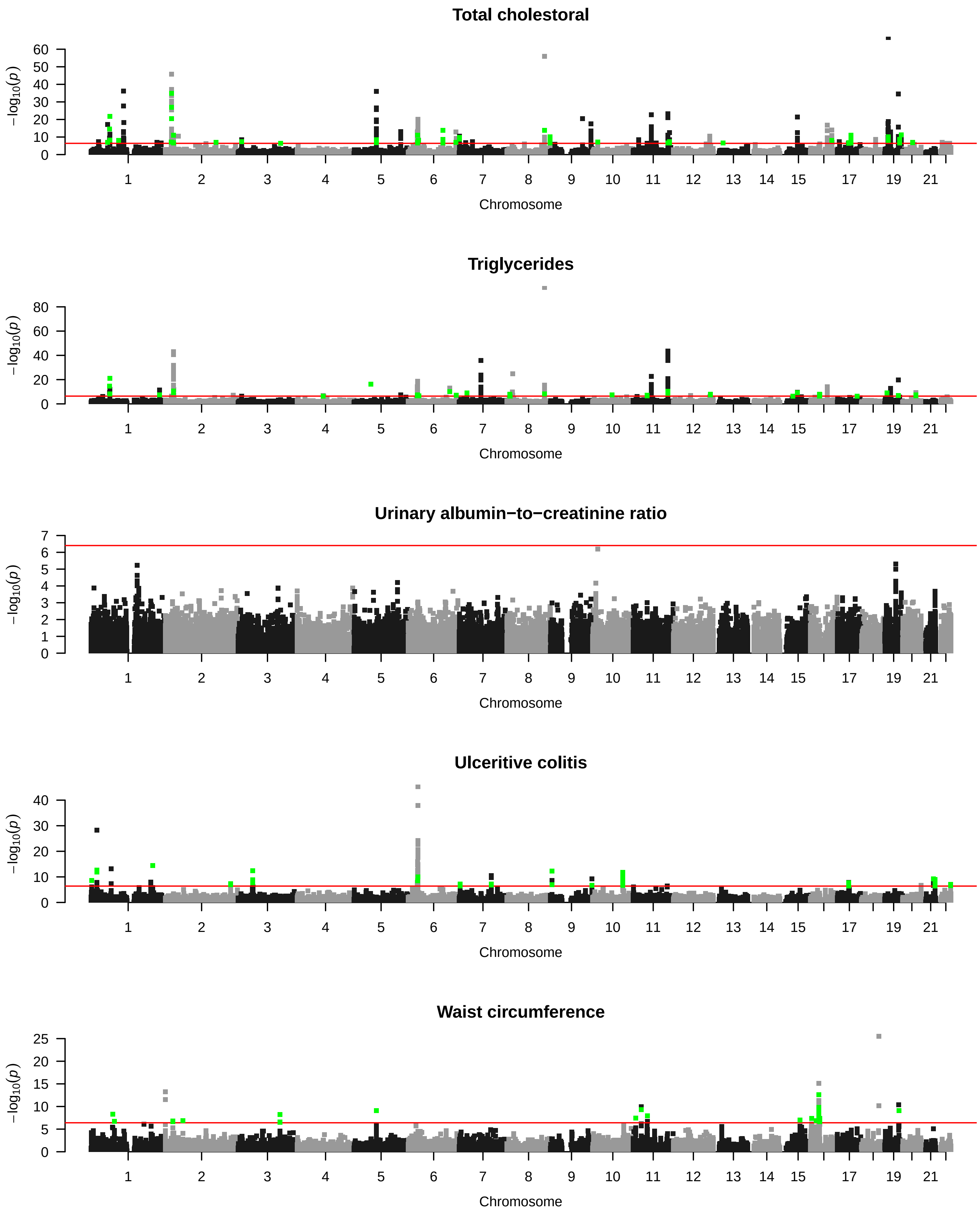
Total cholestoral
Triglycerides
Urinary albumin−to−creatinine ratio
Ulceritive colitis
Waist circumference
$-\log_{10}(p)$
Chromosome
1 2 3 4 5 6 7 8 9 10 11 12 13 14 15 17 19 21

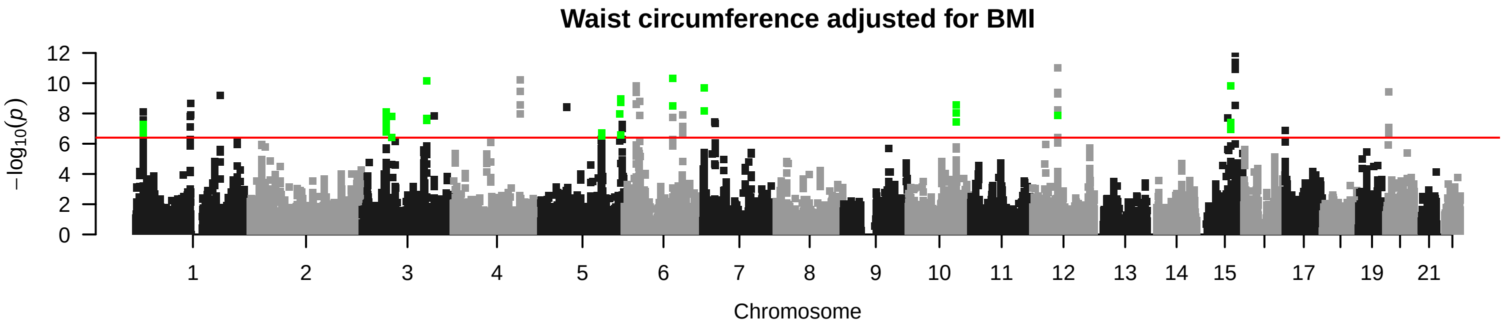
Waist circumference adjusted for BMI
−log10(p)
Chromosome

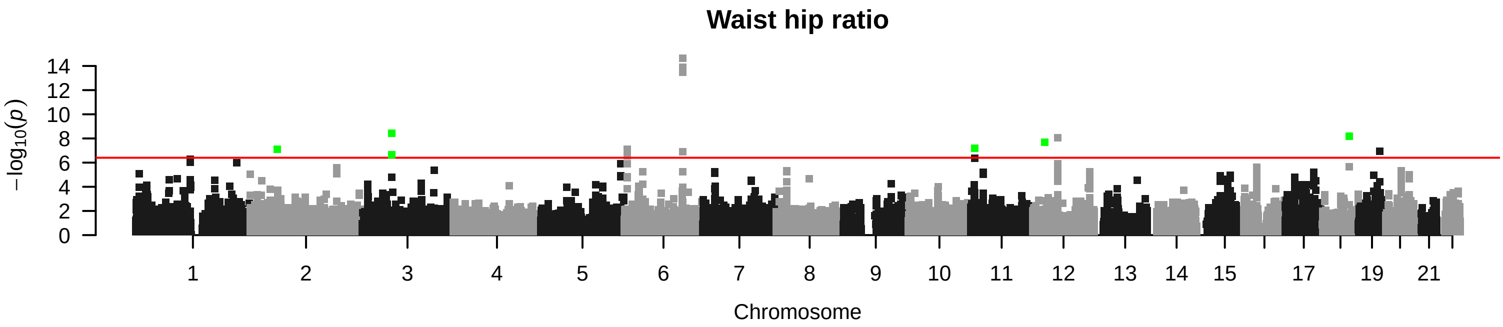
Waist hip ratio
−log10(p)
Chromosome

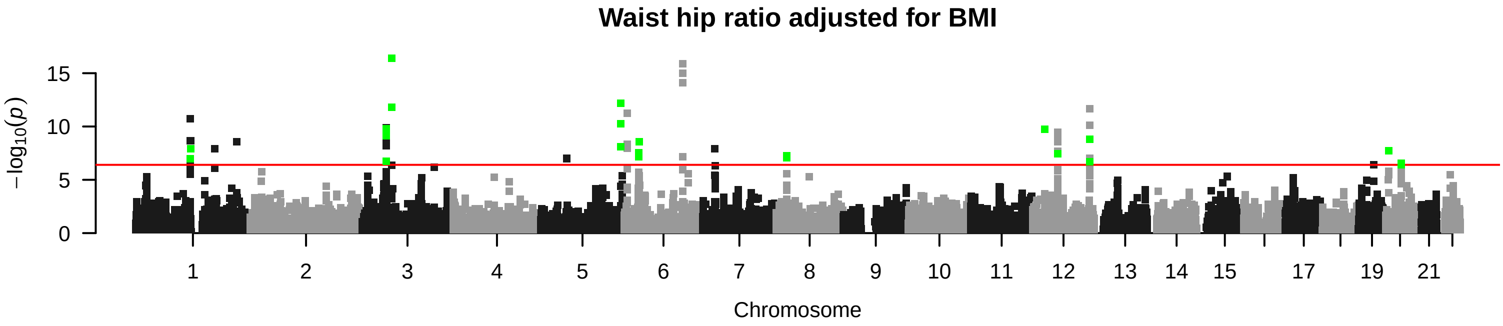
Waist hip ratio adjusted for BMI
−log10(p)
Chromosome

Figure S14: Replication of significant pleiotropic associations across two independent datasets. Scatterplots of estimated causal effect from Summary data-based Mendelian Randomization (SMR) analysis between the UKHLS (x-axis) and our previous study (y-axis)(Hannon et al. 2017). Each point represents a significant pleiotropic associations identified in UKHLS also tested in the replication dataset. Green points indicate associations significant in both datasets.

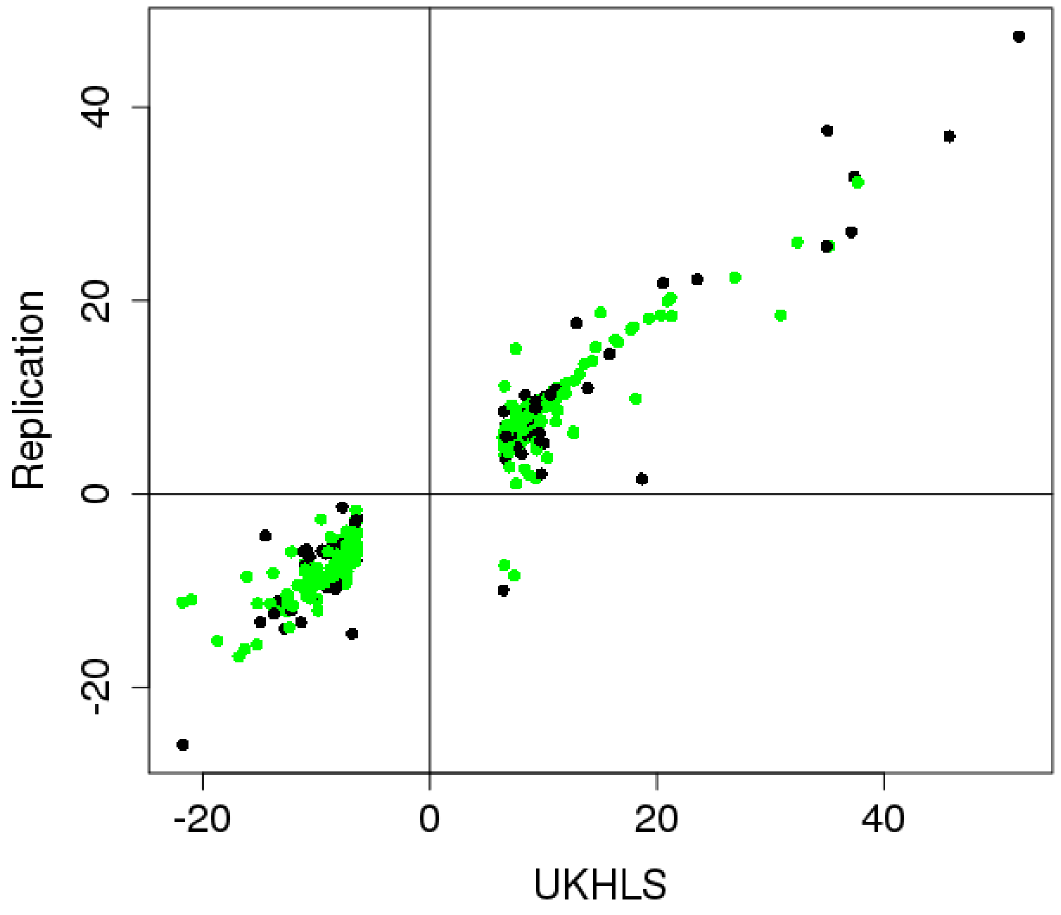

Figure S15: Flowchart showing the number of gene expression probes, DNAm sites and pairs significant at each stage of the analysis.

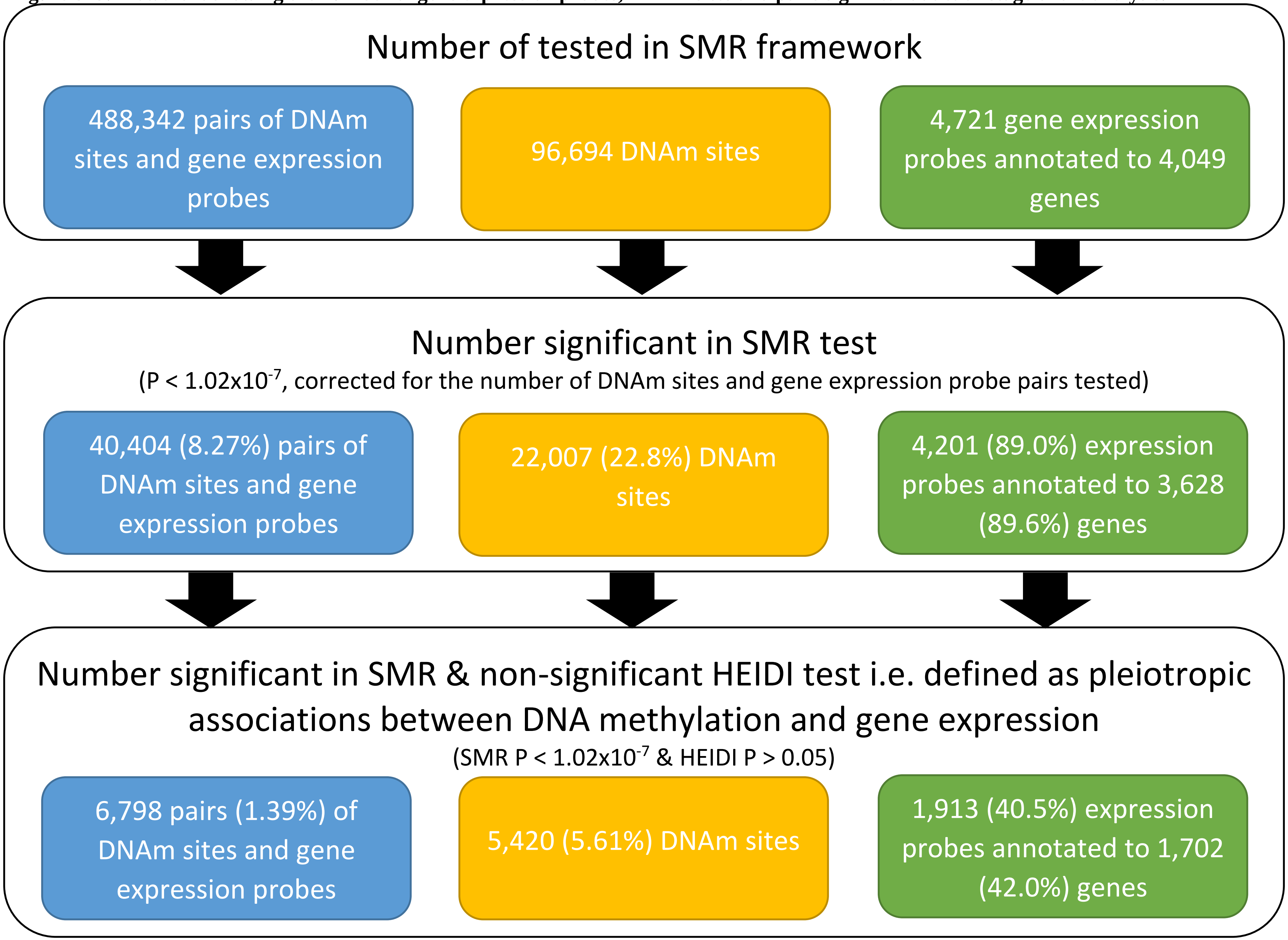

Figure S16: Genomic distribution of DNA methylation sites pleiotropically associated with gene expression. Barplot demonstrating the genic location of DNA methylation sites pleiotropically associated with gene expression (blue bars) compared to the background rate inferred from all DNA methylation sites included in this analysis (gray bars). There was enrichment for DNA methylation sites to be located in the gene body and promotor (TSS1500, TSS200, 5'UTR) and depleted in intergenic regions, see **Table S9** for frequency statistics.

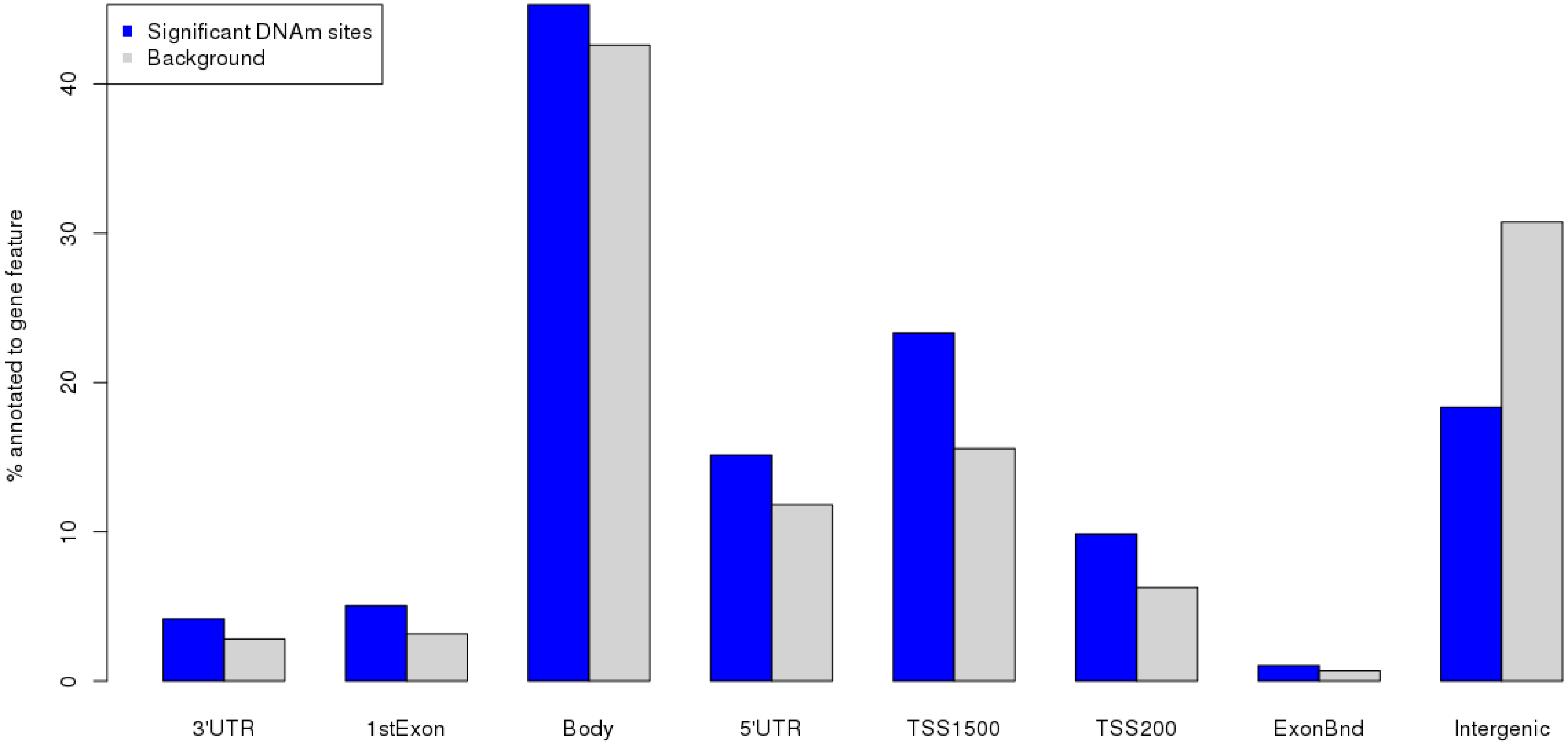

Figure S17: Distribution of estimated effect of pleiotropic associations between DNA methylation and gene expression, stratified by genic location of DNA methylation site. Violin plots of effect size estimated from the SMR analysis between DNA methylation and gene expression. Each violin plot represents pleiotropic associations where the DNA methylation site is annotated to that genic feature. All features are associated with both positive and negative relationships with gene expression, however there is a significant shift towards negative associations for 1st Exon (P = 6.19e-05) 5'UTR (P = 0.00108), TSS200 (P = 6.38e-07), TSS1500 (P = 5.82e-11), gene body (P = 0.0230).

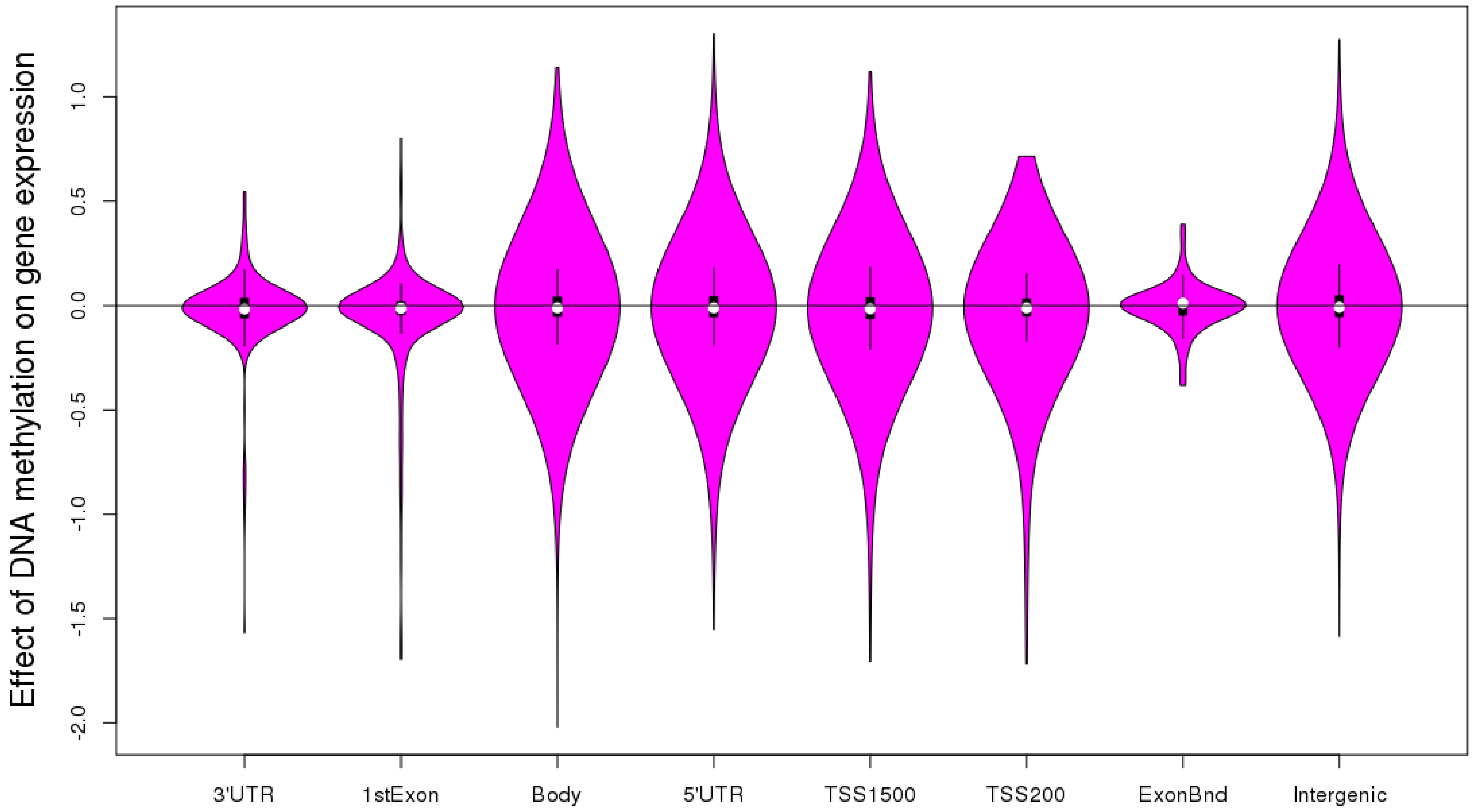

Table S1: Summary of DNA methylation quantitative trait loci analysis in Understanding Society (n = 1,111). A) all mQTL; B) mQTL classed as cis (distance between genetic variant and DNA methylation site < 500kb); C) mQTL classed as trans.

A)

P value threshold	nMQTL	nSNPs	nProbes	DNA methylation change per allele	
				mean	SD
6.52E-14	12689548	2907234	93268	3.46%	3.01%
1.00E-13	12886785	2926784	94209	3.44%	3.00%
1.00E-12	14040864	3036038	99833	3.31%	2.93%
1.00E-11	15399232	3152092	106408	3.18%	2.85%
1.00E-10	17051673	3281391	114595	3.03%	2.76%

B)

P value threshold	nMQTL	nSNPs	nProbes	DNA methylation change per allele	
				mean	SD
6.52E-14	11679376	2837748	89325	3.48%	3.03%
1.00E-13	11854125	2857053	90197	3.46%	3.02%
1.00E-12	12866269	2964911	95439	3.33%	2.95%
1.00E-11	14055426	3079629	101414	3.20%	2.87%
1.00E-10	15479136	3204842	108405	3.05%	2.79%

C)

P value threshold	nMQTL	nSNPs	nProbes	DNA methylation change per allele	
				mean	SD
6.52E-14	1010172	398965	7791	3.26%	2.78%
1.00E-13	1032660	404570	7948	3.23%	2.76%
1.00E-12	1174595	438405	8935	3.10%	2.66%
1.00E-11	1343806	478896	10224	2.98%	2.56%
1.00E-10	1572537	531470	12347	2.83%	2.45%

Table S2: Summary of additional associations from novel content on Illumina EPIC BeadArray compared to Illumina 450K BeadArray.

P value threshold	DNAm probes associated with an mQTL				Genes associated with an mQTL			Intergenic DNA methylation sites associated with an mQTL	
	All	EPIC specific			All	Only associated with EPIC specific	Additional association with EPIC of 450K association	All	EPIC specific
		Total	within 1kb of 450k association	within 5kb of 450k association					
6.52E-14	93268	48099	8509	15627	16276	5172	6521	31087	17780
1.00E-13	94209	48593	8613	15809	16360	5185	6577	31408	17955
1.00E-12	99833	51407	9180	17020	16802	5211	6905	33310	19010
1.00E-11	106408	54715	9931	18513	17266	5235	7277	35399	20185
1.00E-10	114595	58884	10929	20451	17848	5268	7711	38063	21677

Table S3: Frequency of DNAm sites in A) genic feature and B) CpG Island feature annotation categories.

A)

Genic annotation category	DNAm sites associated with mQTL (P < 6.52x10-14)		All tested DNAm sites	
	Number	%	Number	%
**Body**	38495	41.27353433	330180	42.11656868
**TSS200**	5805	6.223999657	75322	9.607802369
**TSS1500**	13567	14.54625381	116805	14.89922407
**5'UTR**	10478	11.23429258	103362	13.18448353
**3'UTR**	2349	2.518548698	22363	2.852543538
**ExonBnd**	524	0.561821847	6857	0.874654163
**1stExon**	2963	3.176866664	43993	5.611588243
**Intergenic**	31087	33.33083158	217106	27.6932575

B)

CpG island annotation category	DNAm sites associated with mQTL (P < 6.52x10-14)		All tested DNAm sites	
	Number	%	Number	%
**Island**	10878	11.66316421	149471	19.06598109
**Shore**	19850	21.28275507	141724	18.07780174
**Shelf**	6278	6.73114037	54623	6.967512663
**Sea**	56262	60.32294034	438149	55.8887045

Table S9: Frequency of DNAm sites associated with gene expression in gene feature annotation categories.

Genic annotation category	DNAm sites associated with gene expression (SMR P < 1.02x10-7 & HEIDI P > 0.05)		All DNAm sites tested against gene expression	
	Number	%	Number	%
3'UTR	226	4.169741697	2728	2.821271227
1stExon	274	5.055350554	3044	3.148075372
Body	2456	45.31365314	41186	42.59416303
5'UTR	820	15.12915129	11425	11.81562455
TSS1500	1263	23.30258303	15063	15.57800898
TSS200	533	9.833948339	6050	6.256851511
ExonBnd	56	1.033210332	668	0.690839142
Intergenic	994	18.33948339	29727	30.74337601

References

Hannon E, Weedon M, Bray N, O'Donovan M, Mill J. 2017. Pleiotropic Effects of Trait-Associated Genetic Variation on DNA Methylation: Utility for Refining GWAS Loci. *Am J Hum Genet* doi:10.1016/j.ajhg.2017.04.013.

van Dongen J, Nivard MG, Willemsen G, Hottenga JJ, Helmer Q, Dolan CV, Ehli EA, Davies GE, van Iterson M, Breeze CE et al. 2016. Genetic and environmental influences interact with age and sex in shaping the human methylome. *Nat Commun* **7**: 11115.
